# Toward Coordination Cages with Hybrid Chirality: Amino Acid-Induced Chirality on Metal Centers

**DOI:** 10.1021/acs.inorgchem.2c01738

**Published:** 2022-07-11

**Authors:** Marcin Grajda, Grzegorz Staros, Hanna Jędrzejewska, Agnieszka Szumna

**Affiliations:** † Institute of Organic Chemistry, Polish Academy of Sciences, Kasprzaka 44/52, 01-224 Warsaw, Poland

## Abstract

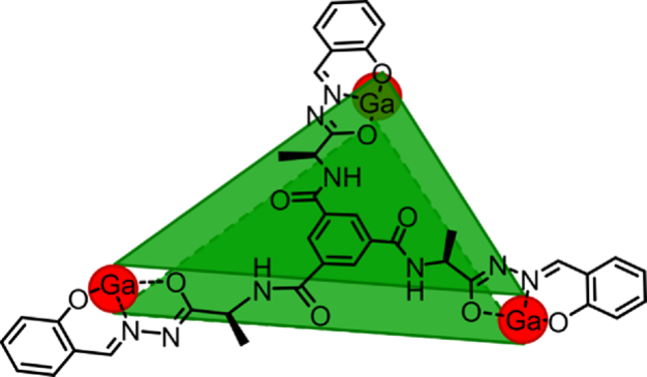

Tripodal chiral ligands containing amino acid residues and salicyl-acylhydrazone units were synthesized and used to obtain coordination cages through deprotonation and coordination to gallium. These coordination cages have Ga_3_L_2_ stoichiometry and pinwheel geometry with two types of chiral centers built into their walls: stereogenic centers at the amino acid backbones and stereoselectively induced centers at metal ions. The pinwheel geometry is unique among analogous cages and originates from the partial flexibility of the ligands. Despite the flexibility, the ligands induce the chirality of metal centers in a highly stereoselective way, leading to the formation of cages that are single diastereoisomers. It has also been demonstrated that stereoselectivity is a unique feature of cage geometry and leads to effective chiral self-sorting: homochiral cages can be obtained selectively from the mixtures of racemic ligands. The configuration of metal centers was determined by circular dichroism, TD DFT calculation, and X-ray crystallography.

## Abstract 2

Tridentate ligands containing chiral amino acids were used to synthesize coordination cages with gallium ions. Chiral induction on metal centers and chiral self-sorting were observed.

## Introduction

Metal–organic coordination cages^1^ are well-known discrete coordination structures with numerous applications that originate from their porous structure. Selective encapsulation of guest molecules,^2^ including natural products and drugs,^3^ anion extraction,^4^ protein folding,^5^ and catalysis^6^ were reported to take place in metal–organic coordination cages. For prospective applications like the asymmetric catalysis, recognition, and separation of enantiomers, the chirality of the cages is a desired feature. However, the synthesis of chiral, enantiopure coordination cages is challenging because of the requirement of precise coordination geometry and the prevention of collapse, which imposes rigidity constraints on the ligands. Therefore, carbon stereogenic centers (C-SCs), which are the most common chirality elements but contain rotatable single bonds, are rarely used to construct cores of chiral cages.^7^ In contrast, metal stereogenic centers (M-SCs) offer a unique possibility of obtaining chiral cages while maintaining rigidity.^8^ However, M-SCs still need to be induced by other chirality elements; therefore, the most common strategy for obtaining chiral cages involves the induction of chirality on M-SCs by C-SCs that are positioned externally to the basal cage skeleton.^9^ Here, we report a different approach that involves the formation of chiral coordination cages with both types of chirality elements constituting integral parts of the cores: C-SCs come from amino-acid-containing ligands, and M-SCs come from chiral Ga^III^ centers.

Achiral Ga^III^ cages of tetrahedral, octahedral, or cubic geometry have been previously obtained using various polyphenolic ligands (vase-shaped pyrogallol[4]arenes,^10^ linear catecholates,^11^ linear and trigonal acylhydrazone catecholates,^12^ and salicyl acylhydrazones^13^) and used as nanovessels and catalysts in various reactions.^14^ Chiral Ga^III^ cages, in which the chirality of M-SCs is induced by externally placed chiral amine groups, have also been reported.^15^ However, to date, the induction of chirality in Ga^III^ centers by amino acid derivatives remains unknown. Interestingly, the induction of the chirality of M-SCs by amino acids and their derivatives, despite their availability, chirality, and presence of various functional side groups, remains rare also for other metals. Sparse examples include the induction of chirality at octahedral Ni^II^ centers with l-asparagine,^16^ Co^III^ centers with amino acid imines,^17^ and Co^II^, Ni^II^, Cu^II,^ and Zn^II^ centers by bipyridyl-appended oxazole cyclic peptides.^18^ There are also only two examples of cage-type complexes with M-SCs induced by amino acid derivatives: a heteronuclear Hg^II^Co^III^ complex containing l-cysteine^19^ and a spectacular large dodecanuclear complex with chirality on binuclear La^III^ clusters induced by amino acid-based ligands with *C*_3_ symmetry.^20^

In this paper, we show the design and synthesis of ligands with *C*_3_ symmetry, containing amino acids and hydrazone-based binding sites. We demonstrate that these ligands effectively induce M-SC chirality on octahedral Ga^III^ centers and form chiral cages, with C-SCs and M-SCs constituting the skeleton of the cage. As a result of such a well-defined geometry, effective self-sorting is also observed, so homochiral cages are formed from the mixtures of racemic ligands.

## Experimental Section

For further experimental details, crystallographic and computational data, see the SI.

### Synthetic Procedures and Analytical Data

#### *S*-**5a**

1,3,5-Benzenetricarboxylic acid **3** (1.33 mmol, 0.28 g) was dissolved in DMF (50 mL) and cooled to 0 °C. 1-Hydroxybenzotriazole hydrate (4.0 mmol, 0.61 g), *S*-phenylalanine methyl ester hydrochloride **4a** (4.0 mmol, 0.86 g), triethylamine (8.39 mmol, 1.17 mL), and EDCI (1-ethyl-3-(3-dimethylaminopropyl)carbodiimide hydrochloride, 4.39 mmol, 0.84 g) were added, and the mixture was stirred overnight at room temperature. The solvent was evaporated, and water was added to the yellow oil. The white precipitate was collected and washed with distilled water and saturated aqueous NaHCO_3_. The white powder was dried under reduced pressure. Yield: 0.89 g, 95%. ^1^H NMR (500 MHz, dimethyl sulfoxide-*d*_6_, 298 K): δ = 9.19 (d), *J* = 7.7 Hz, 3H), 8.37 (s, 3H), 7.32–7.24 (m, 12H), 7.23–7.19 (m, 3H), 4.74–4.66 (m, 3H), 3.64 (s, 9H), 3.18 (dd, *J* = 13.8, 5.4 Hz, 3H), 3.12 (dd, *J* = 13.8, 9.8 Hz, 3H). ^13^C NMR (125 MHz, dimethyl sulfoxide-*d*_6_, 298 K): δ = 171.96, 165.55, 137.57, 134.17, 129.24, 129.00, 128.27, 126.51, 54.44, 51.99, 36.16. HRMS (ESI) *m/z* calcd for C_39_H_39_N_3_O_9_Na: 716.2584 [M + Na]^+^, found: 716.2557.

#### *S*-**6a**

*S*-**5a** (1.0 mmol, 0.69 g) was dissolved in methanol (25 mL). Hydrazine hydrate (30 mmol, 1.46 mL) was added, and the mixture was heated at 70 °C in a sealed tube overnight with stirring. After cooling, the white precipitate was collected, washed with methanol, and dried under reduced pressure. Yield: 0.59 g, 86%. ^1^H NMR (500 MHz, dimethyl sulfoxide-*d*_6_, 298 K): δ = 9.35 (s, 3H), 8.77 ((d), *J* = 8.5 Hz, 3H), 8.25 (s, 3H), 7.35–7.12 (m, 15H), 4.76–4.69 (m, 3H), 4.27 (br s, 6H), 3.09–2.95 (m, 6H). ^13^C NMR (125 MHz, dimethyl sulfoxide-*d*_6_, 298 K): δ = 170.30, 165.41, 138.06, 134.32, 129.12, 128.14, 126.30, 53.62, 37.66. HRMS (ESI) *m/z* calcd for C_36_H_39_N_9_O_6_Na: 716.2921 [M + Na]^+^, found: 716.2912.

#### *S*-**1a**

*S*-**6a** (0.216 mmol, 0.150 g) was dissolved in methanol (10 mL). Salicyl aldehyde **7** (3.24 mmol, 0.34 mL) was added, and the mixture was heated at 70 °C in a sealed tube overnight with stirring. After cooling, the white precipitate was collected, washed with methanol, and dried under reduced pressure. Yield: 0.19 g, 88%. [α]_D_^22^ = 173.2 (c) = 1 in DMSO). The product was obtained as a mixture of two diastereoisomers in a 2:1 ratio. Main diastereoisomer ^1^H NMR (600 MHz, dimethyl sulfoxide-*d*_6_, 298 K): δ = 11.93 (s, 3H, *NH^1^*), 11.07–11.04 (m, 3H, *OH*), 9.07 (t, *J* = 7.7 Hz, 3H, *NH^2^*), 8.44 (s, 3H, *e*), 8.36 (s, 3H, (b)), 7.55–7.52 (m, 3H, *i*), 7.39–7.35 (m, 6H, *Ph*), 7.31–7.24 (m, 9H, *Ph*), 7.21–7.15 (m, 3H, *j*), 6.94–6.88 (m, 6H, *k* + *h*), 4.85–4.77 (m, 3H, α), 3.20–3.04 (m, 6H, β). ^13^C NMR (150 MHz, dimethyl sulfoxide-*d*_6_, 298 K): δ = 167.5 (d), 165.8 (c), 157.3 (*g*), 147.6 (*e*), 137.8 (*Ph*), 134.2 (*a*), 131.4 (*j*), 129.3 (b), 129.2 (*Ph*), 129.0 (*i*), 128.2 (*Ph*), 126.45 (*Ph*), 119.3 (*k*), 118.6 (*f*), 116.3 (*h*), 54.3 (α), 37.1 (β). Minor diastereoisomer ^1^H NMR (600 MHz, dimethyl sulfoxide-*d*_6_, 298 K): δ = 11.47 (s, 3H, *NH^1^*), 10.07 (s, 3H, *OH*), 8.92 (t, *J* = 8.7 Hz, 3H, *NH^2^*), 8.37 (s, 3H, *e*), 8.34 (s, 3H, (b)), 7.75–7.72 (m, 3H, *i*), 7.39–7.35 (m, 6H, *Ph*), 7.31–7.24 (m, 9H, *Ph*), 7.21–7.15 (m, 3H, *j*), 6.94–6.88 (m, 6H, *k* + *h*), 5.63–5.57 (m, 3H, α), 3.20–3.04 (m, 6H, β). ^13^C NMR (150 MHz, dimethyl sulfoxide-*d*_6_, 298 K): δ = 172.2 (d), 165.6 (c), 156.4 (*g*), 141.3 (*e*), 138.2 (*Ph*), 134.5 (*a*), 131.2 (*j*), 129.4 (b), 129.2 (*Ph*), 128.2 (*Ph*), 126.38 (*i*), 126.1 (*Ph*), 120.2 (*f*), 119.5 (*k*), 116.2 (*h*), 52.0 (α), 36.3 (β). HRMS (ESI) *m/z* calcd for C_57_H_50_N_9_O_9_: 1004.3731 [M-H]^−^, found: 1004.3692. IR (KBr, cm^–1^): 3640, 3215, 3058, 3029, 2933, 1682, 1641, 1625, 1559, 1531, 1489, 1454, 1362, 1324, 1275, 1237, 1153, 1107, 1078, 1033, 965, 938, 880, 856, 748, 698, 658, 610, 570, 517, 479. Analysis calcd for C_57_H_51_N_9_O_9_·1.5H_2_O: C 66.27, H 5.27, N 12.20, found: C 66.04, H 5.25, N 12.21.

#### *S*-**9a**

*S*-**1a** (0.06 mmol, 60.4 mg, 2 eq.), Ga(NO_3_)_3_·H_2_O (0.09 mmol, 24.6 mg, 3 eq.) and NaOH (0.36 mmol, 14.4 mg, 12 eq.) were dissolved in methanol (2 mL) and heated at 70 °C in a sealed tube overnight. After cooling, the solvent was evaporated, and the yellow solid was washed with water and dried under reduced pressure. Yield: 90%. ^1^H NMR (600 MHz, methanol-*d*_4_, 298 K): δ = 8.54 (s, 6H, *e*), 7.75 (s, 6H, (b)), 7.34–7.30 (m, 6H, *k*), 7.29–7.25 (m, 6H, *i*), 7.14–7.02 (m, 30H, *Ph*), 6.91–6.87 (m, 6H, *h*), 6.76–6.70 (m, 6H, *j*), 4.86 (dd, *J* = 6.2, 8.1 Hz, 6H, α), 3.11–3.04 (dd, *J* = 8.3, 13.4 Hz, 6H, β), 2.85–2.78 (dd, *J* = 6.2, 13.4 Hz, 6H, β). ^13^C NMR (150 MHz, methanol-*d*_4_, 298 K): δ = 173.7 (d), 167.3 (*g*), 166.8 (c), 158.7 (*e*), 138.4 (*Ph*), 135.4 (*a*), 134.8 (*k*), 134.7 (*i*), 130.7 (*Ph*), 129.1 (*Ph*), 129.0 (b), 127.4 (*Ph*), 122.4 (*h*), 119.0 (*f*), 117.0 (*j*), 55.7 (α), 39.9 (β). HRMS (ESI) *m/z* calcd for C_114_H_90_N_18_O_18_Ga_3_: 735.1482 [M]^3–^, found 735.1474. IR (KBr, cm^–1^): 3399, 3060, 3026, 2926, 1660, 1622, 1601, 1538, 1470, 1446, 1402, 1334, 1289, 1199, 1150, 1126, 1092, 1031, 969, 902, 854, 795, 756, 700, 585, 506. Analysis calcd for C_114_H_90_N_18_O_18_Ga_3_Na_3_·7H_2_O: C 56.95, H 4.36, N 10.49, found: C 56.98, H 4.35, N 10.73.

#### *S*-**5b**

1,3,5-Benzenetricarboxylic acid **3** (1.33 mmol, 0.28 g) was dissolved in DMF (50 mL) and cooled to 0 °C. HOBt hydrate (4.0 mmol, 0.61 g), *S*-alanine methyl ester hydrochloride **4b** (4.0 mmol, 0.56 g), triethylamine (8.39 mmol, 1.17 mL), and EDCI (4.39 mmol, 0.84 g) were added, and the mixture was stirred overnight at room temperature. The solvent was evaporated, and water was added to the yellow oil. The white precipitate was collected and washed with distilled water and saturated aqueous NaHCO_3_. The white powder was dried under reduced pressure. Yield: 89%. ^1^H NMR (500 MHz, dimethyl sulfoxide-*d*_6_, 298 K): δ = 9.13 (d), *J* = 6.8 Hz, 3H), 8.50 (s, 3H), 4.54–4.50 (m, 3H), 3.66 (s, 9H), 1.43 (d), *J* = 7.3 Hz, 9H). ^13^C NMR (125 MHz, dimethyl sulfoxide-*d*_6_, 298 K): δ = 172.97, 165.46, 134.22, 129.34, 51.93, 48.47, 16.68. HRMS (ESI) *m/z* calcd for C_21_H_27_N_3_O_9_Na: 488.1645 [M + Na]^+^, found 488.1651.

#### *S*-**6b**

*S*-**5b** (1.0 mmol, 0.47 g) was dissolved in methanol (25 mL). Hydrazine hydrate (30 mmol, 1.46 mL) was added, and the mixture was heated at 70 °C in a sealed tube overnight with stirring. After cooling, the white precipitate was collected, washed with methanol, and dried under reduced pressure. Yield: 94%. ^1^H NMR (500 MHz, dimethyl sulfoxide-*d*_6_, 298 K): δ = 9.22 (s, 3H), 8.68 (d), *J* = 7.6 Hz, 3H), 8.44 (s, 3H), 4.54–4.50 (m, 3H), 4.26 (s, 6H), 1.33 (d), *J* = 10.6 Hz, 9H). ^13^C NMR (125 MHz, dimethyl sulfoxide-*d*_6_, 298 K): δ = 171.46, 165.28, 134.27, 129.29, 47.81, 18.26. HRMS (ESI) *m/z* calcd for C_18_H_26_N_9_O_6_: 464.2006 [M-H]^−^, found 464.2010.

#### *S*-**1b**

*S*-**6b** (0.216 mmol, 0.100 g) was dissolved in methanol (10 mL). Salicyl aldehyde **7** (3.24 mmol, 0.34 mL) was added, and the mixture was heated at 70 °C in a sealed tube overnight with stirring. After cooling, the white precipitate was collected, washed with methanol, and dried under reduced pressure. Yield: 80%. [α]_D_^22^ = 174.5 ((c) = 1 in DMSO). The product was obtained as a mixture of two diastereoisomers in 2.6:1 ratio. Main diastereoisomer ^1^H NMR (500 MHz, dimethyl sulfoxide-*d*_6_, 298 K): δ = 11.83 (s, 3H, *NH^1^*), 11.14–11.10 (m, 3H, *OH*), 8.98–8.95 (m, 3H, *NH^2^*), 8.56 (s, 3H, *e*), 8.46 (s, 3H, (b)), 7.54–7.49 (m, 3H, *i*), 7.32–7.20 (m, 3H, *j*), 6.94–6.85 (m, 6H, *k* + *h*), 4.62–4.55 (m, 3H, α), 1.46 ((d), *J* = 7.0 Hz, 9H, β). ^13^C NMR (125 MHz, dimethyl sulfoxide-*d*_6_, 298 K): δ = 168.7 (d), 165.7 (c), 157.3 (*g*), 147.5 (*e*), 134.2 (*a*), 131.3 (*j*), 129.38 (*i*), 129.45 (b), 119.3 (*k*), 118.6 (*f*), 116.4 (*h*), 48.5 (α), 17.6 (β). Minor diastereoisomer ^1^H NMR (500 MHz, dimethyl sulfoxide-*d*_6_, 298 K): δ = 11.38 (s, 3H, *NH^1^*), 10.07 (s, 3H, *OH*), 8.85–8.82 (m, 3H, *NH^2^*), 8.54 (s, 3H, *e*), 8.33 (s, 3H, (b)), 7.71–7.67 (m, 3H, *i*), 7.32–7.20 (m, 3H, *j*), 6.94–6.85 (m, 6H, *k* + *h*), 5.37–5.30 (m, 3H, α), 1.46 ((d), *J* = 6.8 Hz, 9H, β). ^13^C NMR (125 MHz, dimethyl sulfoxide-*d*_6_, 298 K): δ = 173.2 (d), 165.4 (c), 156.4 (*g*), 141.1 (*e*), 134.5 (*a*), 131.1 (*j*), 129.6 (b), 126.5 (*i*), 120.2 (*f*), 119.5 (*k*), 116.2 (*h*), 46.3 (α), 16.7 (β). HRMS (ESI) *m/z* calcd for C_39_H_38_N_9_O_9_: 776.2792 [M-H]^−^, found 776.2777. IR (KBr, cm^–1^): 3220, 3054, 1657, 1622, 1531, 1489, 1453, 1388, 1361, 1272, 1222, 1154, 1101, 1035, 965, 883, 856, 755, 688, 658, 545, 477. Analysis calcd for C_39_H_39_N_9_O_9_·H_2_O: C 58.86, H 5.19, N 15.84, found: C 58.63, H 5.13, N 15.72.

#### *S*-**9b**

*S*-**1b** (0.06 mmol, 46.7 mg, 2 eq.), Ga(NO_3_)_3_·H_2_O (0.09 mmol, 24.6 mg, 3 eq.), and NaOH (0.36 mmol, 14.4 mg, 12 eq.) were dissolved in methanol (2 mL) and heated at 70 °C in a sealed tube overnight. After cooling, the solvent was evaporated, and the yellow solid was washed with a water/acetone 1:1 mixture and dried under reduced pressure. Yield: 80%. ^1^H NMR (600 MHz, methanol-*d*_4_, 298 K): δ = 8.63 (s, 6H, *e*), 7.82 (s, 6H, (b)), 7.34–7.31 (m, 6H, *k*), 7.23–7.18 (m, 6H, *i*), 6.75–6.67 (m, 12H, *h* + *j*), 4.75 (q, *J* = 7.1 Hz, 6H, α), 1.31 ((d), *J* = 7.1 Hz, 12H, β). ^13^C NMR (150 MHz, methanol-*d*_4_, 298 K): δ = 175.3 (d), 167.7 (c), 167.1 (*g*), 158.7 (*e*), 135.6 (*a*), 134.7 (*k*), 134.6 (*i*), 129.0 (b), 122.1 (*h*), 118.8 (*f*), 117.1 (*j*), 49.9 (α), 18.9 (β). HRMS (ESI) *m/z* calcd for C_78_H_66_N_18_O_18_Ga_3_: 583.0857 [M]^3–^, found 583.0848. IR (KBr, cm^–1^): 3388, 2427, 1789, 1658, 1624, 1601, 1534, 1472, 1446, 1384, 1291, 1200, 1152, 1126, 1093, 1036, 984, 902, 836, 799, 760, 663, 586, 518, 482, 419.

#### *S*-**2a**

*S*-**12a** (1 mmol, 0.283 g) was dissolved in methanol (5 mL), and 2 equivalents of salicyl aldehyde **7** were added (2 mmol, 0.19 mL). The reaction mixture was heated for 24 h at 70 °C in a sealed tube and then evaporated. The solid was washed with diethyl ether and dried under reduced pressure. Yield: 0.295 g, 76%. [α]_D_^25^ = 175.1 ((c) = 1 in DMSO). The product was obtained as a mixture of two diastereoisomers in 2.2:1 ratio. Main diastereoisomer ^1^H NMR (400 MHz, dimethyl sulfoxide-*d*_6_, 303 K): δ = 11.88 (s, 1H, *NH^1^*), 11.07 (s, 1H, *OH*), 8.81 ((d), *J* = 8.0 Hz, 1H, *NH^2^*), 8.46 (s, 1H, *g*), 7.86–7.81 (m, 2H, (c)), 7.56–7.24 (m, 9H, *Ph* + *j* + (d) + (b)), 7.22–7.15 (m, 1H, *k*), 6.95–6.88 (m, 2H, *i* + *l*), 4.81–4.73 (m, 1H, α), 3.20–3.02 ppm (m, 2H, β). ^13^C NMR (100 MHz, dimethyl sulfoxide-*d*_6_, 303 K): δ = 167.8 (*f*), 166.5 (*e*), 157.3 (*h*), 147.5 (*g*), 138.0 (*a*), 133.8 (*Ph*), 131.3 ((d) + *k*), 129.3 (*j*), 129.1 (*Ph*), 128.15 ((b) + *Ph*), 127.5 (c), 126.35 (*Ph*), 119.3 (*l*), 118.6 (*m*), 116.3 (*i*), 54.2 (α), 36.9 (β). Minor diastereoisomer ^1^H NMR (400 MHz, dimethyl sulfoxide-*d*_6_, 303 K): δ = 11.41 (s, 1H, *NH^1^*), 10.07 (s, 1H, *OH*), 8.67 ((d), *J* = 8.3 Hz, 1H, *NH^2^*), 8.36 (s, 1H, *g*), 7.86–7.81 (m, 2H, (c)), 7.76–7.72 (m, 1H, *j*), 7.56–7.24 (m, 8H, *Ph* + (d) + (b)), 7.22–7.15 (m, 1H, *k*), 6.95–6.88 (m, 2H, *i* + *l*), 5.58–5.51 (m, 1H, α), 3.20–3.02 ppm (m, 2H, β). ^13^C NMR (100 MHz, dimethyl sulfoxide-*d*_6_, 303 K): δ = 172.4 (*f*), 166.3 (*e*), 156.4 (*h*), 141.2 (*g*), 138.4 (*a*), 134.0 (*Ph*), 131.2 (d), 131.1 (*k*), 129.0 (*Ph*), 128.12 ((b) + *Ph*), 127.4 (c), 126.31 (*j*), 126.1 (*Ph*), 120.2 (*m*), 119.4 (*l*), 116.2 (*i*), 52.0 (α), 36.1 (β). HRMS (EI) *m/z* calcd for C_23_H_21_N_3_O_3_: 387.1583 [M]^+^, found: 387.1591. IR (KBr, cm^–1^): 3265, 3059, 3029, 2973, 2925, 2868, 1675, 1641, 1577, 1538, 1487, 1439, 1415, 1351, 1334, 1270, 1240, 1214, 1199, 1153, 1083, 1033, 958, 929, 876, 848, 792, 753, 698, 656, 597, 567, 548, 516, 497, 473, 441. Analysis calcd for C_23_H_21_N_3_O_3_: C 71.30, H 5.46, N 10.85, found: C 71.18, H 5.42, N 10.80.

#### *S*-**10a**

*S*-**2a** (0.02 mmol, 7.7 mg, 2 eq.), Ga(NO_3_)_3_·H_2_O (0.01 mmol, 2.7 mg, 1 eq.) and NaOH (0.04 mmol, 1.6 mg, 4 eq.) were dissolved in methanol (0.7 mL) and heated at 70 °C in a sealed tube overnight. The complex was obtained as a mixture of diastereoisomers. ^1^H NMR (400 MHz, methanol-*d*_4_, 303 K): δ = 8.53 (s), 8.526 (s), 8.523 (s), 8.51 (s), 8.34 (s), 7.77–7.70 (m), 7.66–7.63 (m), 7.60–7.04 (m), 6.81–6.63 (m), 5.00–4.87 (m), 3.25–3.12 (m), 3.10–3.00 (m), 2.95–2.87 (m). HRMS (APCI) *m/z* calcd for C_46_H_38_N_6_O_6_Ga: 839.2109 [M]^−^, found 839.2104. IR (KBr, cm^–1^): 3422, 3060, 3027, 2427, 1623, 1578, 1530, 1485, 1471, 1446, 1384, 1288, 1199, 1151, 1125, 1077, 1031, 969, 902, 849, 795, 756, 701, 660, 585, 510, 417. Analysis calcd for C_46_H_38_N_6_O_6_GaNa·2NaNO_3_·4H_2_O: C 49.97, H 4.19, N 10.14, found: C 50.12, H 3.97, N 10.06.

#### *S*-**2b**

*S*-**12b** (1 mmol, 0.207 g) was dissolved in methanol (5 mL), and 2 equivalents of salicyl aldehyde **7** were added (2 mmol, 0.19 mL). The reaction mixture was heated 24 h at 70 °C in a sealed tube and then evaporated. The solid was washed with diethyl ether and dried under reduced pressure. Yield: 60%. [α]_D_^25^ = 176.8 ((c) = 1 in DMSO). The product was obtained as a mixture of two diastereoisomers in a 2.5:1 ratio. Major diastereoisomer ^1^H NMR (600 MHz, dimethyl sulfoxide-*d*_6_, 298 K): δ = 11.78 (s, 1H, *NH^1^*), 11.13 (s, 1H, *OH*), 8.71 ((d), *J* = 7.0 Hz, 1H, *NH^2^*), 8.46 (s, 1H, *g*), 7.94–7.91 (m, 2H, (c)), 7.57–7.52 (m, 1H, (d)), 7.52–7.50 (m, 1H, *j*), 7.50–7.45 (m, 2H, (b)), 7.30–7.26 (m, 1H, *k*), 6.93–6.89 (m, 2H, *i* + *l*), 4.53 (dq, *J*_1_ = 7.0 Hz, *J*_2_ = 7.1 Hz, 1H, α), 1.42 ppm ((d), *J* = 7.1 Hz, 3H, β). ^13^C NMR (150 MHz, dimethyl sulfoxide-*d*_6_, 298 K): δ = 168.9 (*f*), 166.3 (*e*), 157.3 (*h*), 147.4 (*g*), 133.8 (*a*), 131.4 (d), 131.32 (*k*), 129.4 (*j*), 128.2 (b), 127.6 (c), 119.3 (*l*), 118.7 (*m*), 116.4 (*i*), 48.3 (α), 17.5 ppm (β). Minor diastereoisomer ^1^H NMR (600 MHz, dimethyl sulfoxide-*d*_6_, 298 K): δ = 11.34 (s, 1H, *NH^1^*), 10.08 (s, 1H, *OH*), 8.69 ((d), *J* = 7.4 Hz, 1H, *NH^2^*), 8.32 (s, 1H, *g*), 7.92–7.89 (m, 2H, (c)), 7.70–7.68 (m, 1H, *j*), 7.57–7.52 (m, 1H, (d)), 7.50–7.45 (m, 2H, (b)), 7.26–7.22 (m, 1H, *k*), 6.89–6.85 (m, 2H, *i* + *l*), 5.27 (dq, *J*_1_ = 7.4 Hz, *J*_2_ = 7.2 Hz, 1H, α), 1.43 ppm ((d), *J* = 7.2 Hz, 3H, β). ^13^C NMR (150 MHz, dimethyl sulfoxide-*d*_6_, 298 K): δ = 173.5 (*f*), 166.0 (*e*), 156.4 (*h*), 140.9 (*g*), 134.1 (*a*), 131.29 (d), 131.1 (*k*), 128.2 (b), 127.5 (c), 126.3 (*j*), 120.2 (*m*), 119.5 (*l*), 116.2 (*i*), 46.1 (α), 16.6 ppm (β). HRMS (EI) *m/z* calcd for C_17_H_17_N_3_O_3_: 311.1270 [M]^+^, found: 311.1273. IR (KBr, cm^–1^): 3280, 3191, 3061, 2979, 2868, 1675, 1636, 1577, 1531, 1487, 1448, 1408, 1370, 1342, 1297, 1274, 1217, 1200, 1153, 1123, 1098, 1033, 957, 930, 907, 892, 800, 754, 715, 693, 649, 583, 548, 474, 444, 428. Analysis calcd for C_17_H_17_N_3_O_3_: C 65.58, H 5.50, N 13.50, found: C 65.41, H 5.54, N 13.44.

#### *S*-**10b**

*S*-**2b** (0.02 mmol, 6.2 mg, 2 eq.), Ga(NO_3_)_3_·H_2_O (0.01 mmol, 2.7 mg, 1 eq.) and NaOH (0.04 mmol, 1.6 mg, 4 eq.) were dissolved in methanol (0.7 mL) and heated at 70 °C in a sealed tube overnight. The complex was obtained as a mixture of diastereoisomers in a 45:55 ratio. Major diastereoisomer ^1^H NMR (600 MHz, methanol-*d*_4_, 298 K): δ = 8.55 (s, 2H, *g*), 7.69–7.66 (m, 4H, (b)), 7.49–7.45 (m, 2H, (d)), 7.40–7.35 (m, 4H, (c)), 7.28–7.25 (m, 2H, *l*), 7.15–7.11 (m, 2H, *j*), 6.71–6.64 (m, 4H, *i* + *k*), 4.77–4.71 (m, 2H, α), 1.41 ((d), *J* = 7.0 Hz, 6H, β). ^13^C NMR (150 MHz, methanol-*d*_4_, 298 K): δ = 175.14 (*f*), 169.2 (*e*), 167.1 (*h*), 158.5 (*g*), 135.7 (*a*), 134.57 (*l*), 134.45 (*j*), 132.5 (d), 129.46 (c), 128.29 (b), 122.2 (*i*), 119.2 (*m*), 117.1 (*k*), 49.9(α), 19.5 (β). Minor diastereoisomer ^1^H NMR (600 MHz, methanol-*d*_4_, 298 K): δ = 8.56 (s, 2H, *g*), 7.76–7.73 (m, 4H, (b)), 7.49–7.45 (m, 2H, (d)), 7.40–7.35 (m, 4H, (c)), 7.28–7.25 (m, 2H, *l*), 7.20–7.16 (m, 2H, *j*), 6.71–6.64 (m, 4H, *i* + *k*), 4.77–4.71 (m, 2H, α), 1.35 ((d), *J* = 7.0 Hz, 6H, β). ^13^C NMR (150 MHz, methanol-*d*_4_, 298 K): δ = 175.11 (*f*), 169.1 (*e*), 167.0 (*h*), 158.6 (*g*), 135.6 (*a*), 134.63 (*l*), 134.41 (*j*), 132.6 (d), 129.45 (c), 128.31 (b), 122.1 (*i*), 119.1 (*m*), 117.0 (*k*), 49.7 (α), 19.6 (β). HRMS (APCI) *m/z* calcd for C_34_H_30_N_6_O_6_Ga: 687.1483 [M]^−^, found 687.1480. IR (KBr, cm^–1^): 3410, 3060, 3028, 2981, 2935, 2428, 1789, 1624, 1602, 1578, 1529, 1486, 1471, 1446, 1366, 1292, 1200, 1152, 1123, 1075, 1034, 970, 901, 836, 797, 758, 714, 660, 585, 481. Analysis calcd for C_34_H_30_N_6_O_6_GaNa·2NaNO_3_·3H_2_O: C 43.66, H 3.88, N 11.98, found: C 43.66, H 3.67, N 11.88.

## Results

### Design and Synthesis

**Figure 1:**
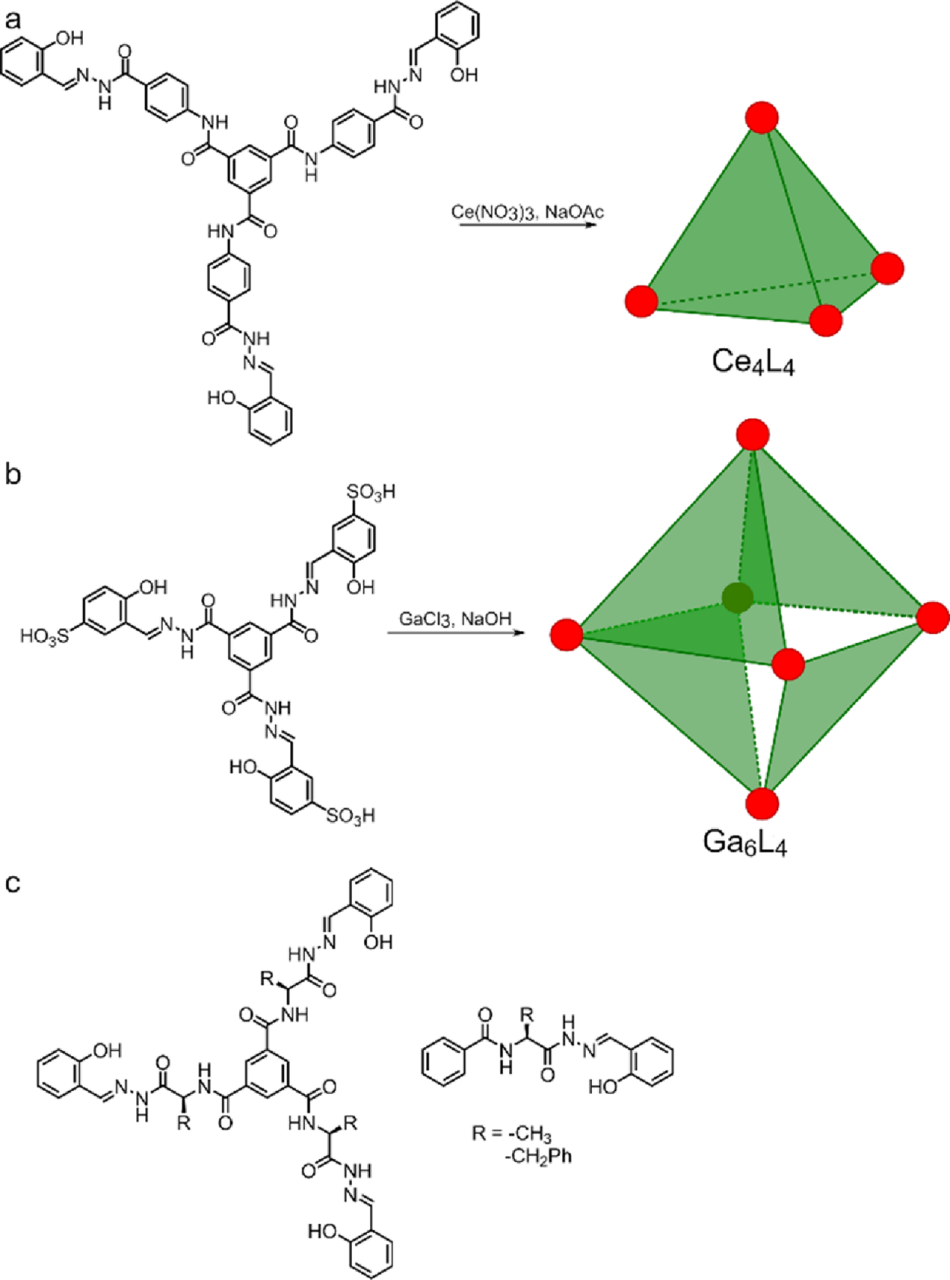
Previously reported complexes of salicyl-acylhydrazone ligands (a, b).^22,13^ Ligands designed in this work (c).

**Scheme 1:**
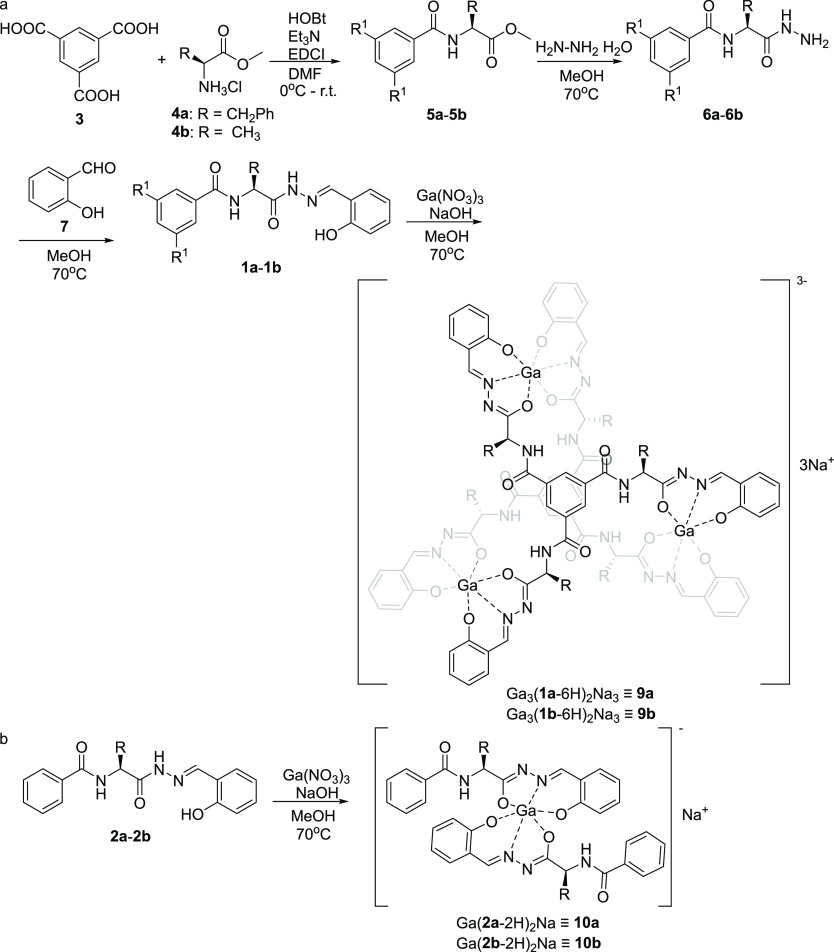
Synthesis of Ligands and Complexes: (a) Ligands **1a** and **1b** and Complexes **9a** and 9b; (b) Complexes **10a** and **10b**

We have designed new tripodal ligands **1a** and **1b** (Figure 1c and Scheme 1a) that contain chiral amino acid residues and salicyl-acylhydrazone units which, upon di-deprotonation, constitute tridentate coordination sites. Inspiration was taken from previously reported rigid and achiral salicyl-acylhydrazones that are known to form tetrahedral M_4_L_4_ (M = Ce; Figure 1a)^21^ or octahedral M_6_L_4_ (M = Ga^III^, Ni^III^; Figure 1b)^13,22^ cages. Newly designed **1a** and **1b** ligands, in addition to being chiral, are non-planar and possess a considerably higher conformational flexibility than the previously known ligands, enabling higher structural diversity of the resulting cages in terms of symmetry and possible stoichiometry. Additionally, M-SCs (Λ or Δ), which are present next to C-SCs, can be induced during the complexation. Considering that the configuration of all M-SCs present in a single cage does not have to be identical, it is non-trivial to predict the possible stoichiometry and geometry of the cages based on **1a** and **1b**.

The synthesis of ligands **1a** and **1b** starts from the coupling of benzene-1,3,5-tricarboxylic acid **3** with amino acid methyl esters **4a** and **4b** using standard coupling reagents to obtain triesters **5a** and **5b** in 89–95% yield. Triesters were next subjected to the reaction with hydrazine hydrate in methanol to obtain trihydrazides **6a** and **6b**, which typically precipitate from the reaction mixtures and are isolated in analytically pure forms by filtration (yields: 86–94%). The trihydrazides were reacted with salicylaldehyde **7** to give final hydrazones **1a** and **1b** in an 80–88% yield. Ligands **2a** and **2b**, which are used as reference compounds, were also synthesized by analogous procedures, starting from benzoic acid **8** (Scheme S1). In the ^1^H NMR spectra of ligands **1a** and **1b** in DMSO-*d*_6_, there are two sets of signals: in 2:1 ratio for **1a** and 2.6:1 ratio for **1b** (Figures S1 and S20). Two sets of signals are also observed in the NMR in DMSO-*d*_6_ of hydrazones **2a** and **2b**, (in 2.2:1 and 2.5:1 ratio, respectively; Figures S29 and S33). The 2D NOESY NMR spectra indicate that there is a chemical exchange between the two sets of signals (observed for α and imine protons, Figure S36). Therefore, it can be concluded that the signals derive from two conformers of hydrazones present in the solution. The exact structure of the conformers remains unknown because of the lack of relevant NOEs; however, it can be assumed that they originate from inhibited rotation around one or more partial double bonds present in the structure. Comparison of the differences in chemical shifts for the isomers (*C*O, N*H*, and C*H*α signals) with literature data^23^ suggests that the isomers are most likely *cis*- and *trans*-amides. This suggestion is further supported by the analysis of CCDC (The Cambridge Crystallographic Data Centre), which contains about 20 examples of *cis*-amides for salicyl-acylhydrazones, while *cis*-hydrazones are observed only in the case of metal coordination.

The synthesis of cages **9a** and **9b** involves the reaction between hydrazones **1a** and **1b** and Ga(NO_3_)_3_ in methanol in the presence of NaOH in ratio 2:3:12. The reference complexes **10a** and **10b** were obtained by an analogous procedure using **2a** and **2b**, Ga(NO_3_)_3,_ and NaOH in a ratio of 2:1:4. The complexes were isolated by evaporation of the solvent, washed with water, and analyzed by mass spectrometry, NMR, and circular dichroism.

### Structures of the Cages

**Figure 2:**
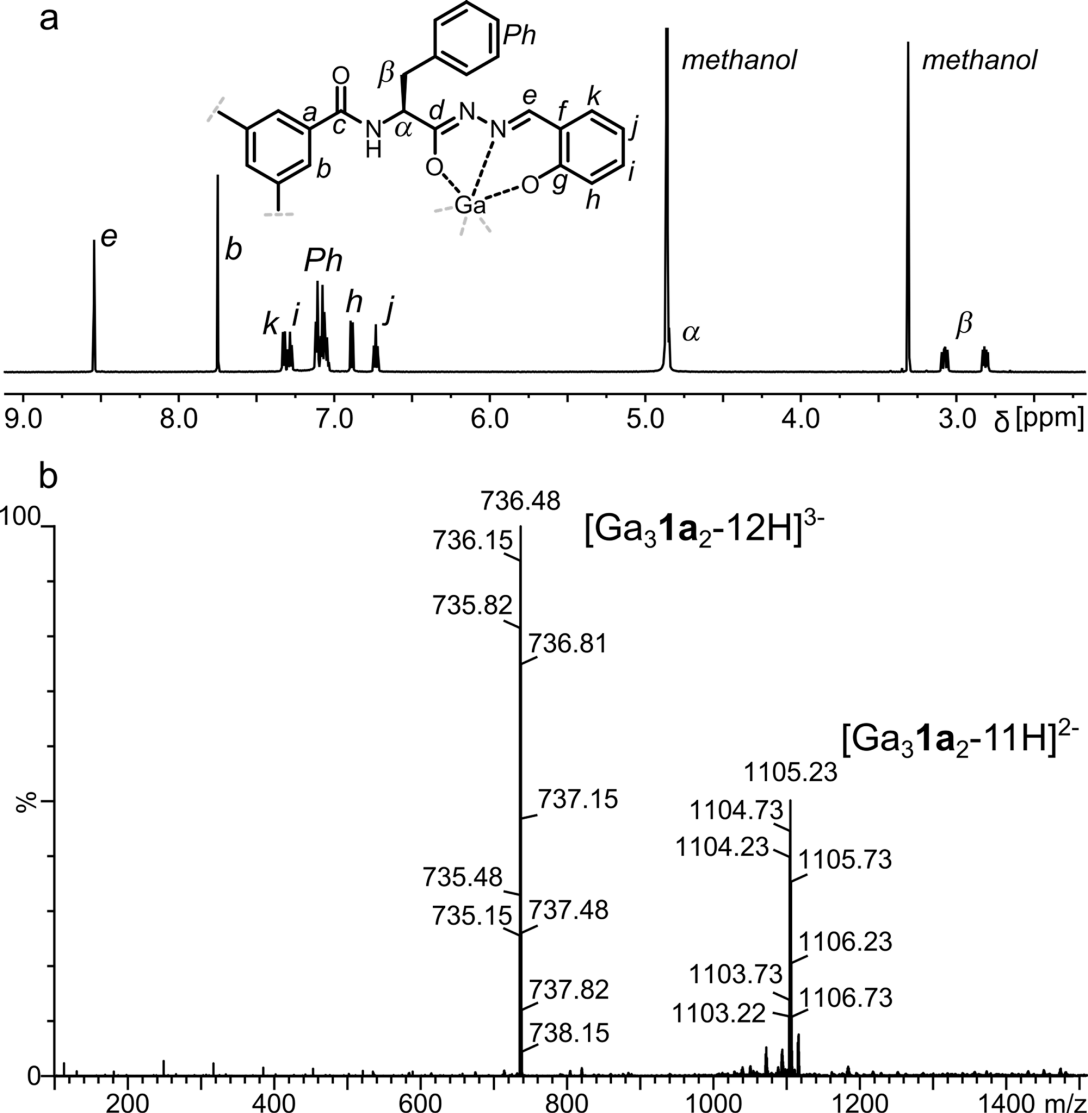
(a) ^1^H NMR spectrum of **9a** (methanol-*d*_4_, 298 K, 600 MHz); (b) ESI MS spectrum of **9a**.

The ESI MS spectrum of **9a** (Figure 2b and Figure S19) reveals peaks corresponding to [Ga_3_(*S*-**1a**-6H)_2_]^3–^ and [Ga_3_(*S*-**1a**-6H)_2_ + H]^2–^, indicating that **9a** is an M_3_L_2_ cage formed by double deprotonation at each arm of the ligand and subsequent coordination to Ga^3+^ with the final Na_3_[Ga_3_(*S*-**1a**-6H)_2_] composition. The ESI MS spectrum of **9b** shows the formation of a similar M_3_L_2_ cage upon coordination with Ga^3+^ (Figure S28). The M_3_L_2_ cages are smaller than previously reported cages M_6_L_4_ and M_4_L_4_ obtained using rigid, achiral ligands. The ^1^H and ^13^C NMR spectra of **9a** and **9b** in methanol-*d*_4_ exhibit single sets of signals, and the number of signals is reduced by *D*_3_ symmetry, indicating the formation of a single diastereoisomer in both cases (Figure 2a and Figures S7 and S22). This leads to the conclusion that all metal centers within the molecule have the same configuration, and this configuration was stereoselectively induced by amino acids. Quite surprisingly, **9a**, despite its charged character, is also soluble in THF-*d*_8_, and the ^1^H NMR spectrum of **9a** indicates that deprotonation occurs in the salicyl *OH* and *NH^1^* groups, whereas the *NH^2^* groups remain protonated (Figure S13). The deprotonation sites are also confirmed by ^13^C NMR spectra, and the *g* and *e* signals of cages **9a** and **9b** are significantly downfield shifted compared to the respective signals in the spectra of **1a** and **1b**.

**Figure 3:**
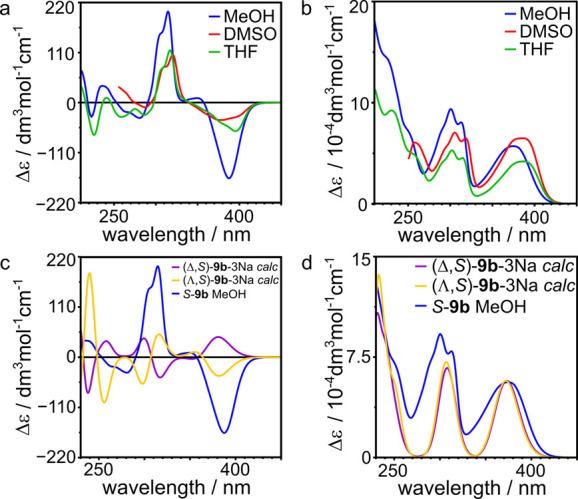
(a, b) Experimental ECD and UV spectra of *S***-9b** in various solvents. (c, d) Comparison of the experimental and calculated ECD and UV spectra of *S***-9b**.

To determine the configuration of the complexes, we recorded the ECD (electronic circular dichroism) and UV spectra and compared them with the theoretically calculated ones. The ECD and UV spectra of *S***-9b** in various solvents (methanol, THF, and DMSO) are similar and show strong CD effects (Figure 3a and Figure S53 for *S***-9a**). The UV bands for complexes are batochromically shifted in comparison to those of ligands, and the lowest energy band at 390 nm gives rise to a strong positive couplet-type band in the CD spectrum.

**Figure 4:**
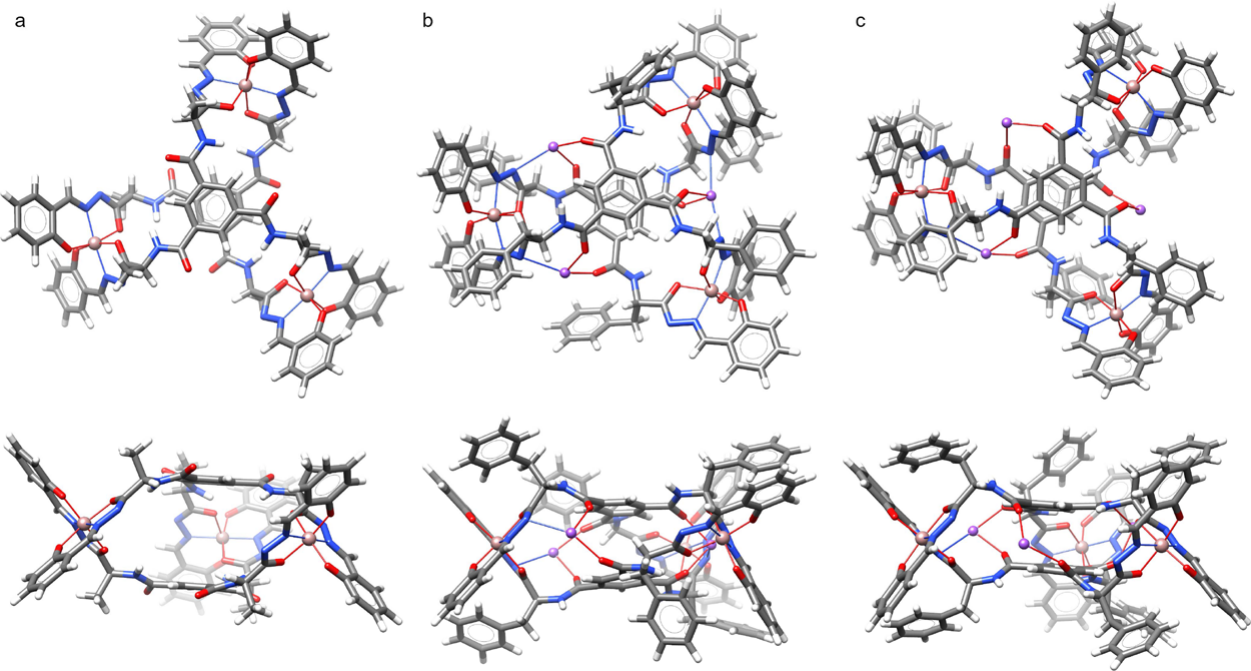
(a) Geometry-optimized structure of (Λ,*S*)-[**9b**-3Na]^3–^, top and side view. (b) Geometry-optimized structure of (Λ,*S*)-**9a**, top and side view. (c) Crystal structure of (Λ,*S*)-**9a**, top and side view.

Geometry optimization calculations^24^ for two diastereoisomers (Λ,*S*)-[**9b**-3Na]^3–^ and (Δ,*S*)-[**9b**-3Na]^3–^ were performed by DFT B3LYP/6-31g/cc-PVDZ, and for the optimized structures, the UV/Vis and ECD spectra were calculated (TD DFT wb97xd/6-31g/cc-PVDZ). Pre-optimization models were constructed based on the basis of the geometry of metal complexes with salicyl hydrazones derived from the CCDC database. The lowest energy structure has *D*_3_ symmetry and (Λ,*S*)-[**9b**-3Na]^3–^ configuration (Figure 4a). The two benzene-1,3,5-tricarbonyl cores are parallel to each other (the distance is 6.1 Å), and the amino acid arms of the ligands are twisted. The second diastereoisomer, (Δ,*S*)-[**9b**-3Na]^3–^ (Figure S66b), has a higher energy (by 17.8 kcal/mol in vacuo and 12.3 kcal/mol in methanol) and *C*_1_ symmetry with steric crowding between the side chains and non-parallel position of two benzene-1,3,5-tricarbonyl cores (distance 4.1–5.0 Å). Based on these calculations, we assume that M_3_L_2_ metal cages with (Λ,*S*) should be preferentially formed. This conclusion is further supported by calculations of the ECD spectra for the optimized structures of diastereoisomers. The theoretical ECD spectrum for (Λ,*S*)-[**9b**-3Na]^3–^ agrees with the experimental spectrum, while the ECD spectrum for (Δ,*S*)-[**9b**-3Na]^3–^ resembles its mirror image (Figure 3c). This pseudo-enatiomeric relationship is in agreement with the fact that the signs of all bands above 300 nm depend on the chirality of the metal centers, which are opposite for the diastereoisomers. The difference between the experimental and calculated spectra observed for the band at 300 nm may originate from small conformational differences because more than 20 orbitals from different parts of the molecule contribute to this band (Figures S67 and S68). However, this discrepancy does not alter the main conclusion concerning the chirality at the metal centers.

Crystals of **9a** suitable for X-ray were obtained by evaporation of the water/methanol mixture. The crystal structure (Figure 4c) confirmed that this complex has (Λ,*S*) configuration; the same as determined by calculations. The symmetry of each ligand is close to *C*_3_, but the whole complex is not *D*_3_-symmetrical, which is caused by a translational shift of the benzene-1,3,5-tricarbonyl cores with respect to each other along the ring plane. These two benzene-1,3,5-tricarbonyl cores are closer to each other in the solid state (distance 3.3–3.6 Å) than in the calculated model (distance 6.1 Å), and the carbonyl groups attached to the core ring are directed inside the cavity, not outside like in the calculated structure. These differences can originate from secondary interactions present in the solid state—coordination of oxygen atoms to sodium ions or packing effects that favor a more compact structure without the internal void. Indeed, when the structure (Λ,*S*)-[**9a**-3Na]^3–^, having molecular geometry derived from X-ray analysis, was subjected to geometry optimization, it converged to the open structure, identical to the one that was obtained initially by modeling. Further calculations that take into account interactions with Na^+^ cations, suggested by the X-ray structure, also indicate that (Λ,*S*)-**9a** (Figure 4b) has a lower energy (by 20.2 kcal/mol in methanol). In this case, the distance between two core rings is about 4.3 Å, so it is longer than in the crystal structure but shorter than in (Λ,*S*)-[**9a**-3Na]^3–^.

**Figure 5:**
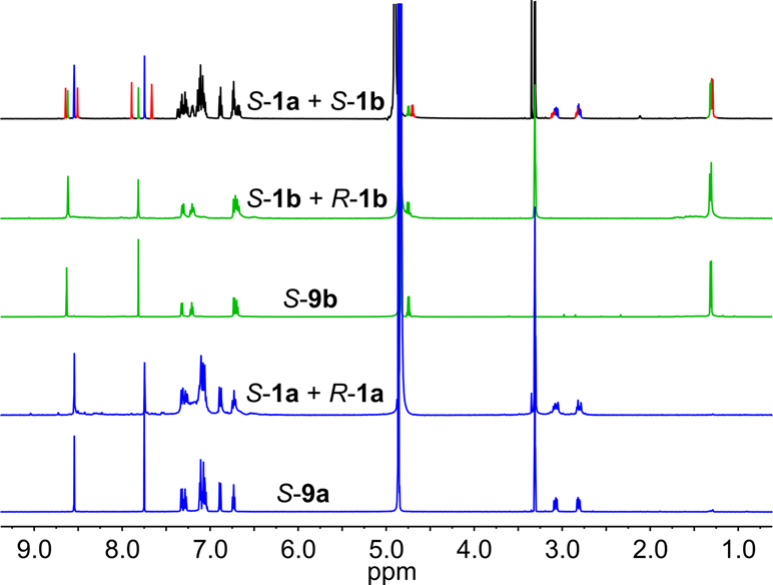
^1^H NMR spectra of complexes and self-sorting mixtures (blue, **9a**; green, **9b**; red, mixed complex).

The strong preference for one diastereoisomer of Ga_3_L_2_ (observed experimentally and predicted theoretically) and the dynamic character of the coordination bonds prompted us to examine the chiral self-sorting between ligands during the formation of cages. The NMR spectrum of the reaction mixture containing ligands *S*-**1a** and *R*-**1a** is almost identical to the NMR spectrum after the reaction with enantiomerically pure ligands, indicating very effective self-sorting based on chirality (Figure 5). The same results were obtained for the mixture of *S*-**1b** and *R*-**1b** ligands. However, in the NMR spectrum of a reaction mixture containing ligands of the same chirality, *S*-**1b** and *S*-**1a**, there are signals of homodimeric cages and also a new set of signals coming from the heterodimeric cage. The chiral sorting phenomenon is not common, and in the literature, there are only a few examples of coordination cages with the ability to chiral self-sorting.^11,25^

**Figure 6:**
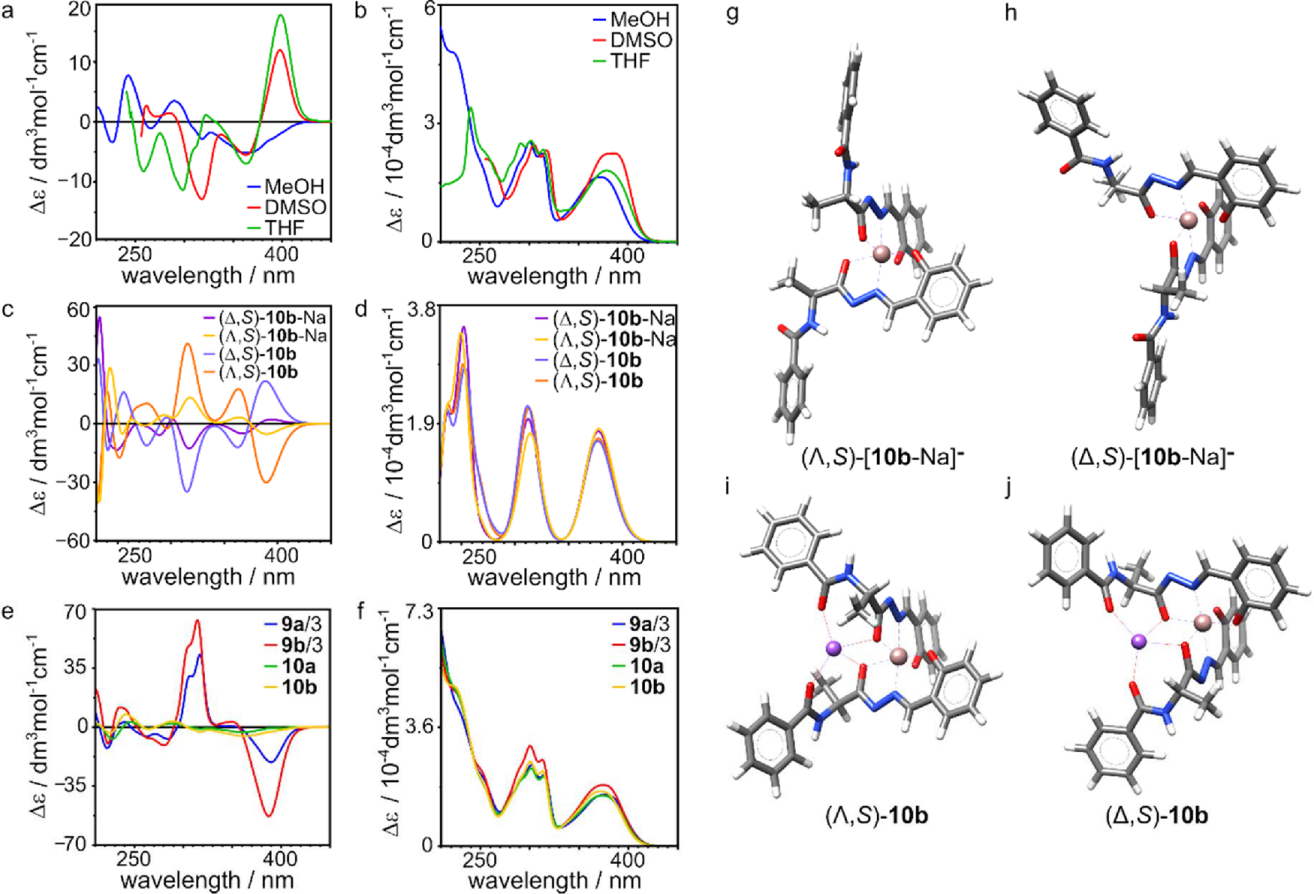
(a, b) Experimental ECD and UV spectra of *S*-**10b**. (c, d) Calculated ECD and UV spectra of *S*-**10b**. (e, f) Normalized ECD and UV spectra of all complexes. (g–j) Optimized models of *S*-**10b**.

The induction of chirality in the metal centers is a unique feature of the cage geometry because for the reference complex **10b**, two diastereoisomers (Λ,*S*)-**10b** or (Δ,*S*)-**10b** were formed. In the ^1^H NMR spectrum of **10b** in methanol, two sets of signals are observed (ratio 1:1.2; Figure S39), and the 2D NMR spectra show no NOE/ROEs between these sets (Figures S42 and S43). In DMSO, the intensity ratio between the two sets is 1:1.7 (Figure S46). These data indicate that for linear ligands, the amino acid C-SCs are not able to stereoselectively induce chirality in the metal center. Different ratios between diastereoisomers observed in different solvents suggest that chirality at the metal stereogenic center is dynamic under current conditions. Indeed, after mixing complexes **10a** and **10b** in methanol, new sets of signals coming from mixed complexes emerge in the ^1^H NMR spectrum, indicating the dynamic exchange of ligands (Figure S47). The intensity of CD bands for complexes **10b** and **10a** is low (Figure 6a and Figure S54), which is attributed to an overlap of the spectra of two diastereoisomers (Λ*,S*)-**10b** or (Δ*,S*)-**10b** having opposite configurations at the metal centers. Significant differences between ECD spectra in methanol and THF or DMSO reflect different diastereomeric ratios in these solvents. For further comparison of the relative intensity of the effects, the UV and ECD spectra for **9a**, **9b**, **10a**, and **10b** were normalized per single Ga^3+^ structural unit (the **9a** and **9b** spectra were divided by 3). The intensities of UV bands in complexes are almost identical (Figure 6f); however, the intensities of CD bands are considerably higher for cages than for the dimers (Figure 6e) in line with the above-presented interpretation.

The calculations of the energies and ECD spectra of diastereoisomers of **10b** were performed for two possible situations: with additional interactions with Na^+^ (**10b**) and neglecting these interactions ([**10b**-Na]^−^; Figure 6g–j). When additional interactions are neglected the energy difference between (Λ,*S*)-[**10b**-Na]^−^ and (Δ*,S*)-[**10b**-Na]^−^ is small (0.14–0.19 kcal/mol, in all solvents) and indicates the ratio of c. a. 1.5:1 in favor of [(Λ,*S*)-[**10b**-Na]^−^. However, when additional interactions with Na^+^ are taken into account, the calculated energy for the second diastereoisomer, (Δ,*S*)-**10b**, is lower than for (Λ,*S*)-**10b** by approximately 4 kcal/mol (in all solvents); therefore, (Δ,*S*)-**10b** should be the only observable diastereoisomer. Calculation of ECD spectra for all four structures shows that the spectra of respective diastereoisomers, e.g. (Δ,*S*)-**10b** vs (Λ,*S*)-**10b**, resemble the spectra of enantiomers, indicating that the signs of ECD effects are dominated by chirality at M-SCs. The presence of Na^+^ influences only the intensity of the ECD bands for a given isomer, but it does not change the signs of the bands.

Based on a comparison of the calculated and experimental ECD spectra, it can be concluded that in THF and DMSO, the dominant diastereoisomer is (Δ,*S*)-**10b**. This preference qualitatively agrees with the preference suggested by calculations that take into account interactions with Na^+^. However, the experimentally observed preference is much lower than theoretically predicted. It may again suggest that the interactions with Na^+^ remain weak, which is in agreement with a similar conclusion for cage complexes.

## Conclusions

In conclusion, we synthesized a new type of coordination cage of Ga_3_L_2_ stoichiometry. By the incorporation of amino acids into the ligand structure, we induced chirality on metal centers and obtained chiral cages with excellent diastereoselectivity. The system containing a racemic mixture of ligands has the ability to chirally self-sort into enantiomerically pure cages. The flexibility of ligands led to coordination complexes of the pinwheel structure, different from tetrahedral cages obtained from planar rigid ligands. The cages currently obtained have small cavities; however, because of the universal character of the amino acid-based linker, they may be considered as the smallest members of the whole family that can be extended by using longer peptides. Moreover, the functional character of the side chains of amino acids offers further possibilities to tune the properties toward obtaining functional cages.

## Supporting Information Available

Experimental details and procedures, NMR spectra, ECD and UV spectra, crystal structure determinations and refinements, calculations, and X-ray structure of **9a** (PDF)

## Accession Codes

CCDC 2153029 contains the supplementary crystallographic data for this paper. These data can be obtained free of charge via www.ccdc.cam.ac.uk/data_request/cif, or by emailing data_request@ccdc.cam.ac.uk, or by contacting The Cambridge Crystallographic Data Centre, 12 Union Road, Cambridge CB2 1EZ, UK; fax: +44 1223 336033.
