# Supplementary material for: Toward Coordination Cages with Hybrid Chirality: Amino Acid-Induced Chirality on Metal Centers
Source: Inorg Chem. 2022 Jul 11;61(29):11410–8. doi: 10.1021/acs.inorgchem.2c01738 (PMC9490811; doi:10.1021/acs.inorgchem.2c01738)
Supplement: Supplementary file 1 — ic2c01738_si_001.pdf [file ic2c01738_si_001.pdf]

Toward coordination cages with hybrid chirality - amino acid-induced chirality on metal centers

Marcin Grajda[a], Grzegorz Staros[a], Hanna Jędrzejewska[a,]*, and Agnieszka Szumna[a,]*

* email: agnieszka.szumna@icho.edu.pl

[a] Institute of Organic Chemistry, Polish Academy of Sciences, Kasprzaka 44/52, 01-224 Warsaw, Poland

Electronic Suplementary Information

General information

All solvents and chemicals used were purchased from Merck, TCI Europe N. V., Roth, Chem Impex Inc. and Euriso-top, were of reagent grade and were used without further purification. High resolution ESI and APCI mass spectra were recorded on a MaldiSYNAPT G2-S HDMS spectrometer. High resolution EI mass spectra were recorded on an AutoSpec Premier spectrometer. ^{1}H and ^{13}C NMR spectra were recorded on Bruker 400 MHz, Varian 500 MHz and Varian 600 MHz instruments with residual solvent signal as internal standard. All 2D NMR spectra were recorded at 298 K on Varian 600 MHz with residual solvent signal as internal standard. *J* values are given in Hz. ECD spectra were recorded on Jasco J-715 spectropolarimeter. Specific rotations were measured on Jasco P-2000 polarimeter. $[\alpha]_D$ are given in $degcm^3g^{-1}dm^{-1}$. IR spectra were recorded on an FTIR Jasco 6200 spectrometer.

Synthetic procedures and analytical data

S-**5a**

1,3,5-Benzenetricarboxylic acid **3** (1.33 mmol, 0.28 g) was dissolved in DMF (50 mL) and cooled to 0°C. HOBt hydrate (4.0 mmol, 0.61 g), *S*-PheOMe·HCl **4a** (4.0 mmol, 0.86 g), triethylamine (8.39 mmol, 1.17 mL) and EDCI (4.39 mmol, 0.84 g) were added and the mixture was stirred overnight at room temperature. The solvent was evaporated and water was added to the yellow oil. The white precipitate was collected and washed with distilled water and saturated aqueous $NaHCO_3$. The white powder was dried under reduced pressure. Yield 0.89 g, 95 %.
^{1}H NMR (500 MHz, dimethyl sulfoxide-d6, 298K): δ= 9.19 (d, *J* = 7.7 Hz, 3H), 8.37 (s, 3H), 7.32-7.24 (m, 12H), 7.23-7.19 (m, 3H), 4.74-4.66 (m, 3H), 3.64 (s, 9H), 3.18 (dd, *J* = 13.8, 5.4 Hz, 3H), 3.12 (dd, *J* = 13.8, 9.8 Hz, 3H). ^{13}C NMR (125 MHz, dimethyl sulfoxide-d6, 298K): δ= 171.96, 165.55, 137.57, 134.17, 129.24, 129.00, 128.27, 126.51, 54.44, 51.99, 36.16. HRMS (ESI) *m/z* calcd for $C_{39}H_{39}N_3O_9Na$: 716.2584 $[M+Na]^+$, found 716.2557.

S-**6a**

S-**5a** (1.0 mmol, 0.69 g) was dissolved in methanol (25 mL). Hydrazine hydrate (30 mmol, 1.46 mL) was added and the mixture was heated at 70°C in a sealed tube overnight with stirring. After cooling, the white precipitate was collected, washed with methanol and dried under reduced pressure. Yield 0.59 g, 86 %. ^{1}H NMR (500 MHz, dimethyl sulfoxide-d6, 298K): δ= 9.35 (s, 3H), 8.77 (d, *J* = 8.5 Hz, 3H), 8.25 (s, 3H), 7.35-7.12 (m, 15H), 4.76-4.69 (m, 3H), 4.27 (br s, 6H), 3.09-2.95 (m, 6H). ^{13}C NMR (125 MHz, dimethyl sulfoxide-d6, 298K): δ= 170.30, 165.41, 138.06, 134.32, 129.12, 128.14, 126.30, 53.62, 37.66. HRMS (ESI) *m/z* calcd for $C_{36}H_{39}N_9O_6Na$: 716.2921 $[M+Na]^+$, found 716.2912.

S-**1a**

S-**6a** (0.216 mmol, 0.150 g) was dissolved in methanol (10 mL). Salicyl aldehyde **7** (3.24 mmol, 0.34 mL) was added and the mixture was heated at 70°C in a sealed tube overnight with stirring. After cooling, the white precipitate was collected, washed with methanol and dried under reduced pressure. Yield 0.19 g, 88 %. $[\alpha]_D^{22}$= 173.2 (c=1 in DMSO). The product was obtained as a mixture of two diastereoisomers in 2:1 ratio. Main diastereoisomer ^{1}H NMR (600 MHz, dimethyl sulfoxide-d6, 298K): δ= 11.93 (s, 3H, NH^1), 11.07-11.04 (m, 3H, *OH*), 9.07 (t, *J* = 7.7 Hz, 3H, NH^2), 8.44 (s, 3H, *e*), 8.36 (s, 3H, *b*), 7.55-7.52 (m, 3H, *i*), 7.39-7.35 (m, 6H, *Ph*), 7.31-7.24 (m, 9H, *Ph*), 7.21-7.15 (m, 3H, *j*), 6.94-6.88 (m, 6H, *k*+*h*), 4.85-4.77 (m, 3H, α), 3.20-3.04 (m, 6H, β). ^{13}C NMR (150 MHz, dimethyl sulfoxide-d6, 298K): δ= 167.5 (*d*), 165.8 (*c*), 157.3 (*g*), 147.6 (*e*), 137.8 (*Ph*), 134.2 (*a*), 131.4 (*j*), 129.3 (*b*), 129.2 (*Ph*), 129.0 (*i*), 128.2 (*Ph*), 126.45 (*Ph*), 119.3 (*k*), 118.6 (*f*), 116.3 (*h*), 54.3 (α), 37.1 (β). Minor diastereoisomer ^{1}H NMR (600 MHz, dimethyl sulfoxide-d6, 298K) : δ= 11.47 (s, 3H, NH^1), 10.07 (s, 3H, *OH*), 8.92 (t, *J* = 8.7 Hz, 3H, NH^2), 8.37 (s, 3H, *e*), 8.34 (s, 3H, *b*), 7.75-7.72 (m, 3H, *i*), 7.39-7.35 (m, 6H, *Ph*), 7.31-7.24 (m, 9H, *Ph*), 7.21-7.15 (m, 3H, *j*), 6.94-6.88 (m, 6H, *k*+*h*), 5.63-5.57 (m, 3H, α), 3.20-3.04 (m, 6H, β). ^{13}C NMR (150 MHz, dimethyl sulfoxide-d6, 298K): δ= 172.2 (*d*), 165.6 (*c*), 156.4 (*g*), 141.3 (*e*), 138.2 (*Ph*), 134.5 (*a*), 131.2 (*j*), 129.4 (*b*), 129.2 (*Ph*), 128.2 (*Ph*), 126.38 (*i*), 126.1 (*Ph*), 120.2 (*f*),

119.5 (*k*), 116.2 (*h*), 52.0 (α), 36.3 (β). HRMS (ESI) *m/z* calcd for $C_{57}H_{50}N_9O_9$: 1004.3731 [M-H]$^-$, found 1004.3692. IR (KBr, cm^{-1}): 3640, 3215, 3058, 3029, 2933, 1682, 1641, 1625, 1559, 1531, 1489, 1454, 1362, 1324, 1275, 1237, 1153, 1107, 1078, 1033, 965, 938, 880, 856, 748, 698, 658, 610, 570, 517, 479. Analysis calcd for $C_{57}H_{51}N_9O_9{\cdot}1.5H_2O$: C 66.27, H 5.27, N 12.20, found: C 66.04, H 5.25, N 12.21.

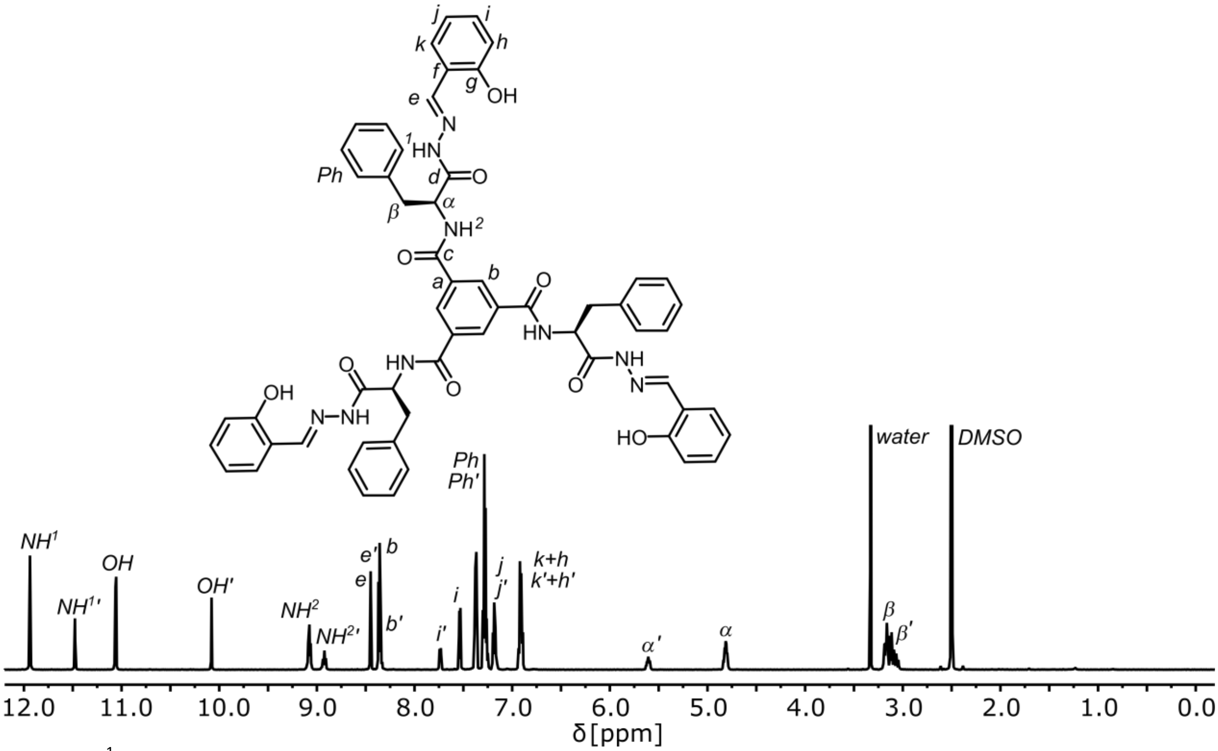

Figure S1. ^{1}H NMR spectrum of *S*-**1a** (dimethyl sulfoxide-d6, 600 MHz, 298 K).

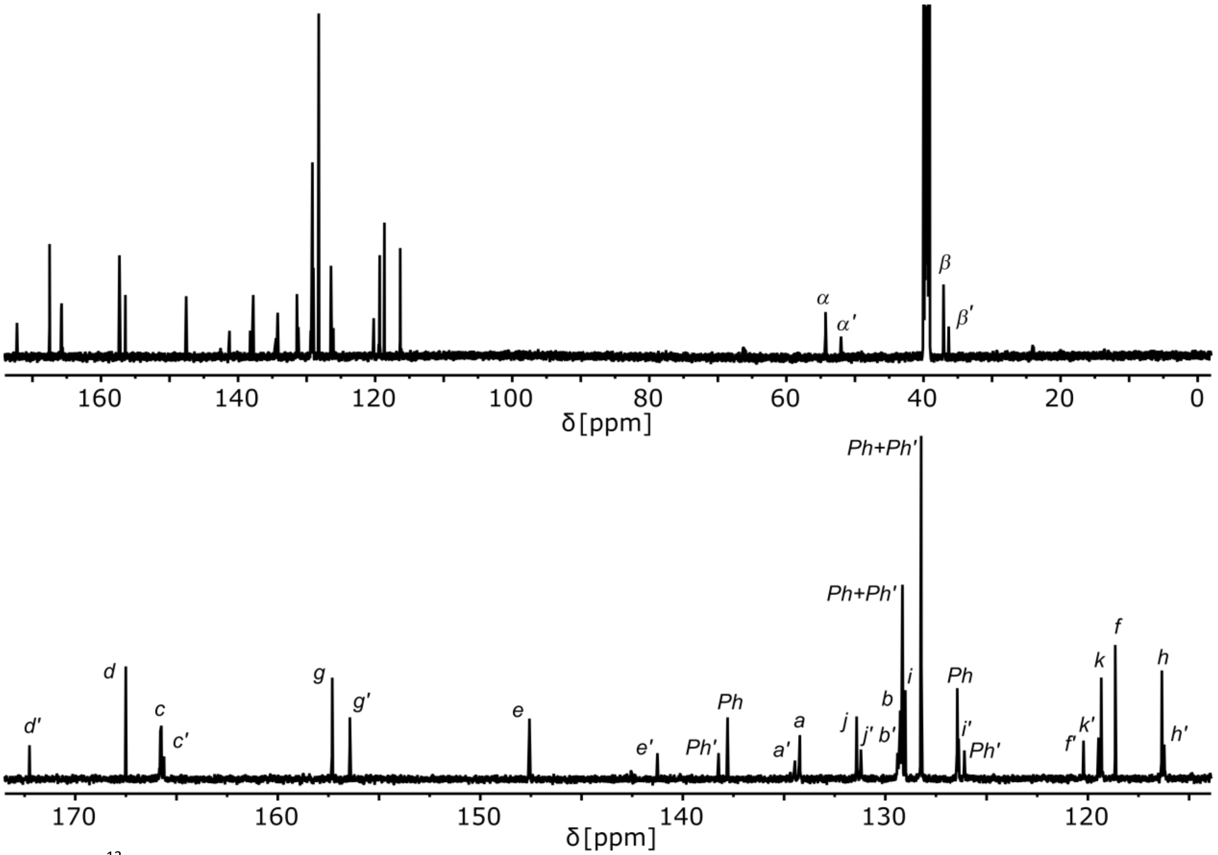

Figure S2. ^{13}C NMR spectrum of *S*-**1a** (dimethyl sulfoxide-d6, 150 MHz, 298 K).

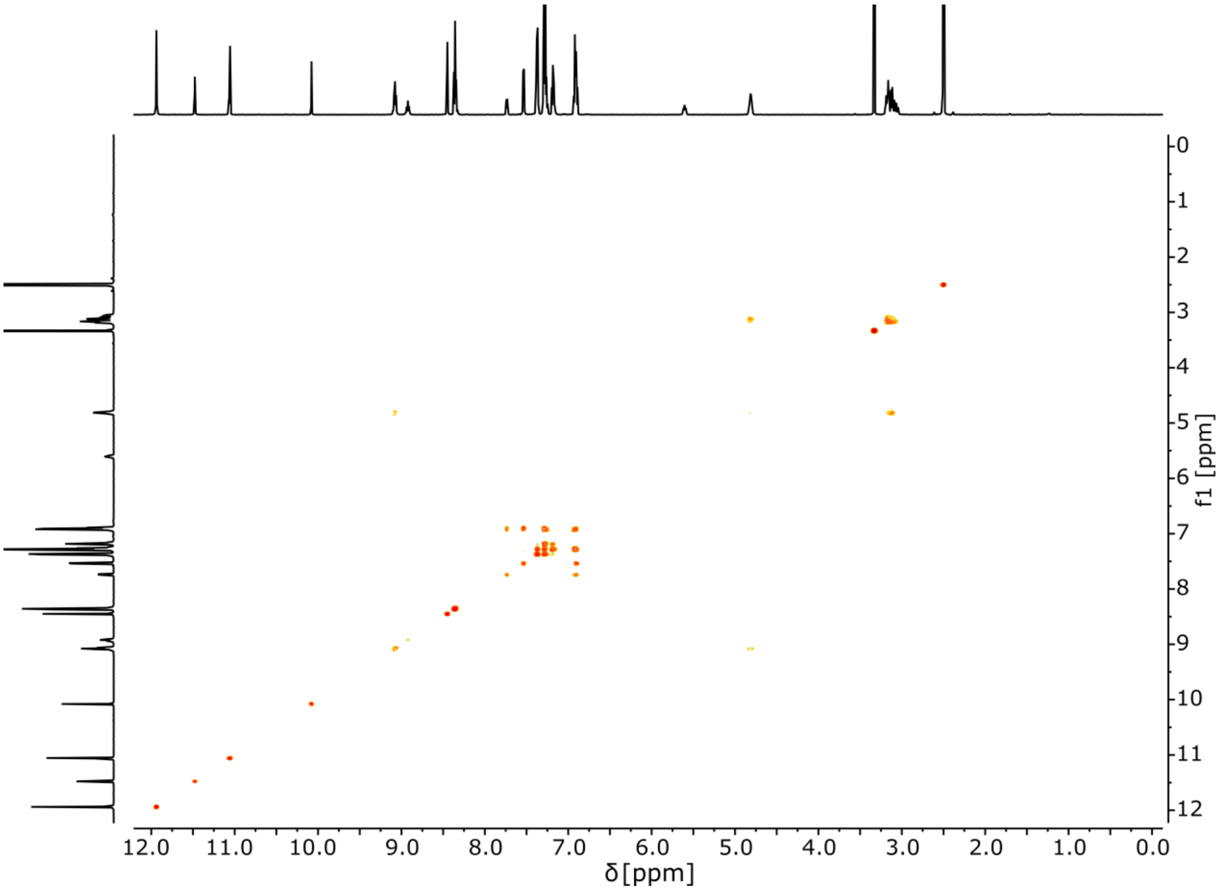

Figure S3. ^{1}H-^{1}H COSY spectrum of *S*-**1a** (dimethyl sulfoxide-d6, 600 MHz, 298 K).

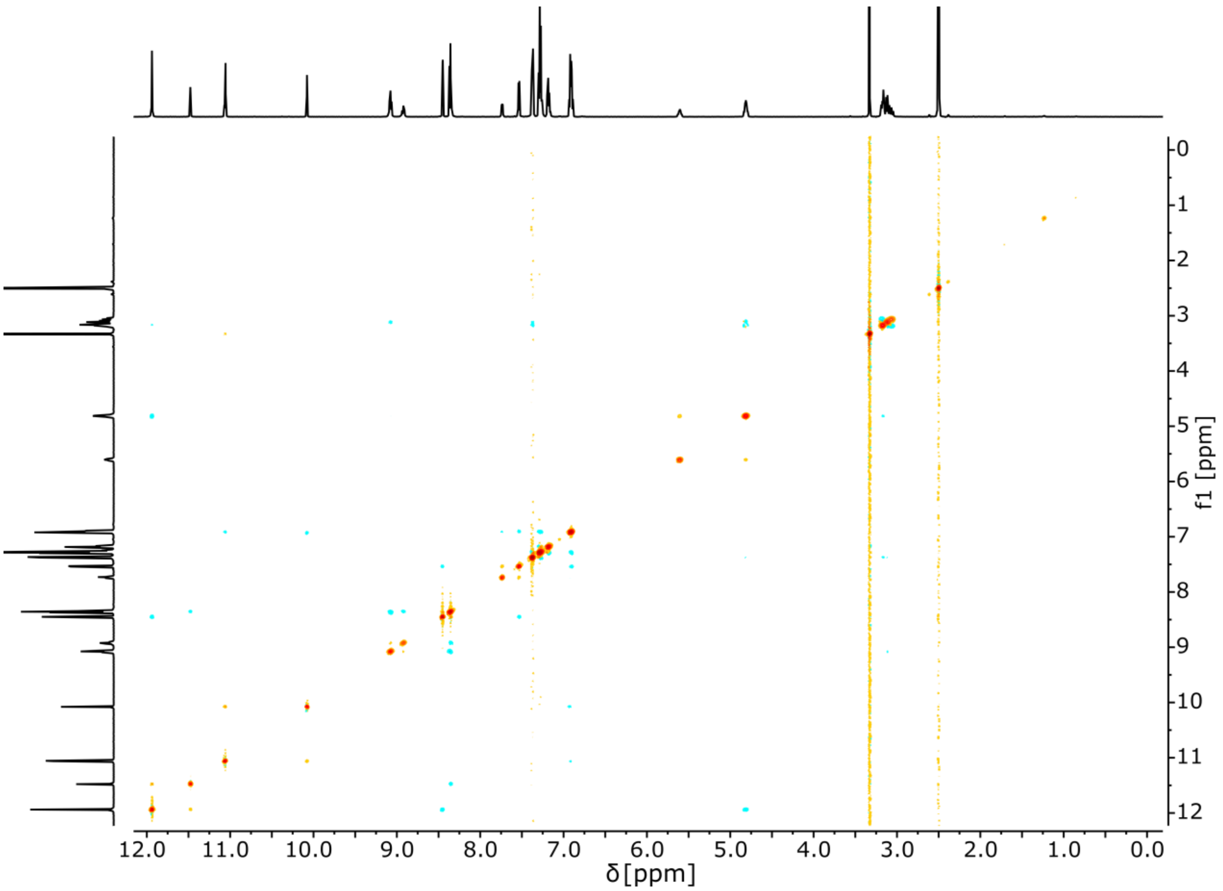

Figure S4. ^{1}H-^{1}H ROESY spectrum of *S*-**1a** (dimethyl sulfoxide-d6, 600 MHz, 298 K).

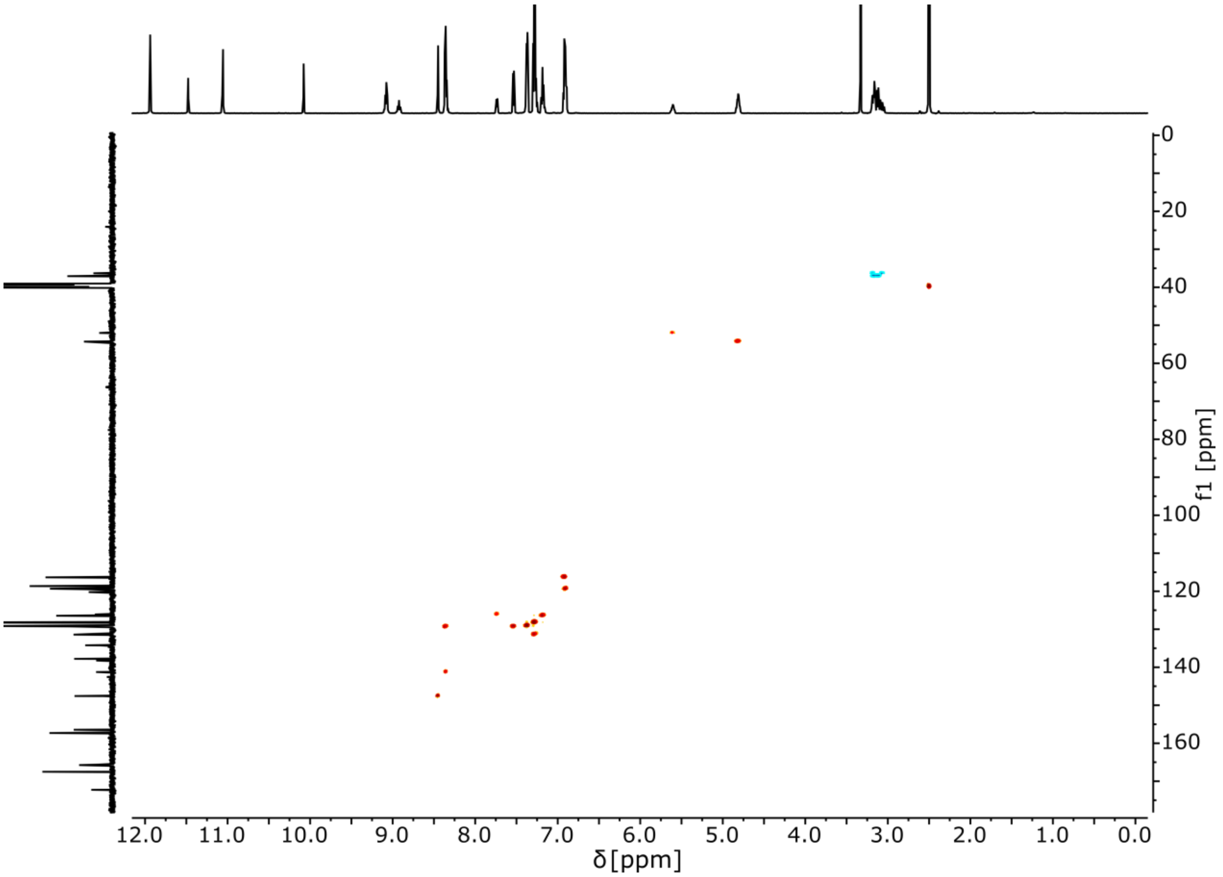

Figure S5. ^{1}H-^{13}C HSQC spectrum of *S*-**1a** (dimethyl sulfoxide-d6, 600 MHz, 298 K).

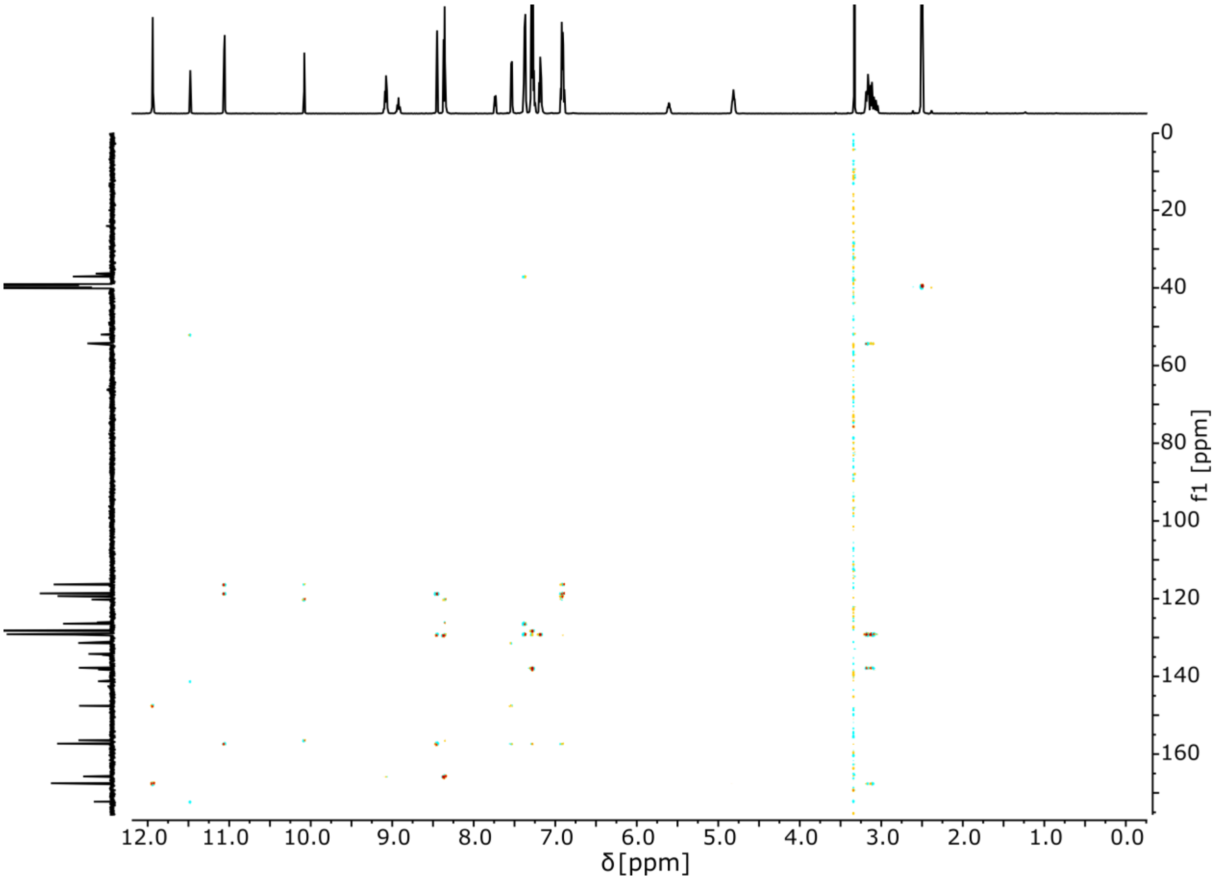

Figure S6. ^{1}H-^{13}C HMBC spectrum of *S*-**1a** (dimethyl sulfoxide-d6, 600 MHz, 298 K).

S-**9a**

S-**1a** (0.06 mmol, 60.4 mg, 2eq.), $Ga(NO_3)_3 \cdot H_2O$ (0.09 mmol, 24.6 mg, 3 eq.) and NaOH (0.36 mmol, 14.4 mg, 12 eq.) were dissolved in methanol (2 mL) and heated at 70°C in a sealed tube overnight. After cooling, the solvent was evaporated and yellow solid was washed with water and dried under reduced pressure. Yield 90 %. ^1H NMR (600 MHz, methanol-d4, 298K): δ= 8.54 (s, 6H, *e*), 7.75 (s, 6H, *b*), 7.34-7.30 (m, 6H, *k*), 7.29-7.25 (m, 6H, *i*), 7.14-7.02 (m, 30H, *Ph*), 6.91-6.87 (m, 6H, *h*), 6.76-6.70 (m, 6H, *j*), 4.86 (dd, *J* = 6.2, 8.1 Hz, 6H, α), 3.11-3.04 (dd, *J* = 8.3, 13.4 Hz, 6H, β), 2.85-2.78 (dd, *J* = 6.2, 13.4 Hz, 6H, β). ^{13}C NMR (150 MHz, methanol-d4, 298K): δ= 173.7 (*d*), 167.3 (*g*), 166.8 (*c*), 158.7 (*e*), 138.4 (*Ph*), 135.4 (*a*), 134.8 (*k*), 134.7 (*i*), 130.7 (*Ph*), 129.1 (*Ph*), 129.0 (*b*), 127.4 (*Ph*), 122.4 (*h*), 119.0 (*f*), 117.0 (*j*), 55.7 (α), 39.9 (β). HRMS (ESI) *m/z* calcd for $C_{114}H_{90}N_{18}O_{18}Ga_3$: 735.1482 $[M]^{3-}$, found 735.1474. IR (KBr, cm^{-1}): 3399, 3060, 3026, 2926, 1660, 1622, 1601, 1538, 1470, 1446, 1402, 1334, 1289, 1199, 1150, 1126, 1092, 1031, 969, 902, 854, 795, 756, 700, 585, 506. Analysis calcd for $C_{114}H_{90}N_{18}O_{18}Ga_3Na_3 \cdot 7H_2O$: C 56.95, H 4.36, N 10.49, found: C 56.98, H 4.35, N 10.73.

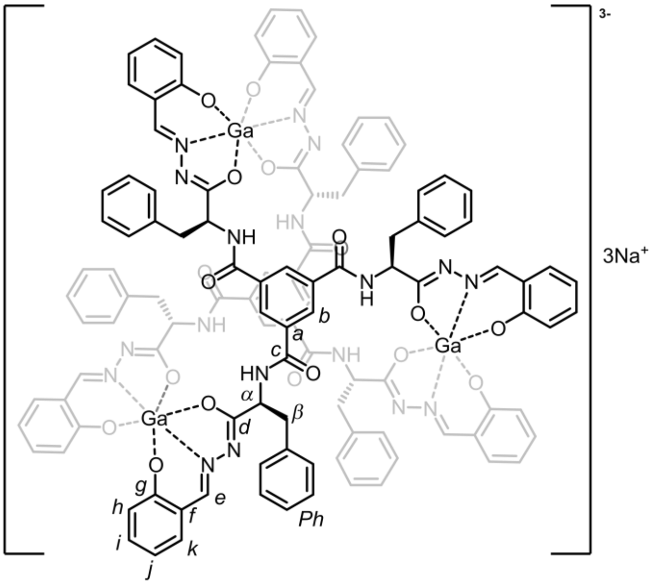

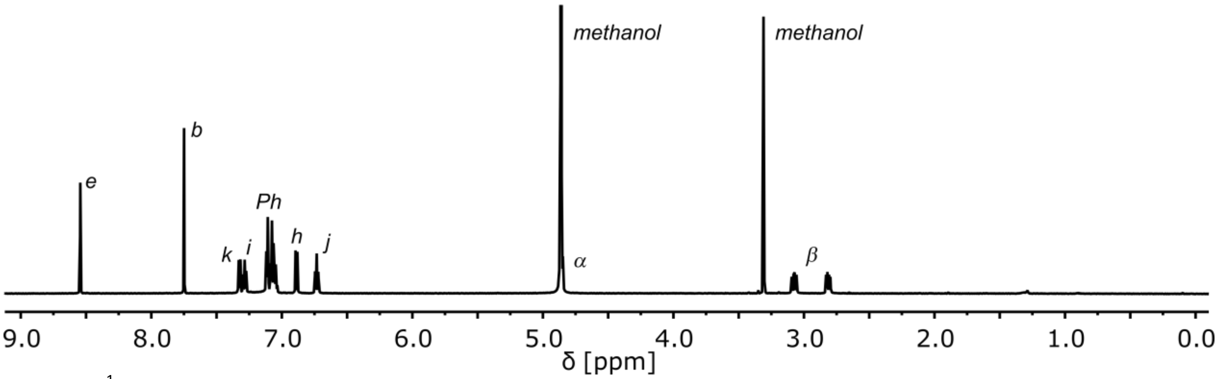

Figure S7. ^1H NMR spectrum of *S*-**9a** (methanol-d4, 600 MHz, 298 K).

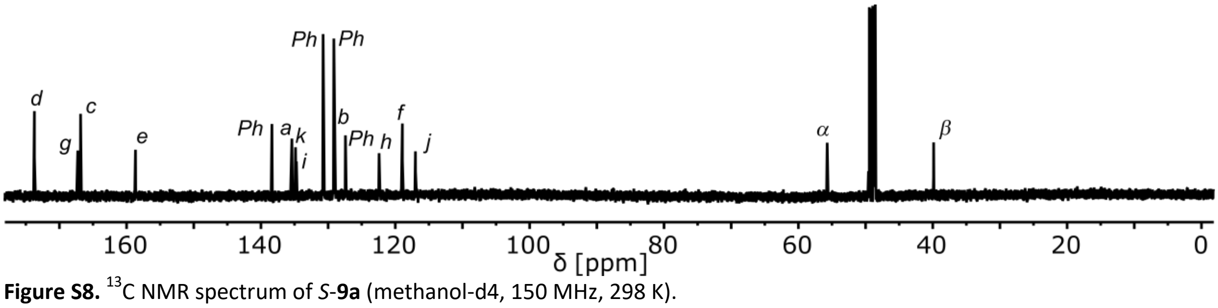

Figure S8. ^{13}C NMR spectrum of *S*-**9a** (methanol-d4, 150 MHz, 298 K).

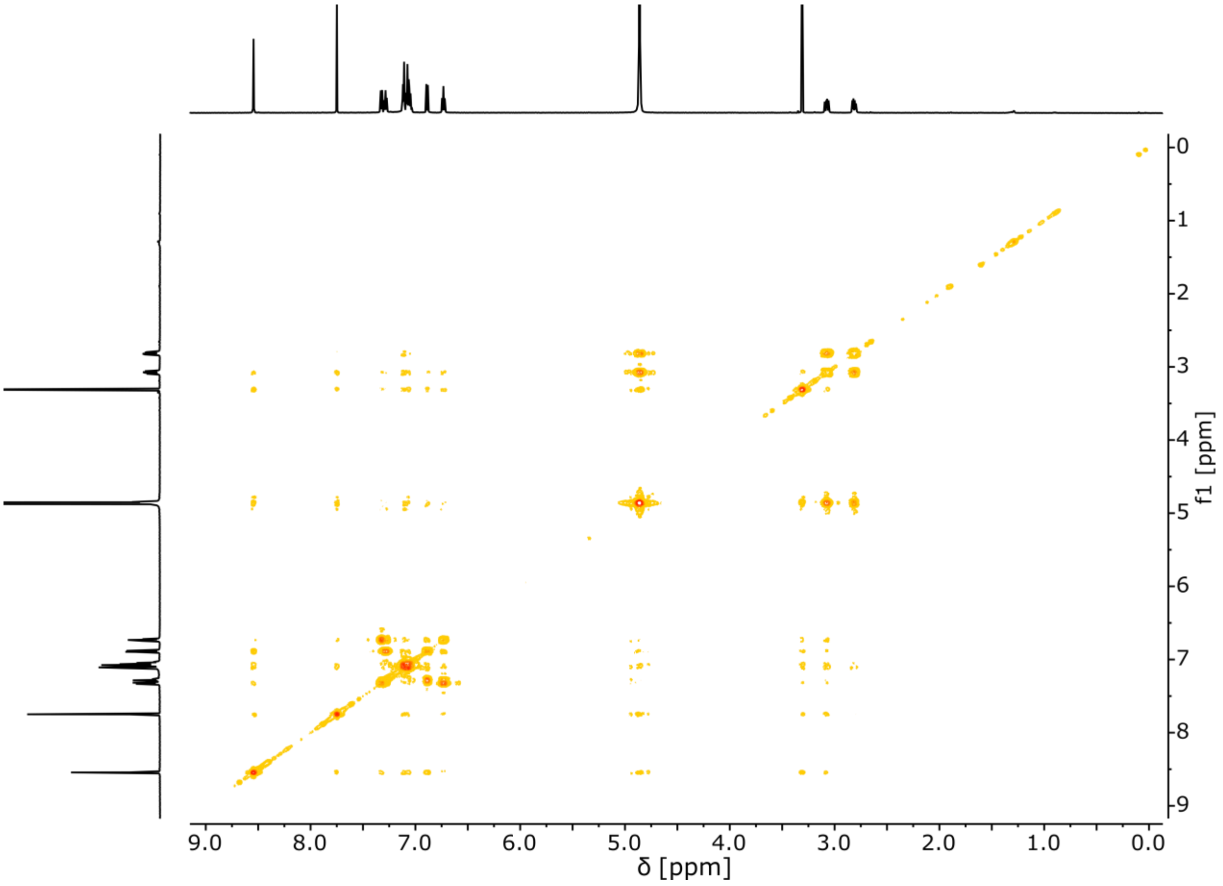

Figure S9. ^{1}H-^{1}H COSY spectrum of *S*-**9a** (methanol-d4, 600 MHz, 298 K).

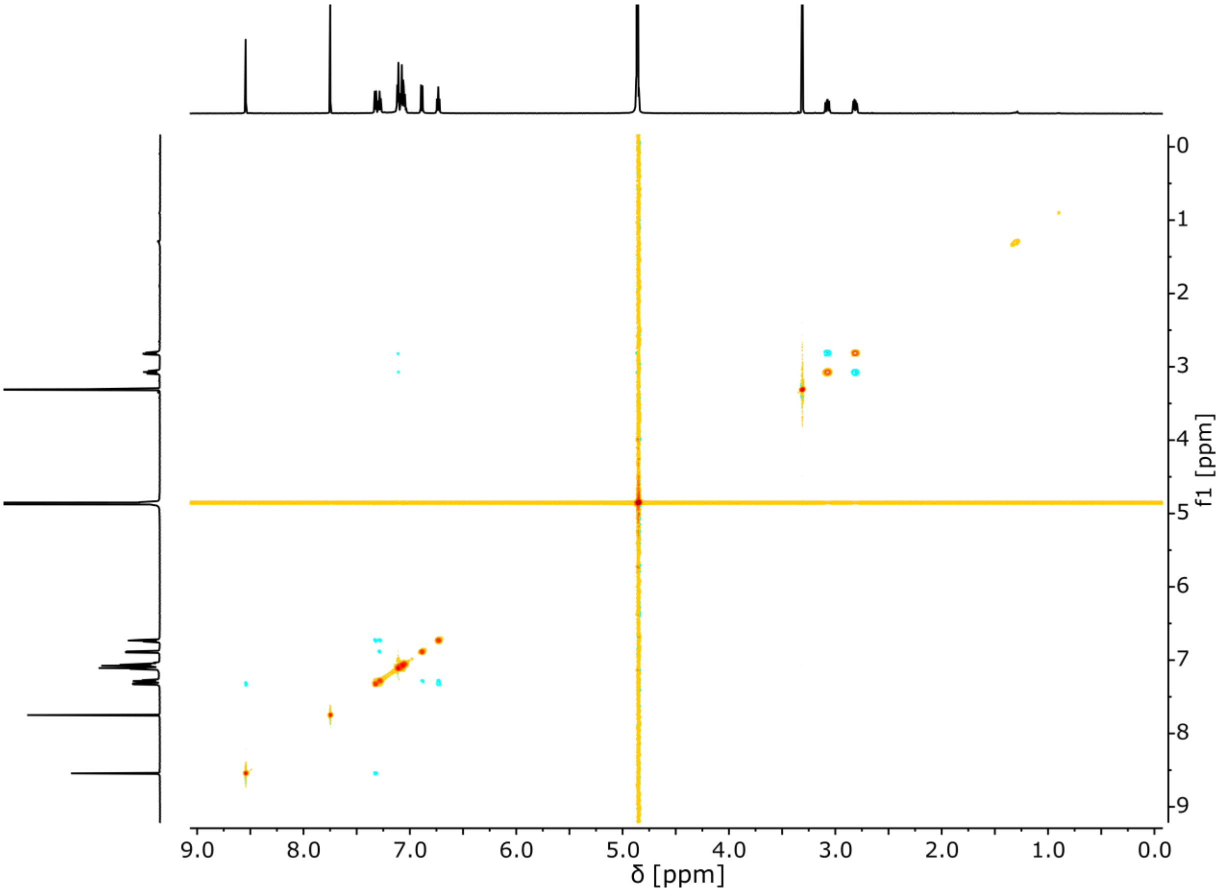

Figure S10. ^{1}H-^{1}H ROESY spectrum of *S*-**9a** (methanol-d4, 600 MHz, 298 K).

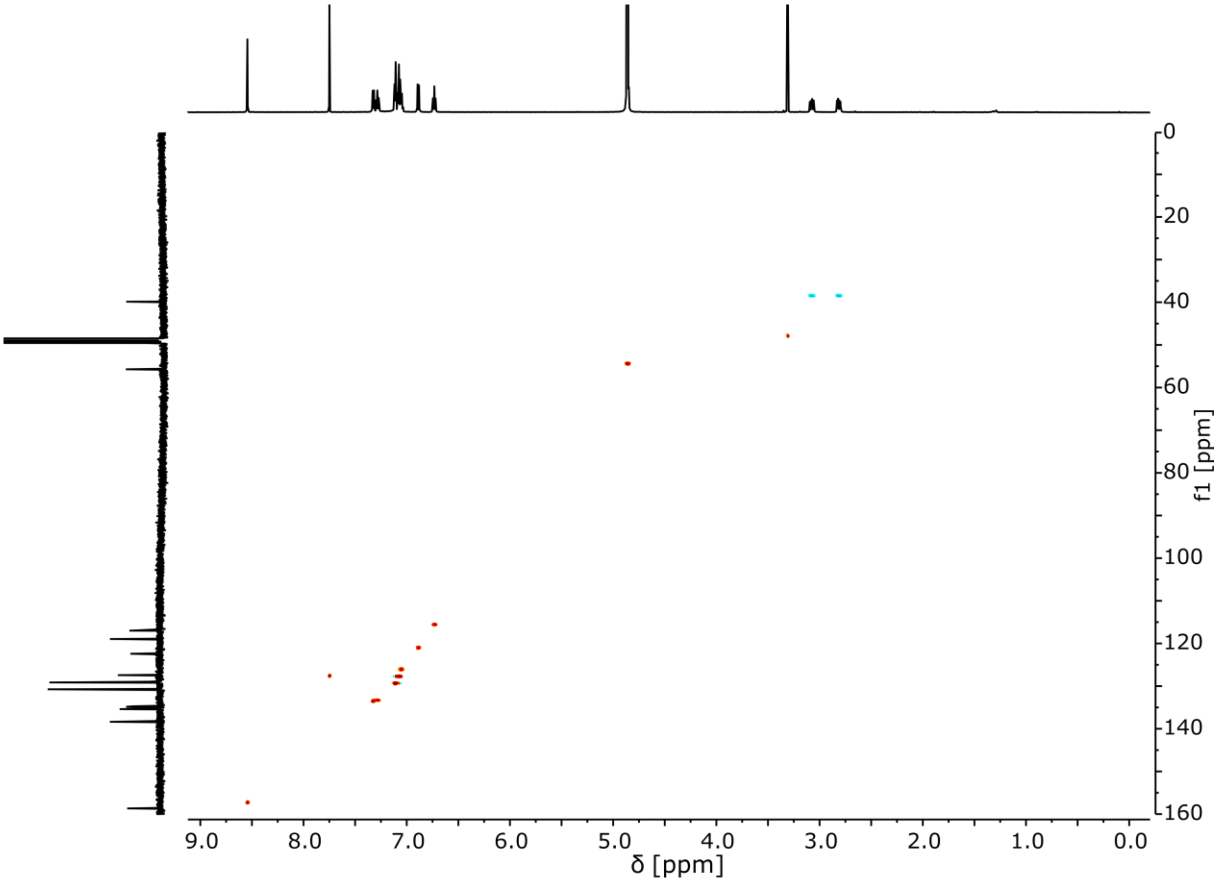

Figure S11. ^{1}H-^{13}C HSQC spectrum of *S*-**9a** (methanol-d4, 600 MHz, 298 K).

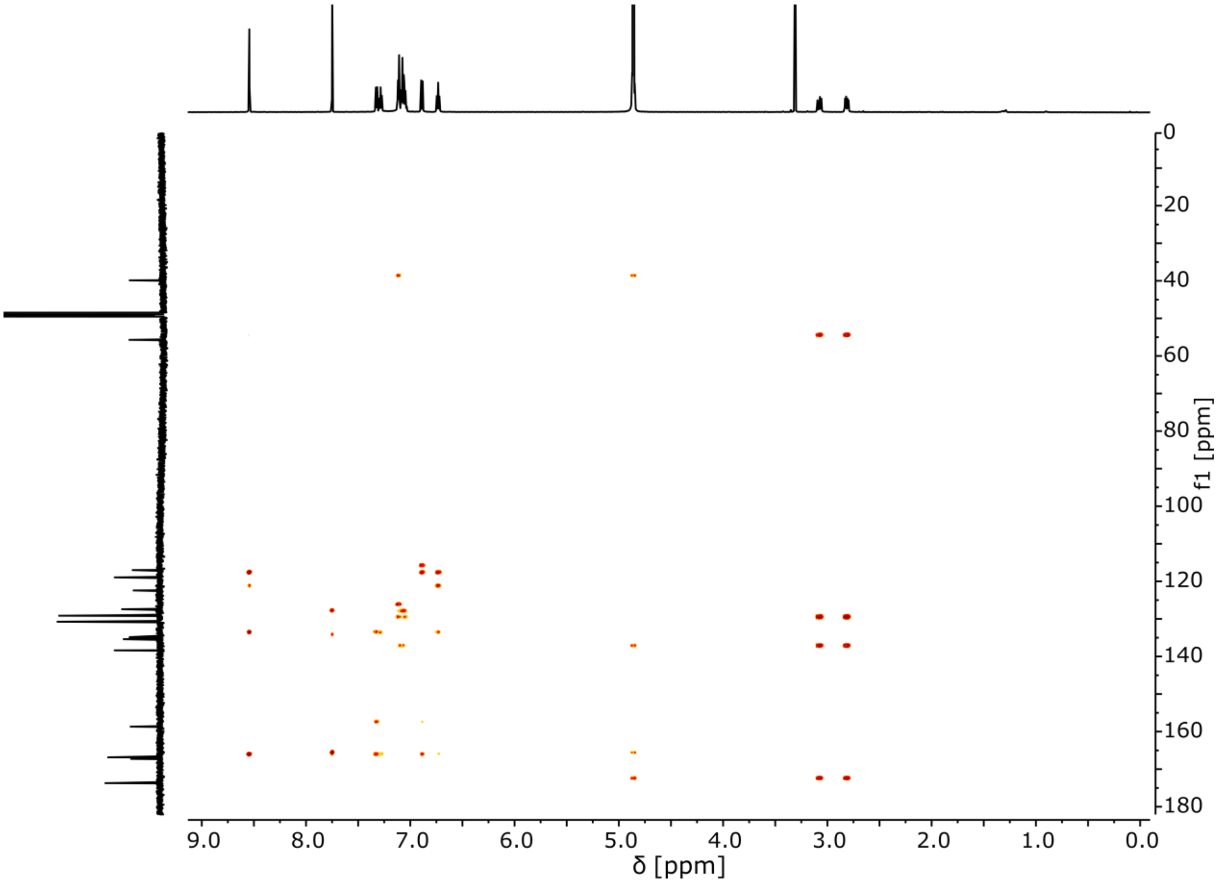

Figure S12. ^{1}H-^{13}C HMBC spectrum of *S*-**9a** (methanol-d4, 600 MHz, 298 K).

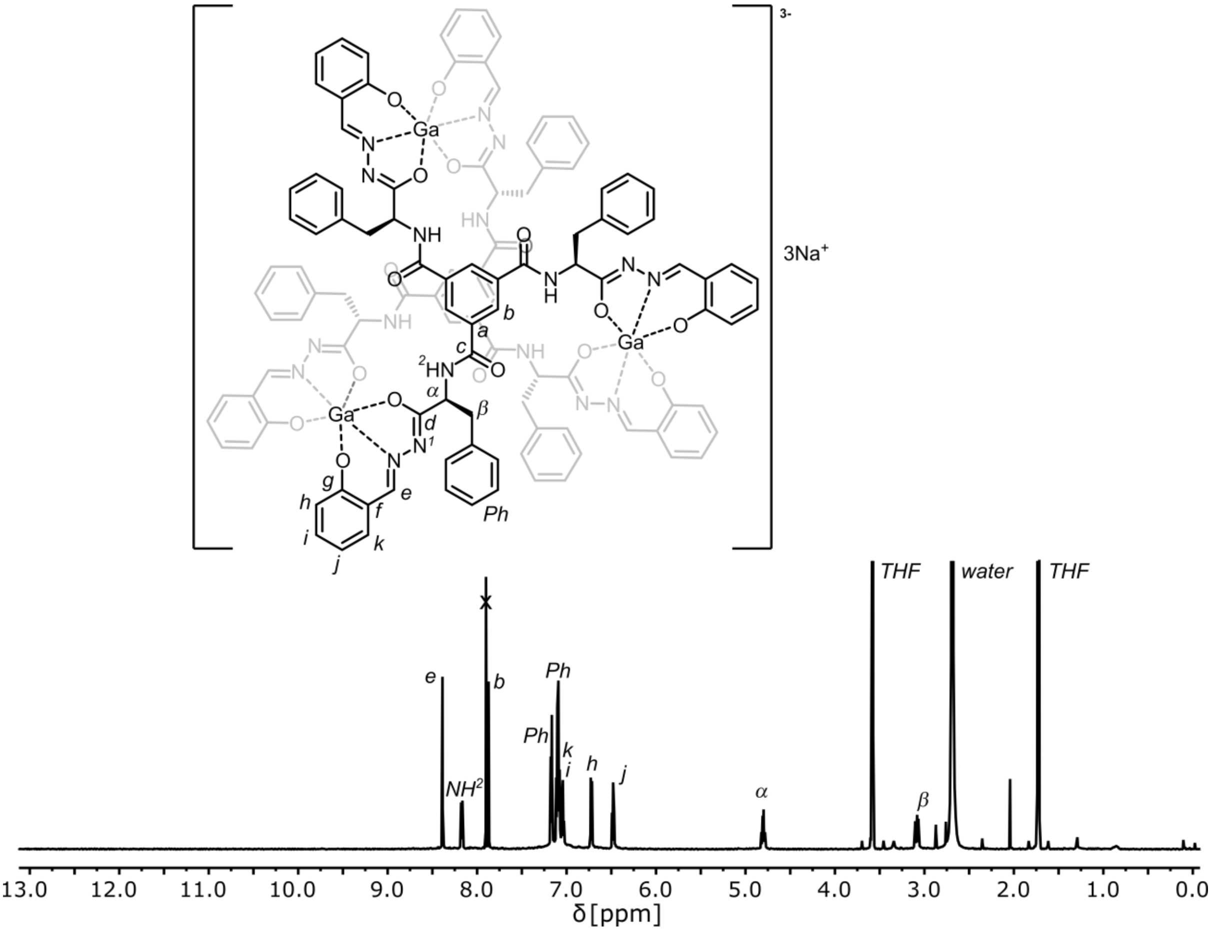

Figure S13. ^{1}H NMR spectrum of *S*-**9a** (tetrahydrofurane-d8, 600 MHz, 298 K, x-$CHCl_3$).

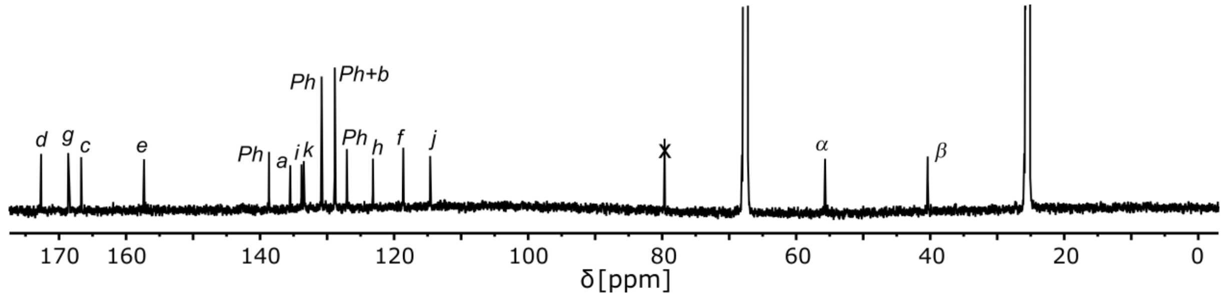

Figure S14. ^{13}C NMR spectrum of *S*-**9a** (tetrahydrofurane-d8, 150 MHz, 298 K, x-$CHCl_3$).

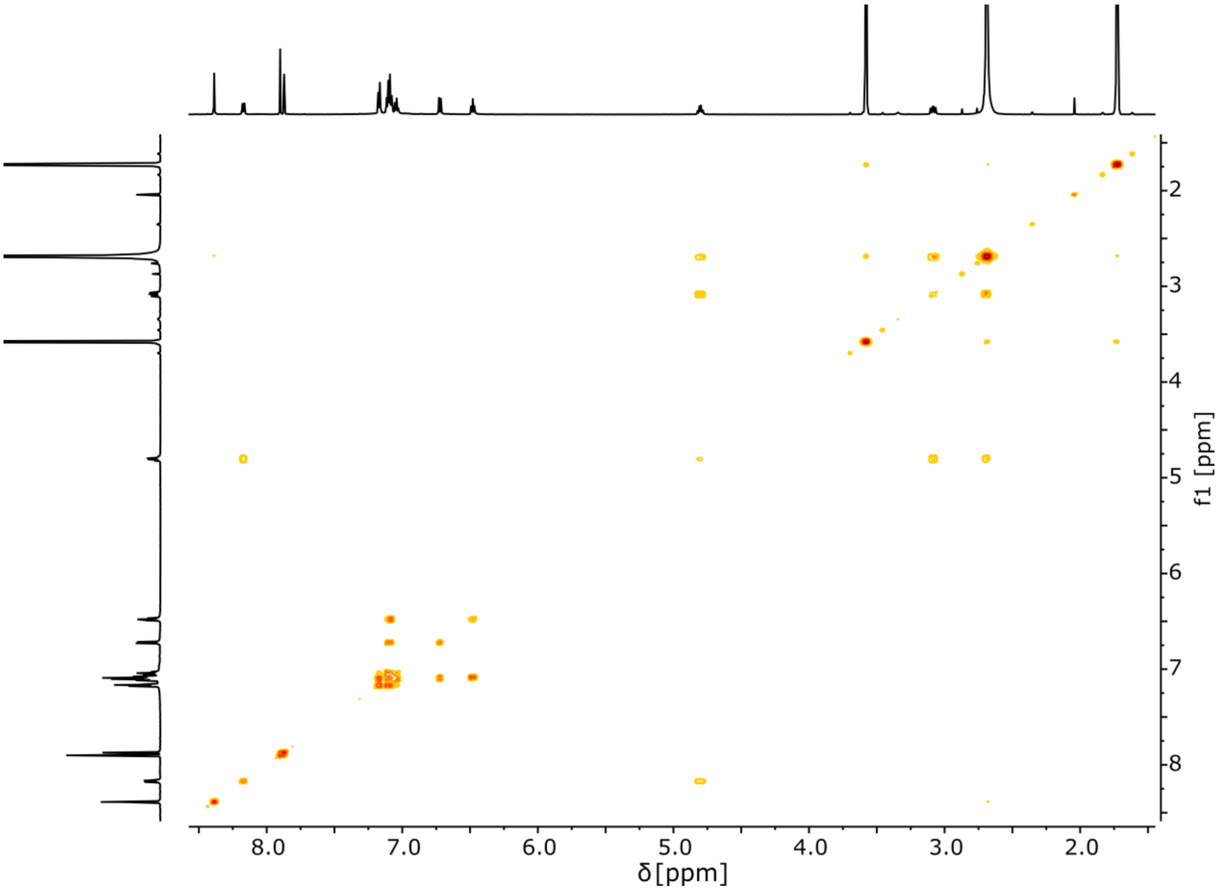

Figure S15. ^{1}H-^{1}H COSY spectrum of *S*-**9a** (tetrahydrofurane-d8, 600 MHz, 298 K).

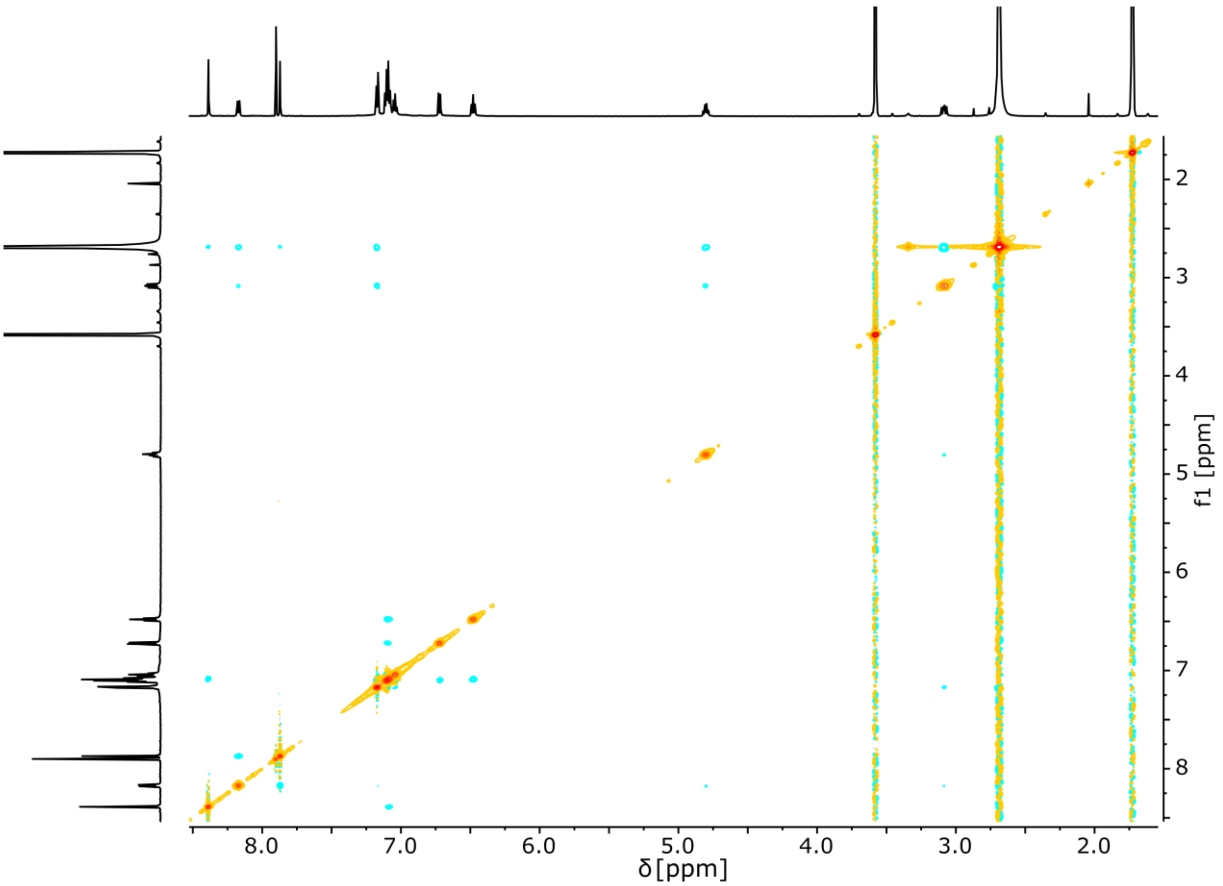

Figure S16. ^{1}H-^{1}H ROESY spectrum of *S*-**9a** (tetrahydrofurane-d8, 600 MHz, 298 K).

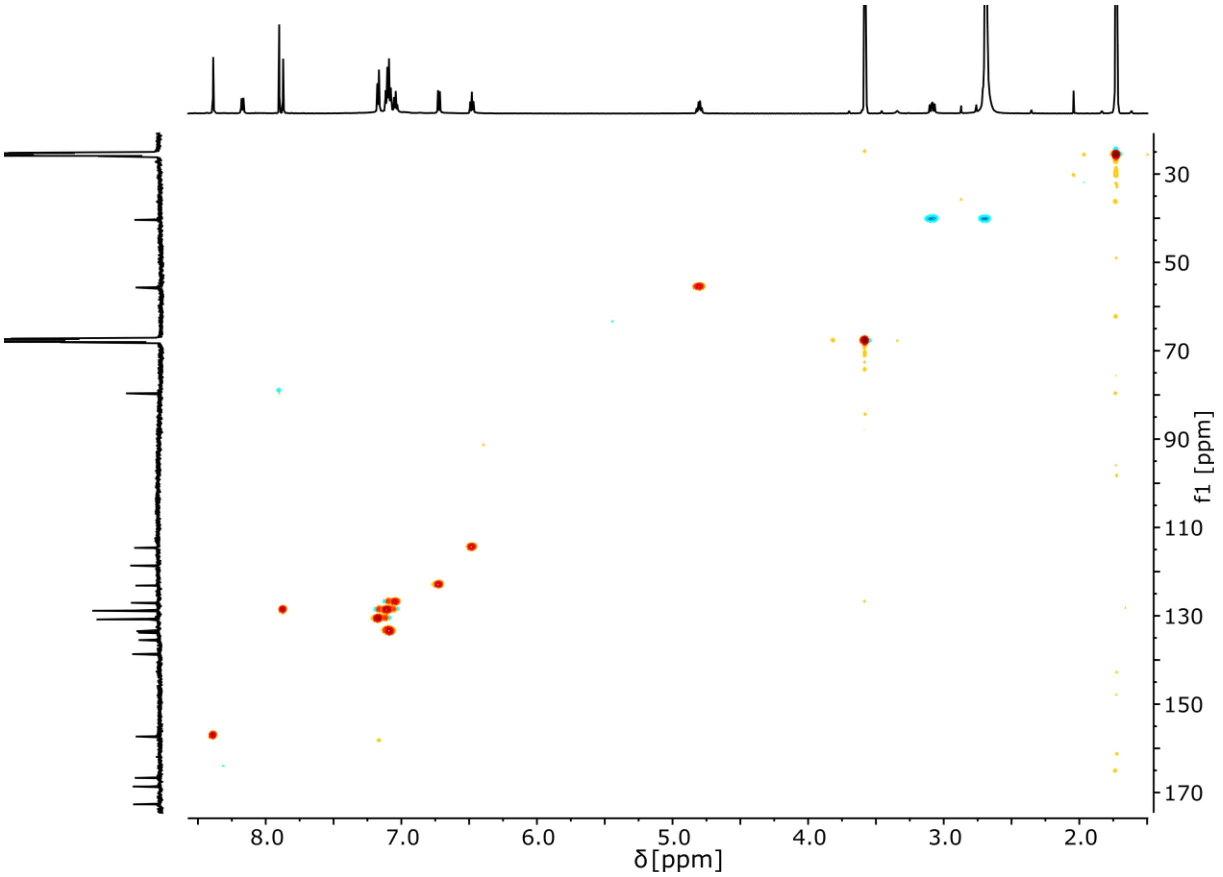

Figure S17. ^{1}H-^{13}C HSQC spectrum of *S*-**9a** (tetrahydrofurane-d8, 600 MHz, 298 K).

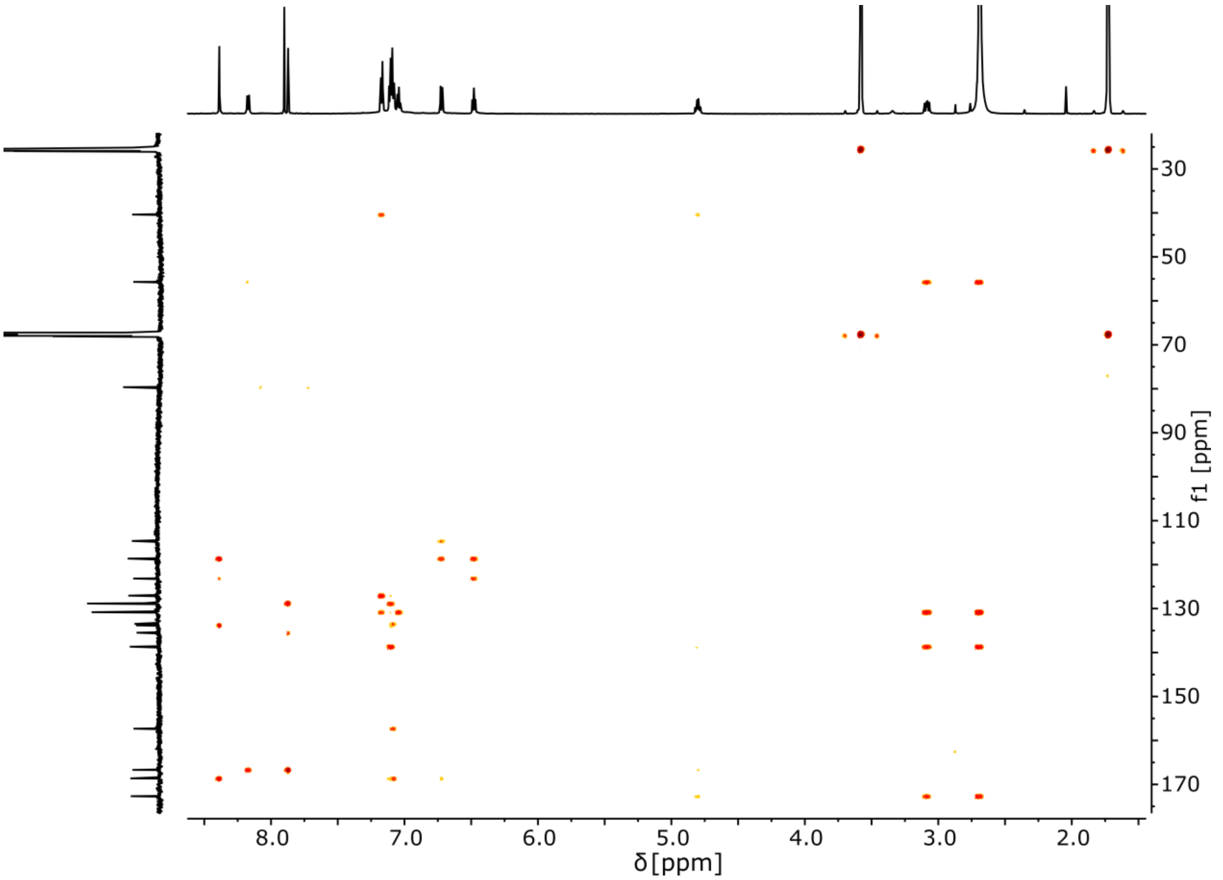

Figure S18. ^{1}H-^{13}C HMBC spectrum of *S*-**9a** (tetrahydrofurane-d8, 600 MHz, 298 K).

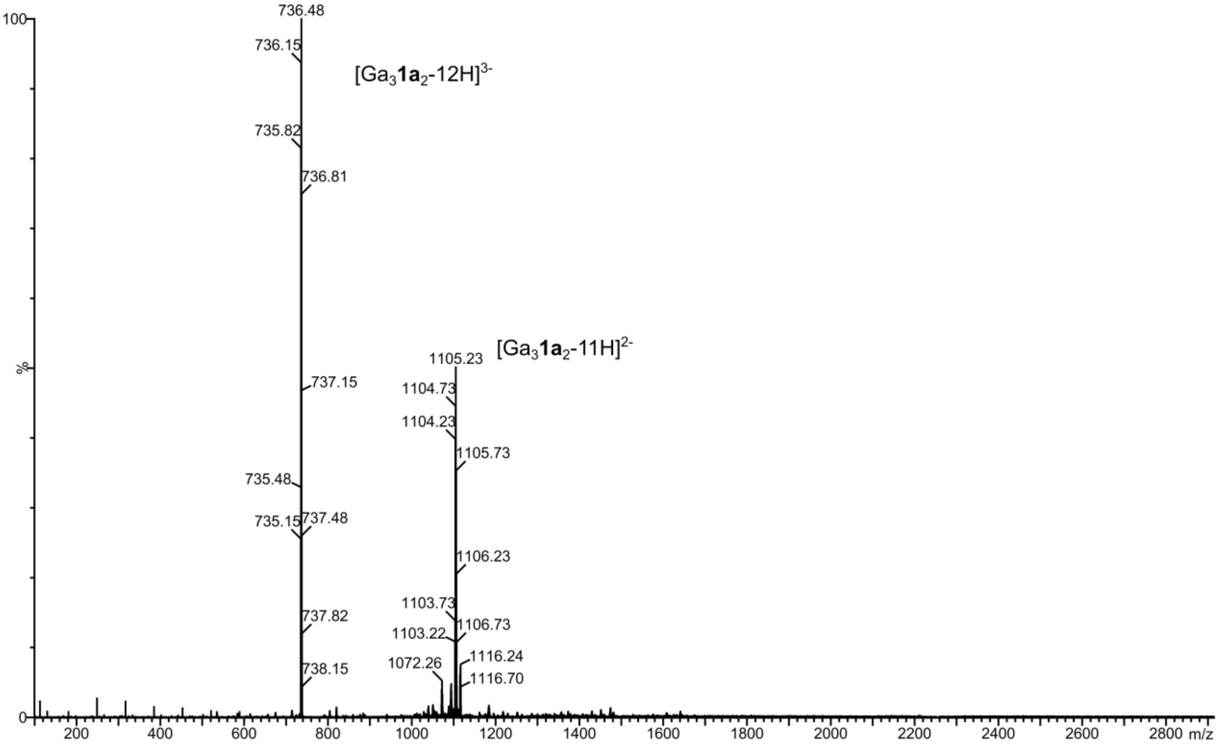

Figure S19. ESI MS spectrum of *S*-**9a**.

S-**5b**

1,3,5-Benzenetricarboxylic acid **3** (1.33 mmol, 0.28 g) was dissolved in DMF (50 mL) and cooled to 0°C. HOBt hydrate (4.0 mmol, 0.61 g), *S*-AlaOMe·HCl **4b** (4.0 mmol, 0.56 g), triethylamine (8.39 mmol, 1.17 mL) and EDCI (4.39 mmol, 0.84 g) were added and the mixture was stirred overnight at room temperature. The solvent was evaporated and water was added to the yellow oil. The white precipitate was collected and washed with distilled water and saturated aqueous $NaHCO_3$. The white powder was dried under reduced pressure. Yield 89 %. ^{1}H NMR (500 MHz, dimethyl sulfoxide-d6, 298K): δ= 9.13 (d, *J* = 6.8 Hz, 3H), 8.50 (s, 3H), 4.54-4.50 (m, 3H), 3.66 (s, 9H), 1.43 (d, *J* = 7.3 Hz, 9H). ^{13}C NMR (125 MHz, dimethyl sulfoxide-d6, 298K): δ= 172.97, 165.46, 134.22, 129.34, 51.93, 48.47, 16.68. HRMS (ESI) *m/z* calcd for $C_{21}H_{27}N_3O_9Na$: 488.1645 $[M+Na]^+$, found 488.1651.

S-**6b**

S-**5b** (1.0 mmol, 0.47 g) was dissolved in methanol (25 mL). Hydrazine hydrate (30 mmol, 1.46 mL) was added and the mixture was heated at 70°C in a sealed tube overnight with stirring. After cooling, the white precipitate was collected, washed with methanol and dried under reduced pressure. Yield 94 %. ^{1}H NMR (500 MHz, dimethyl sulfoxide-d6, 298K): δ= 9.22 (s, 3H), 8.68 (d, *J* = 7.6 Hz, 3H), 8.44 (s, 3H), 4.54-4.50 (m, 3H), 4.26 (s, 6H), 1.33 (d, *J* = 10.6 Hz, 9H). ^{13}C NMR (125 MHz, dimethyl sulfoxide-d6, 298K): δ= 171.46, 165.28, 134.27, 129.29, 47.81, 18.26. HRMS (ESI) *m/z* calcd for $C_{18}H_{26}N_9O_6$: 464.2006 $[M-H]^-$, found 464.2010.

S-**1b**

S-**6b** (0.216 mmol, 0.100 g) was dissolved in methanol (10 mL). Salicyl aldehyde **7** (3.24 mmol, 0.34 mL) was added and the mixture was heated at 70°C in a sealed tube overnight with stirring. After cooling, the white precipitate was collected, washed with methanol and dried under reduced pressure. Yield 80 %. $[\alpha]_D^{22}$= 174.5 (c=1 in DMSO). The product was obtained as a mixture of two diastereoisomers in 2.6:1 ratio. Main diastereoisomer ^{1}H NMR (500 MHz, dimethyl sulfoxide-d6, 298K): δ= 11.83 (s, 3H, NH^1), 11.14-11.10 (m, 3H, *OH*), 8.98-8.95 (m, 3H, NH^2), 8.56 (s, 3H, *e*), 8.46 (s, 3H, *b*), 7.54-7.49 (m, 3H, *i*), 7.32-7.20 (m, 3H, *j*), 6.94-6.85 (m, 6H, *k*+*h*), 4.62-4.55 (m, 3H, α), 1.46 (d, *J* = 7.0 Hz, 9H, β). ^{13}C NMR (125 MHz, dimethyl sulfoxide-d6, 298K): δ= 168.7 (*d*), 165.7 (*c*), 157.3 (*g*), 147.5 (*e*), 134.2 (*a*), 131.3 (*j*), 129.38 (*i*), 129.45 (*b*), 119.3 (*k*), 118.6 (*f*), 116.4 (*h*), 48.5 (α), 17.6 (β).

Minor diastereoisomer ^{1}H NMR (500 MHz, dimethyl sulfoxide-d6, 298K): δ= 11.38 (s, 3H, *NH^{1}*), 10.07 (s, 3H, *OH*), 8.85-8.82 (m, 3H, *NH^{2}*), 8.54 (s, 3H, *e*), 8.33 (s, 3H, *b*), 7.71-7.67 (m, 3H, *i*), 7.32-7.20 (m, 3H, *j*), 6.94-6.85 (m, 6H, *k*+*h*), 5.37-5.30 (m, 3H, α), 1.46 (d, *J* = 6.8 Hz, 9H, β). ^{13}C NMR (125 MHz, dimethyl sulfoxide-d6, 298K): δ= 173.2 (*d*), 165.4 (*c*), 156.4 (*g*), 141.1 (*e*), 134.5 (*a*), 131.1 (*j*), 129.6 (*b*), 126.5 (*i*), 120.2 (*f*), 119.5 (*k*), 116.2 (*h*), 46.3 (α), 16.7 (β). HRMS (ESI) *m/z* calcd for $C_{39}H_{38}N_9O_9$: 776.2792 [M-H]$^-$, found 776.2777. IR (KBr, cm^{-1}): 3220, 3054, 1657, 1622, 1531, 1489, 1453, 1388, 1361, 1272, 1222, 1154, 1101, 1035, 965, 883, 856, 755, 688, 658, 545, 477. Analysis calcd for $C_{39}H_{39}N_9O_9 \cdot H_2O$: C 58.86, H 5.19, N 15.84, found: C 58.63, H 5.13, N 15.72.

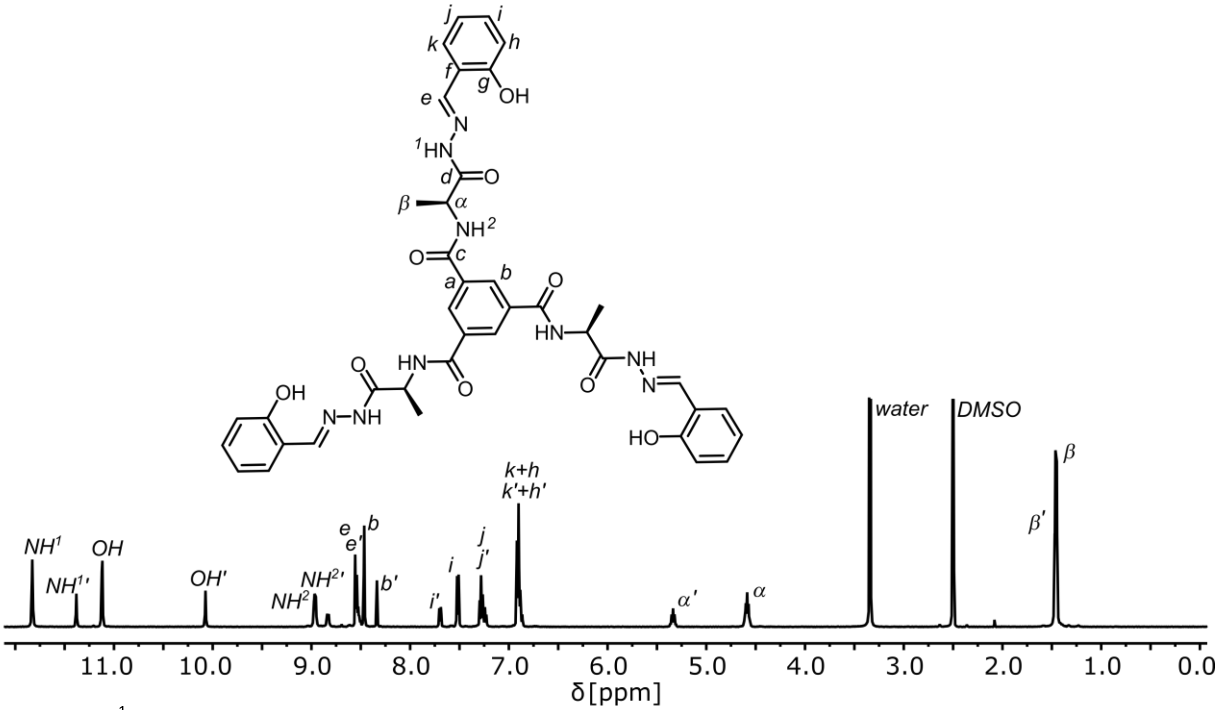

Figure S20. ^{1}H NMR spectrum of *S*-**1b** (dimethyl sulfoxide–d6, 500 MHz, 298 K).

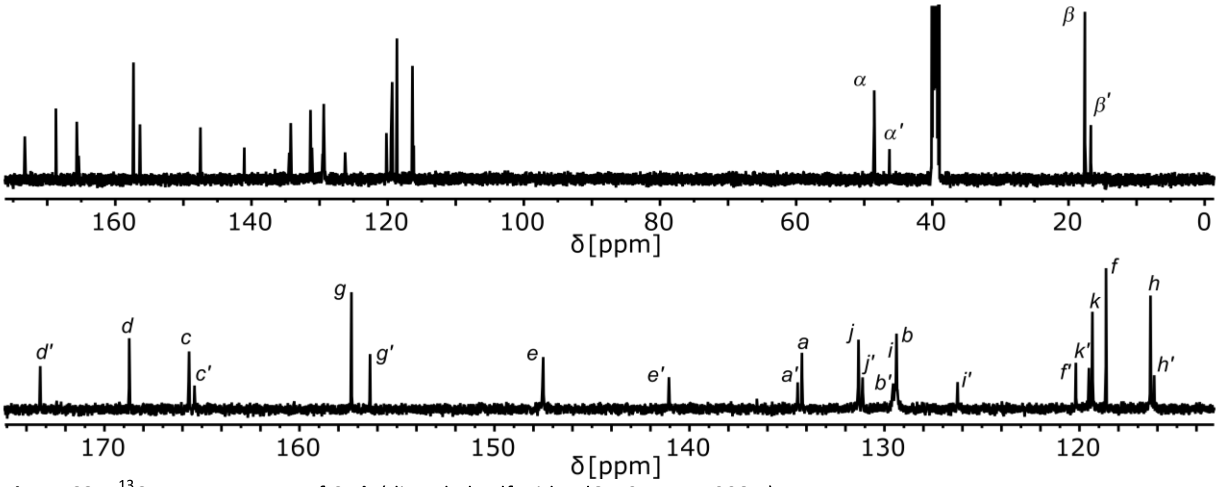

Figure S21. ^{13}C NMR spectrum of *S*-**1b** (dimethyl sulfoxide–d6, 125 MHz, 298 K).

S-**9b**

S-**1b** (0.06 mmol, 46.7 mg, 2eq.), $Ga(NO_3)_3 \cdot H_2O$ (0.09 mmol, 24.6 mg, 3 eq.) and NaOH (0.36 mmol, 14.4 mg, 12 eq.) were dissolved in methanol (2 mL) and heated at 70°C in a sealed tube overnight. After cooling, the solvent was evaporated and yellow solid was washed with water/acetone 1:1 mixture and dried under reduced pressure. Yield 80 %. ^{1}H NMR (600 MHz, methanol-d4, 298K): δ= 8.63 (s, 6H, *e*), 7.82 (s, 6H, *b*), 7.34-7.31 (m, 6H, *k*), 7.23-7.18 (m, 6H, *i*), 6.75-6.67 (m, 12H, *h*+*j*), 4.75 (q, *J* = 7.1 Hz, 6H, α), 1.31 (d, *J* = 7.1 Hz, 12H, β). ^{13}C NMR (150 MHz, methanol-d4, 298K): δ= 175.3

(*d*), 167.7 (*c*), 167.1 (*g*), 158.7 (*e*), 135.6 (*a*), 134.7 (*k*), 134.6 (*i*), 129.0 (*b*), 122.1 (*h*), 118.8 (*f*), 117.1 (*j*), 49.9 (α), 18.9 (β). HRMS (ESI) *m/z* calcd for $C_{78}H_{66}N_{18}O_{18}Ga_3$: 583.0857 $[M]^{3-}$, found 583.0848. IR (KBr, cm^{-1}): 3388, 2427, 1789, 1658, 1624, 1601, 1534, 1472, 1446, 1384, 1291, 1200, 1152, 1126, 1093, 1036, 984, 902, 836, 799, 760, 663, 586, 518, 482, 419.

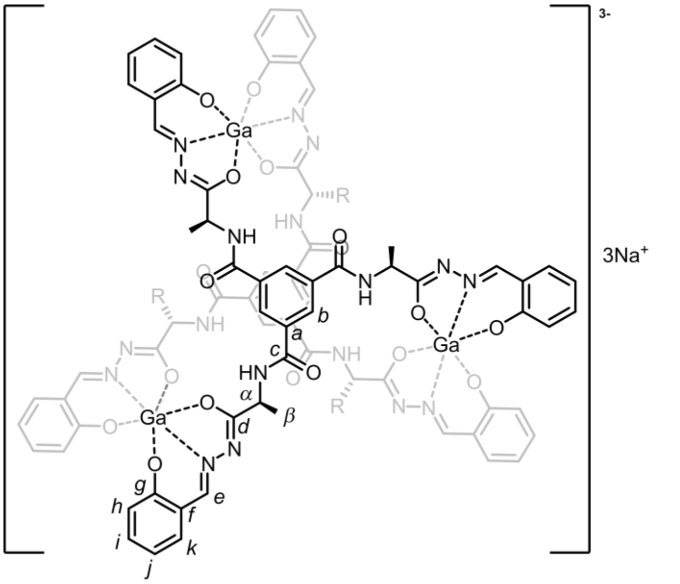

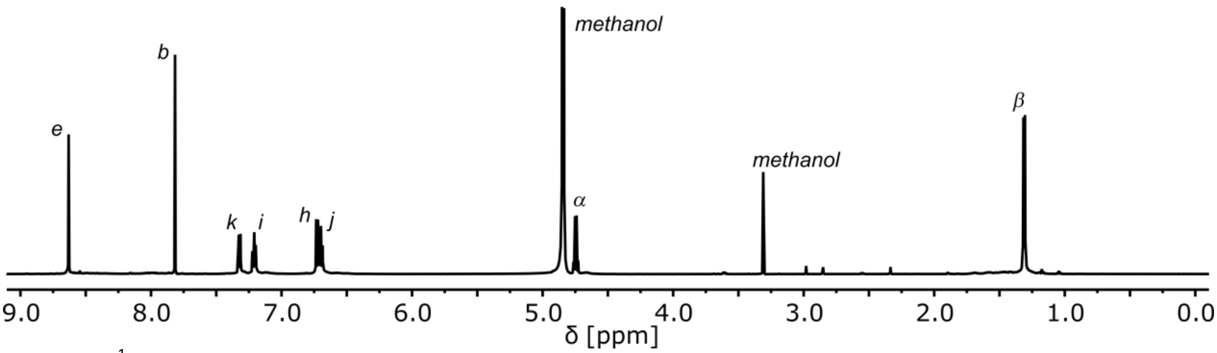

Figure S22. ^{1}H NMR spectrum of *S*-**9b** (methanol-d4, 600 MHz, 298 K).

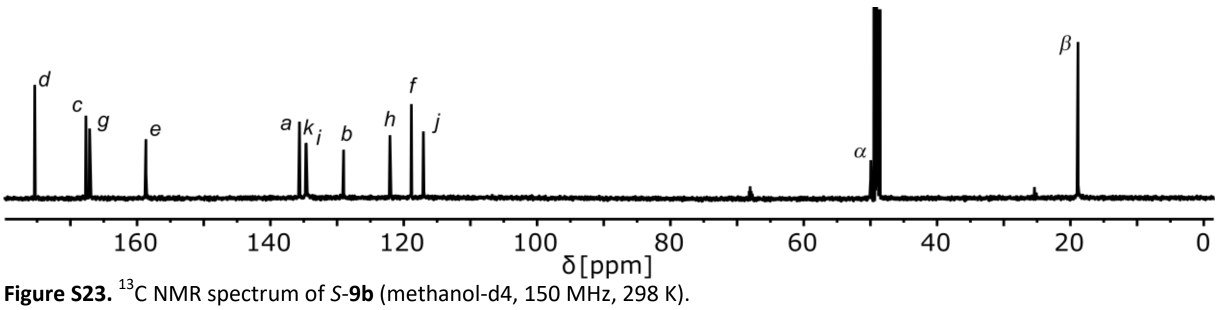

Figure S23. ^{13}C NMR spectrum of *S*-**9b** (methanol-d4, 150 MHz, 298 K).

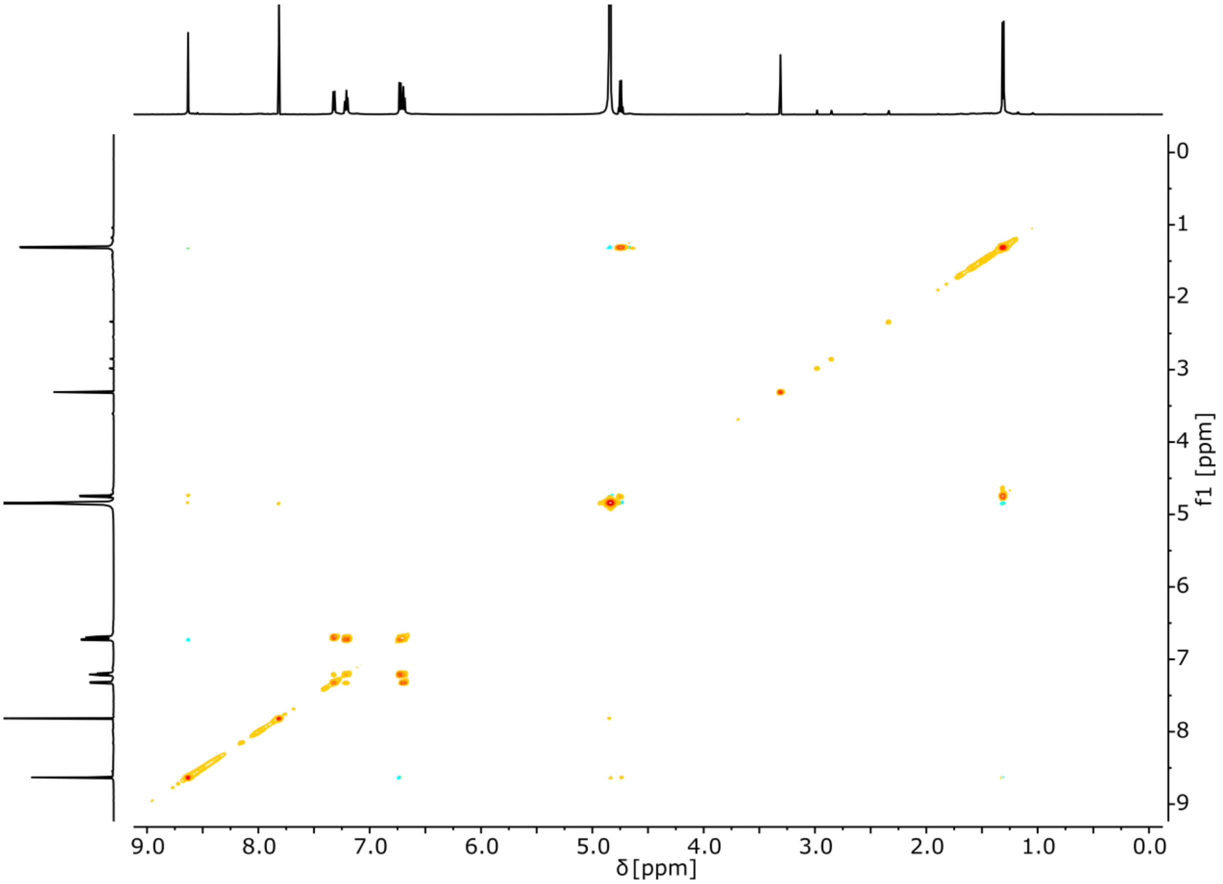

Figure S24. ^{1}H-^{1}H COSY spectrum of *S*-**9b** (methanol-d4, 600 MHz, 298 K).

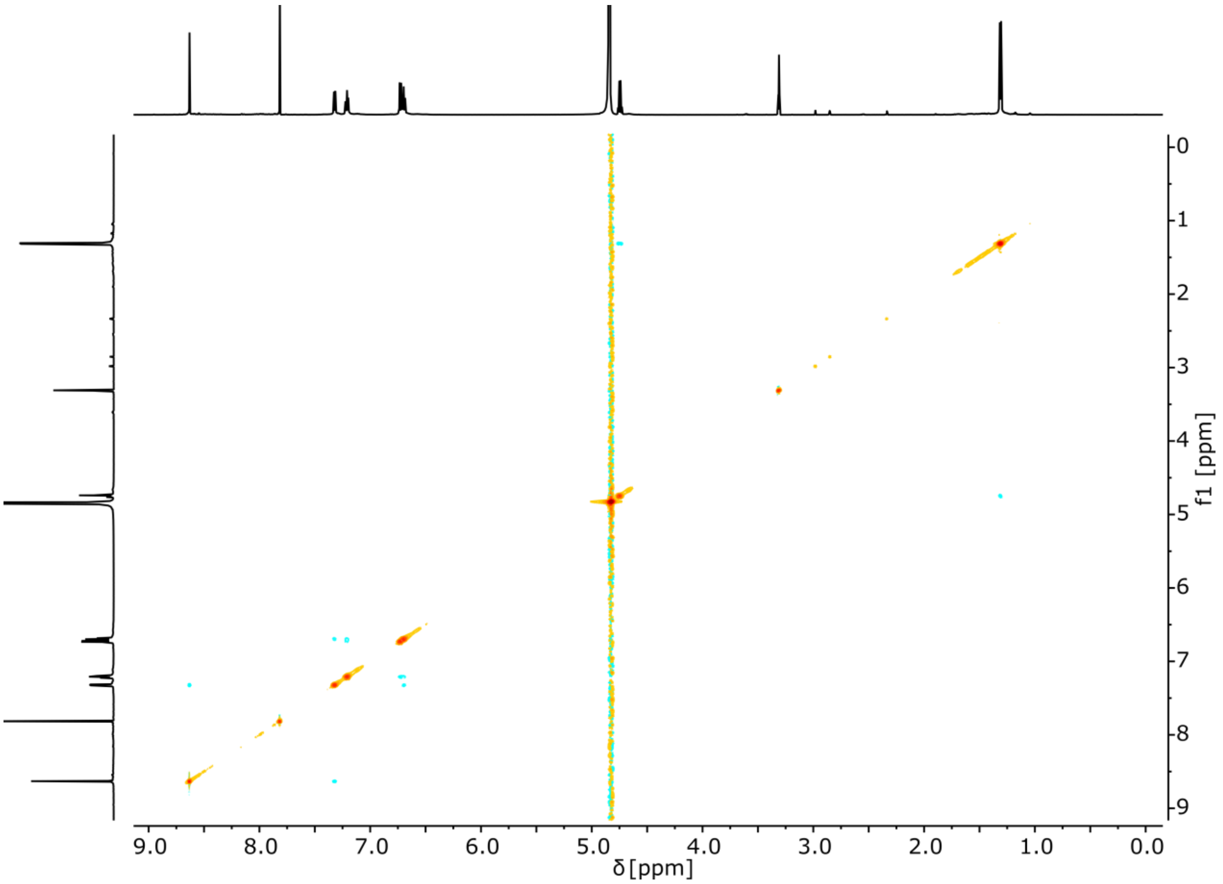

Figure S25. ^{1}H-^{1}H ROESY spectrum of *S*-**9b** (methanol-d4, 600 MHz, 298 K).

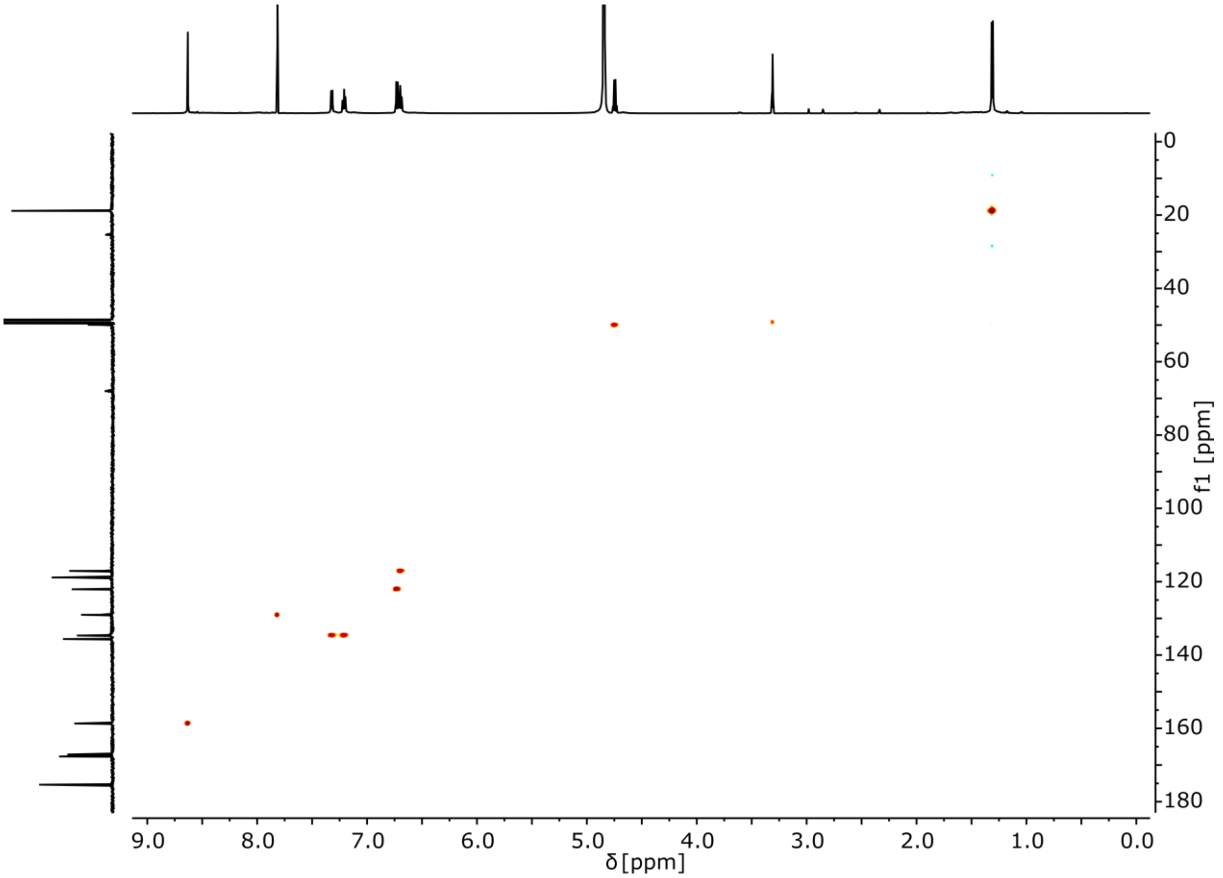

Figure S26. ^1H-^{13}C HSQC spectrum of *S*-**9b** (methanol-d4, 600 MHz, 298 K).

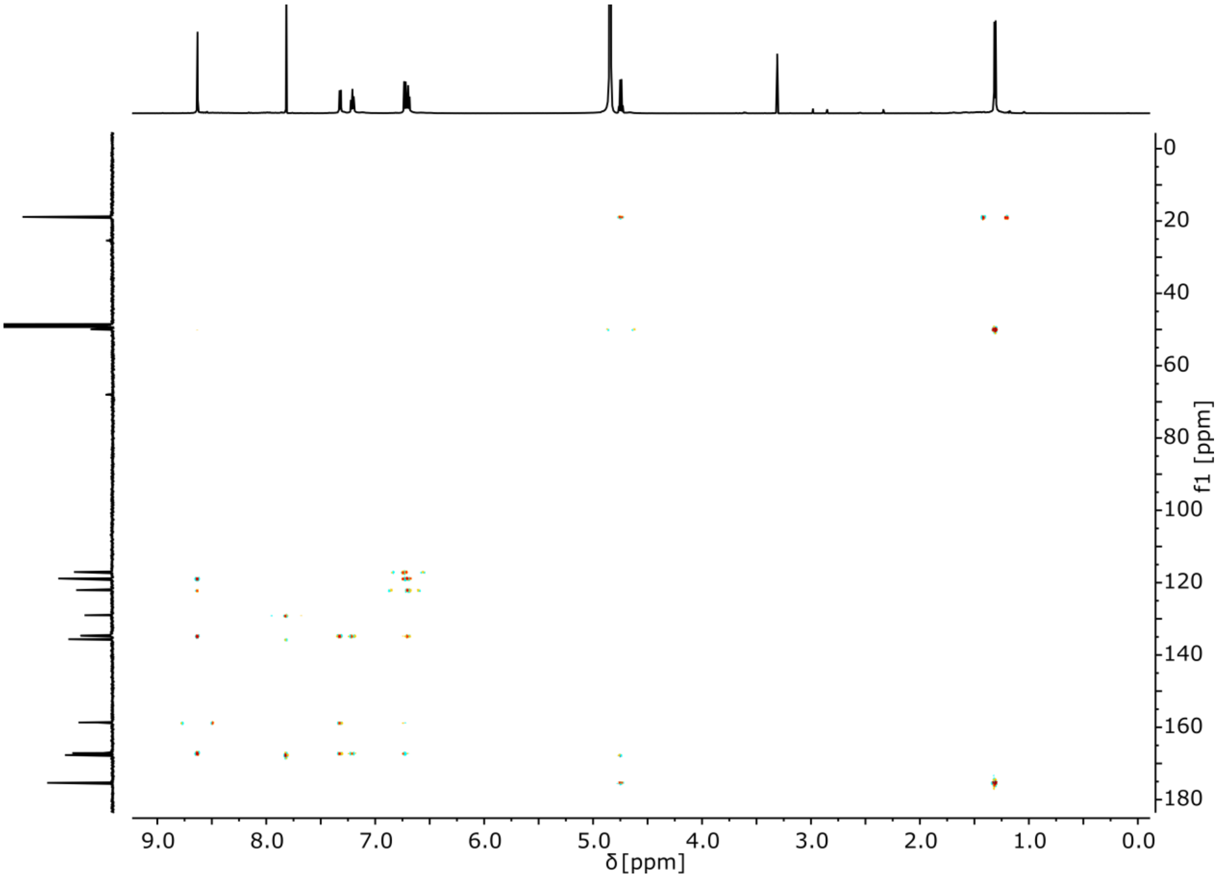

Figure S27. ^1H-^{13}C HMBC spectrum of *S*-**9b** (methanol-d4, 600 MHz, 298 K).

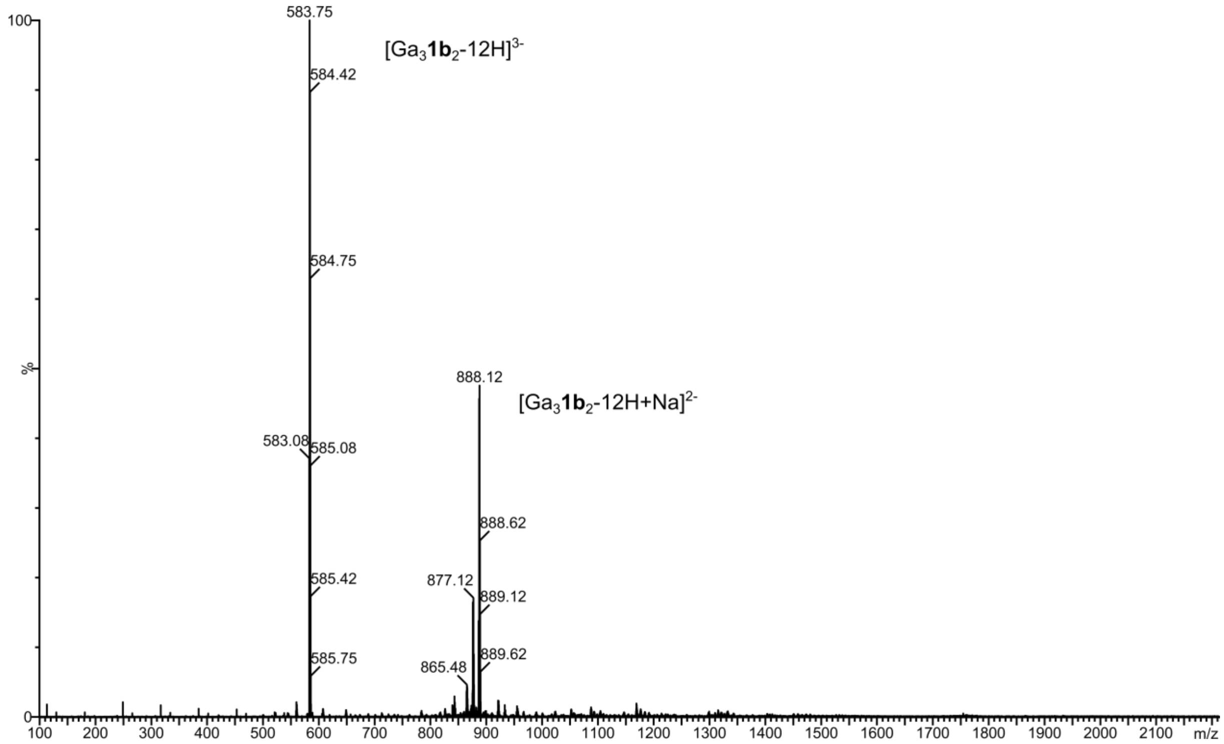

Figure S28. ESI MS spectrum of *S*-**9b**.

Scheme S1. Synthesis of ligands **2a**-**b** and complexes **10a**-**b**.

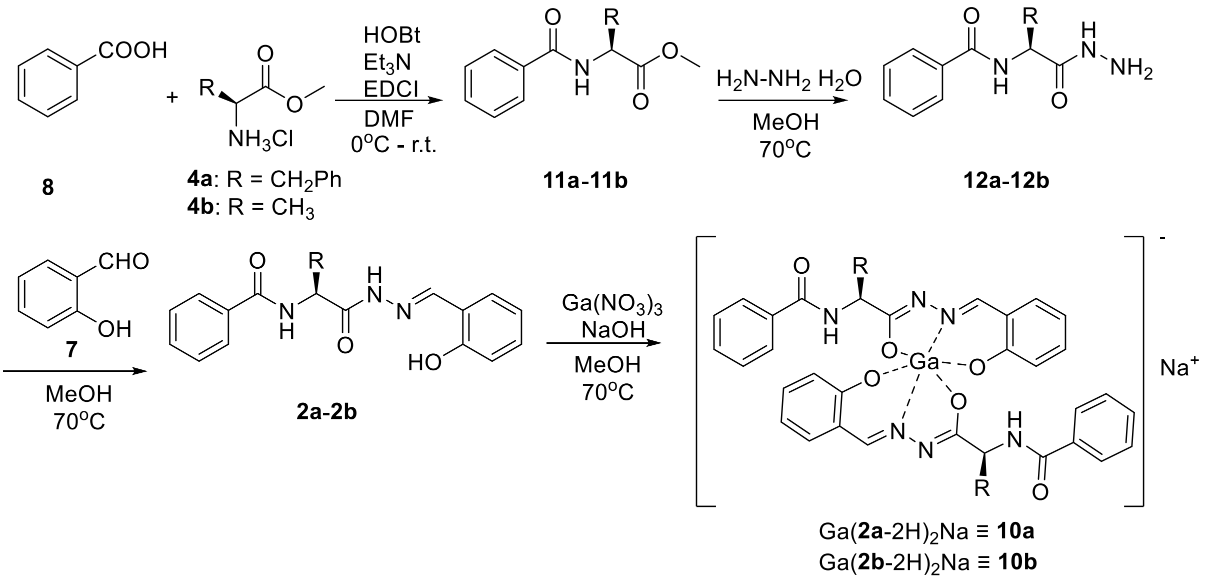

S-**12a**

N-benzoil- *S*-phenylalanine methyl ester **11a** (2 mmol, 0.567 g) was dissolved in methanol (10 ml) and 2 equivalents of hydrazine hydrate were added (4 mmol, 0.2 ml). The reaction mixture was stirred 24 h at 50°C and the evaporated. The solid was washed with $CHCl_3/Et_2O$ 1:2 mixture and dried under vacuum. Yield 0.425 g, 75 %.

^{1}H NMR (400 MHz, dimethyl sulfoxide-d6, 303K): δ= 9.28 (s, 1H), 8.54 (d, *J* = 8.5 Hz, 1H), 7.82-7.74 (m, 2H), 7.54-7.12 (m, 10H), 4.69-4.63 (m, 1H), 4.26 (br s, 2H), 3.07-2.97 ppm (m, 2H). ^{13}C NMR (100 MHz, dimethyl sulfoxide-d6, 303K): δ= 170.6, 166.1, 138.3, 134.1, 131.2, 129.1, 128.07, 128.03, 127.4, 126.2, 53.6, 37.5 ppm.

S-**2a**

S-**12a** (1 mmol, 0.283 g) was dissolved in methanol (5 ml) and 2 equivalents of salicyl aldehyde **7** were added (2 mmol, 0.19 ml). The reaction mixture was heated 24 h at 70°C in a sealed tube and then evaporated. The solid was washed with diethyl ether and dried under reduced pressure. Yield 0.295 g, 76 %. $[\alpha]_D^{25}$= 175.1 (c=1 in DMSO). The product was obtained as a mixture of two diastereoisomers in 2.2:1 ratio. Main diastereoisomer ^{1}H NMR (400 MHz, dimethyl sulfoxide-d6, 303K): δ= 11.88 (s, 1H, *NH*^{1}), 11.07 (s, 1H, *OH*), 8.81 (d, *J* = 8.0 Hz, 1H, *NH*^{2}), 8.46 (s, 1H, *g*), 7.86-7.81 (m, 2H, *c*), 7.56-7.24 (m, 9H, *Ph+j+d+b*), 7.22-7.15 (m, 1H, *k*), 6.95-6.88 (m, 2H, *i+l*), 4.81-4.73 (m, 1H, α), 3.20-3.02 ppm (m, 2H, β). ^{13}C NMR (100 MHz, dimethyl sulfoxide-d6, 303K): δ= 167.8 (*f*), 166.5 (*e*), 157.3 (*h*), 147.5 (*g*), 138.0 (*a*), 133.8 (*Ph*), 131.3 (*d+k*), 129.3 (*j*), 129.1 (*Ph*), 128.15 (*b+Ph*), 127.5 (*c*), 126.35 (*Ph*), 119.3 (*l*), 118.6 (*m*), 116.3 (*i*), 54.2 (α), 36.9 (β). Minor diastereoisomer ^{1}H NMR (400 MHz, dimethyl sulfoxide-d6, 303K): δ= 11.41 (s, 1H, *NH*^{1}), 10.07 (s, 1H, *OH*), 8.67 (d, *J* = 8.3 Hz, 1H, *NH*^{2}), 8.36 (s, 1H, *g*), 7.86-7.81 (m, 2H, *c*), 7.76-7.72 (m, 1H, *j*), 7.56-7.24 (m, 8H, *Ph+d+b*), 7.22-7.15 (m, 1H, *k*), 6.95-6.88 (m, 2H, *i+l*), 5.58-5.51 (m, 1H, α), 3.20-3.02 ppm (m, 2H, β). ^{13}C NMR (100 MHz, dimethyl sulfoxide-d6, 303K): δ= 172.4 (*f*), 166.3 (*e*), 156.4 (*h*), 141.2 (*g*), 138.4 (*a*), 134.0 (*Ph*), 131.2 (*d*), 131.1 (*k*), 129.0 (*Ph*), 128.12 (*b+Ph*), 127.4 (*c*), 126.31 (*j*), 126.1 (*Ph*), 120.2 (*m*), 119.4 (*l*), 116.2 (*i*), 52.0 (α), 36.1 (β). HRMS (EI) *m/z* calcd for $C_{23}H_{21}N_3O_3$: 387.1583 $[M]^+$, found: 387.1591. IR (KBr, cm^{-1}): 3265, 3059, 3029, 2973, 2925, 2868, 1675, 1641, 1577, 1538, 1487, 1439, 1415, 1351, 1334, 1270, 1240, 1214, 1199, 1153, 1083, 1033, 958, 929, 876, 848, 792, 753, 698, 656, 597, 567, 548, 516, 497, 473, 441. Analysis calcd for $C_{23}H_{21}N_3O_3$: C 71.30, H 5.46, N 10.85, found: C 71.18, H 5.42, N 10.80.

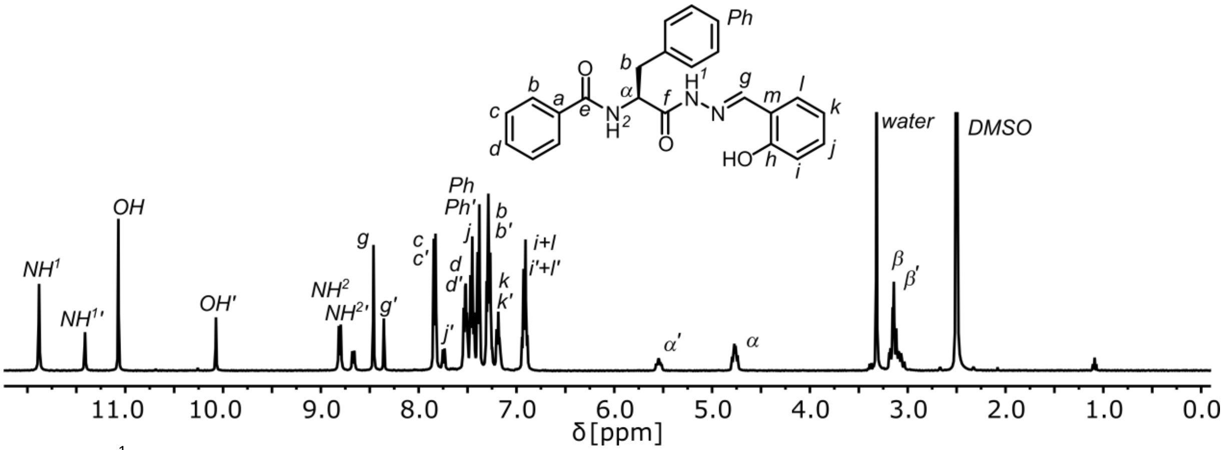

Figure S29. ^{1}H NMR spectrum of *S*-**2a** (dimethyl sulfoxide-d6, 400 MHz, 303 K).

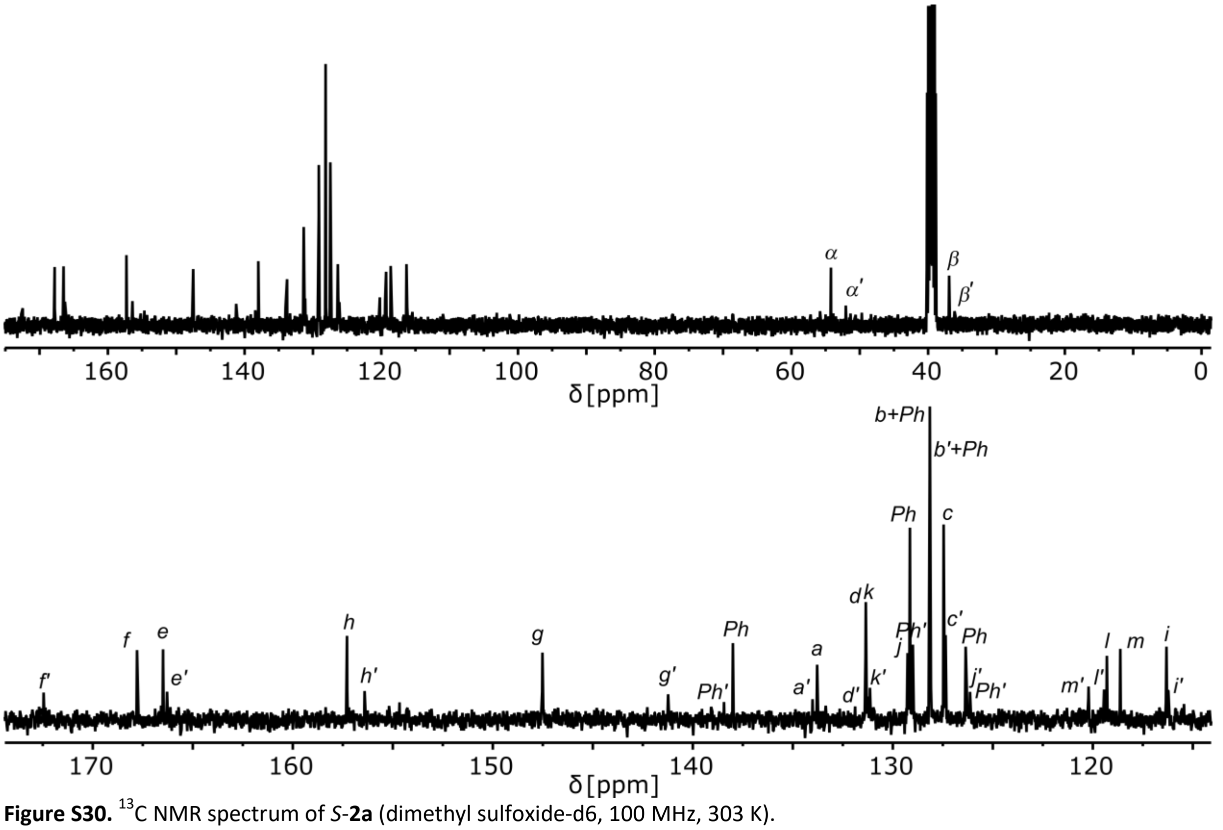

Figure S30. ^{13}C NMR spectrum of *S*-**2a** (dimethyl sulfoxide-d6, 100 MHz, 303 K).

S-**10a**

S-**2a** (0.02 mmol, 7.7 mg, 2eq.), $Ga(NO_3)_3 \cdot H_2O$ (0.01 mmol, 2.7 mg, 1 eq.) and NaOH (0.04 mmol, 1.6 mg, 4eq.) were dissolved in methanol (0.7 mL) and heated at 70°C in a sealed tube overnight. The complex was obtained as a mixture of diastereoisomers. ^{1}H NMR (400 MHz, methanol-d4, 303K): δ= 8.53 (s), 8.526 (s), 8.523 (s), 8.51 (s), 8.34 (s), 7.77-7.70 (m), 7.66-7.63 (m), 7.60-7.04 (m), 6.81-6.63 (m), 5.00-4.87 (m), 3.25-3.12 (m), 3.10-3.00 (m), 2.95-2.87 (m). HRMS (APCI) *m/z* calcd for $C_{46}H_{38}N_6O_6Ga$: 839.2109 $[M]^-$, found 839.2104. IR (KBr, cm^{-1}): 3422, 3060, 3027, 2427, 1623, 1578, 1530, 1485, 1471, 1446, 1384, 1288, 1199, 1151, 1125, 1077, 1031, 969, 902, 849, 795, 756, 701, 660, 585, 510, 417. Analysis calcd for $C_{46}H_{38}N_6O_6GaNa \cdot 2NaNO_3 \cdot 4H_2O$: C 49.97, H 4.19, N 10.14, found: C 50.12, H 3.97, N 10.06.

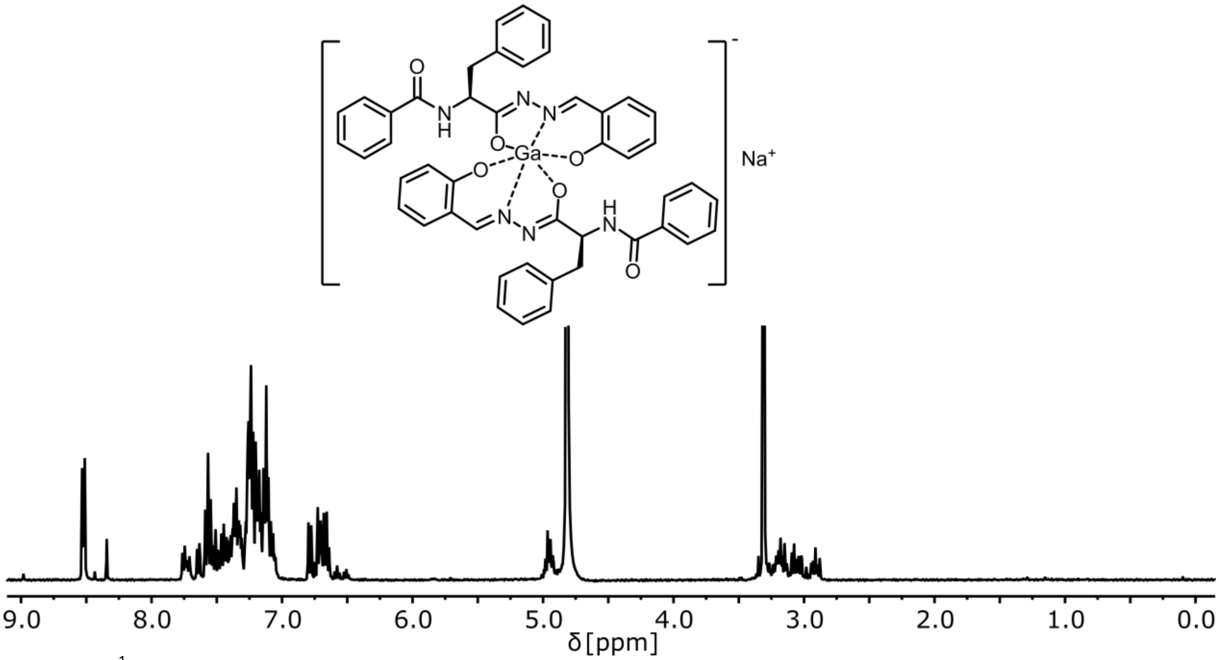

Figure S31. ^{1}H NMR spectrum of *S*-**10a** (methanol-d4, 400 MHz, 303 K).

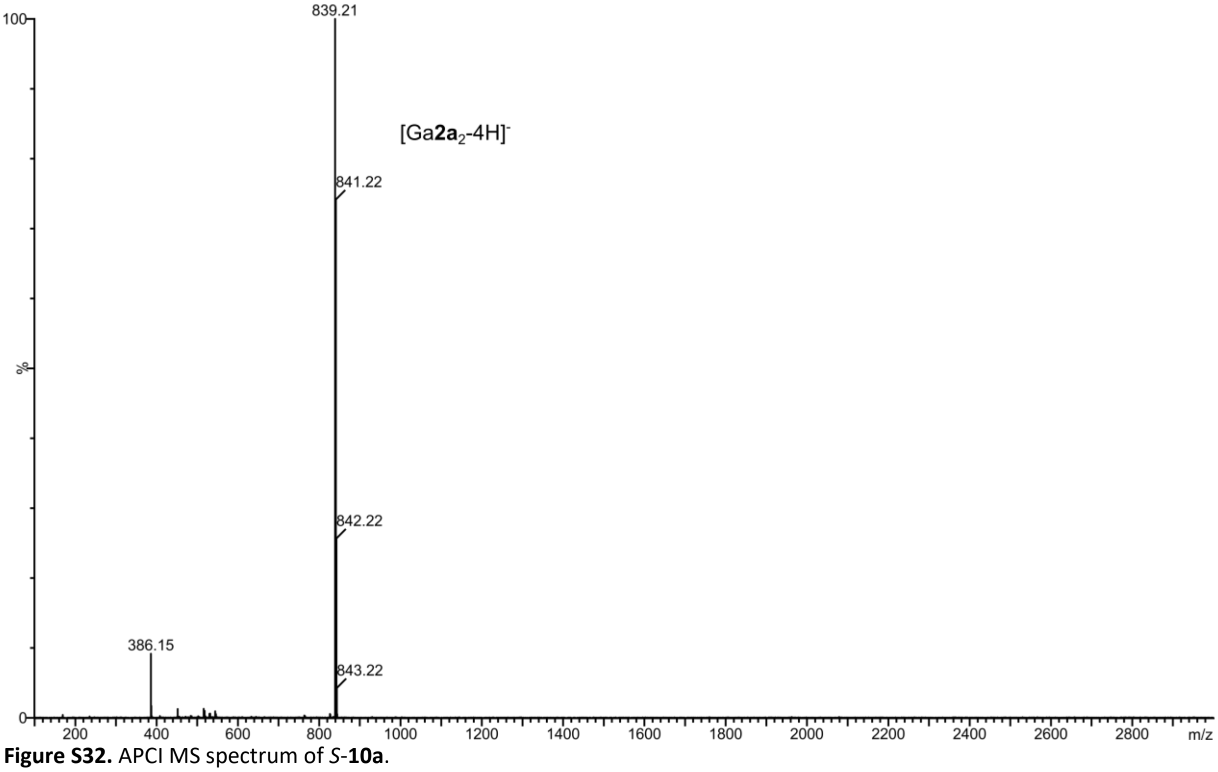

Figure S32. APCI MS spectrum of *S*-**10a**.

S-**12b**

N-benzoil-*S*-alanine methyl ester **11b** (2 mmol, 0.41 g) was dissolved in methanol (10 ml) and 2 equivalents of hydrazine hydrate were added (4 mmol, 0.2 ml). The reaction mixture was stirred 24 h at 50°C and the evaporated. The solid was washed with $CHCl_3/Et_2O$ 1:2 mixture and dried under vacuum. Data in agreement with literature.[1]

S-**2b**

S-**12b** (1 mmol, 0.207 g) was dissolved in methanol (5 ml) and 2 equivalents of salicyl aldehyde **7** were added (2 mmol, 0.19 ml). The reaction mixture was heated 24 h at 70°C in a sealed tube and then evaporated. The solid was washed with diethyl ether and dried under reduced pressure. Yield

60 %. $[\alpha]_D^{25}$= 176.8 (c=1 in DMSO). The product was obtained as a mixture of two diastereoisomers in 2.5:1 ratio. Major diastereoisomer ^{1}H NMR (600 MHz, dimethyl sulfoxide-d6, 298K): δ= 11.78 (s, 1H, *NH¹*), 11.13 (s, 1H, *OH*), 8.71 (d, *J* = 7.0 Hz, 1H, *NH²*), 8.46 (s, 1H, *g*), 7.94-7.91 (m, 2H, *c*), 7.57-7.52 (m, 1H, *d*), 7.52-7.50 (m, 1H, *j*), 7.50-7.45 (m, 2H, *b*), 7.30-7.26 (m, 1H, *k*), 6.93-6.89 (m, 2H, *i+l*), 4.53 (dq, J_1 = 7.0 Hz, J_2 = 7.1 Hz, 1H, α), 1.42 ppm (d, *J* = 7.1 Hz, 3H, β). ^{13}C NMR (150 MHz, dimethyl sulfoxide-d6, 298K): δ= 168.9 (*f*), 166.3 (*e*), 157.3 (*h*), 147.4 (*g*), 133.8 (*a*), 131.4 (*d*), 131.32 (*k*), 129.4 (*j*), 128.2 (*b*), 127.6 (*c*), 119.3 (*l*), 118.7 (*m*), 116.4 (*i*), 48.3 (α), 17.5 ppm (β). Minor diastereoisomer ^{1}H NMR (600 MHz, dimethyl sulfoxide-d6, 298K): δ= 11.34 (s, 1H, *NH¹*), 10.08 (s, 1H, *OH*), 8.69 (d, *J* = 7.4 Hz, 1H, *NH²*), 8.32 (s, 1H, *g*), 7.92-7.89 (m, 2H, *c*), 7.70-7.68 (m, 1H, *j*), 7.57-7.52 (m, 1H, *d*), 7.50-7.45 (m, 2H, *b*), 7.26-7.22 (m, 1H, *k*), 6.89-6.85 (m, 2H, *i+l*), 5.27 (dq, J_1 = 7.4 Hz, J_2 = 7.2 Hz, 1H, α), 1.43 ppm (d, *J* = 7.2 Hz, 3H, β). ^{13}C NMR (150 MHz, dimethyl sulfoxide-d6, 298K): δ= 173.5 (*f*), 166.0 (*e*), 156.4 (*h*), 140.9 (*g*), 134.1 (*a*), 131.29 (*d*), 131.1 (*k*), 128.2 (*b*), 127.5 (*c*), 126.3 (*j*), 120.2 (*m*), 119.5 (*l*), 116.2 (*i*), 46.1 (α), 16.6 ppm (β). HRMS (EI) *m/z* calcd for $C_{17}H_{17}N_3O_3$: 311.1270 $[M]^+$, found: 311.1273. IR (KBr, cm^{-1}): 3280, 3191, 3061, 2979, 2868, 1675, 1636, 1577, 1531, 1487, 1448, 1408, 1370, 1342, 1297, 1274, 1217, 1200, 1153, 1123, 1098, 1033, 957, 930, 907, 892, 800, 754, 715, 693, 649, 583, 548, 474, 444, 428. Analysis calcd for $C_{17}H_{17}N_3O_3$: C 65.58, H 5.50, N 13.50, found: C 65.41, H 5.54, N 13.44.

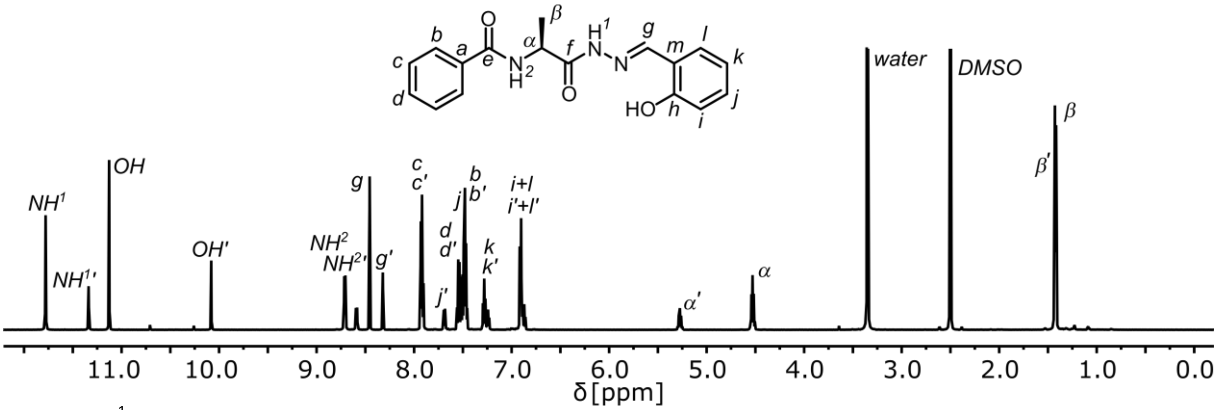

Figure S33. ^{1}H NMR spectrum of *S*-**2b** (dimethyl sulfoxide-d6, 600 MHz, 298 K).

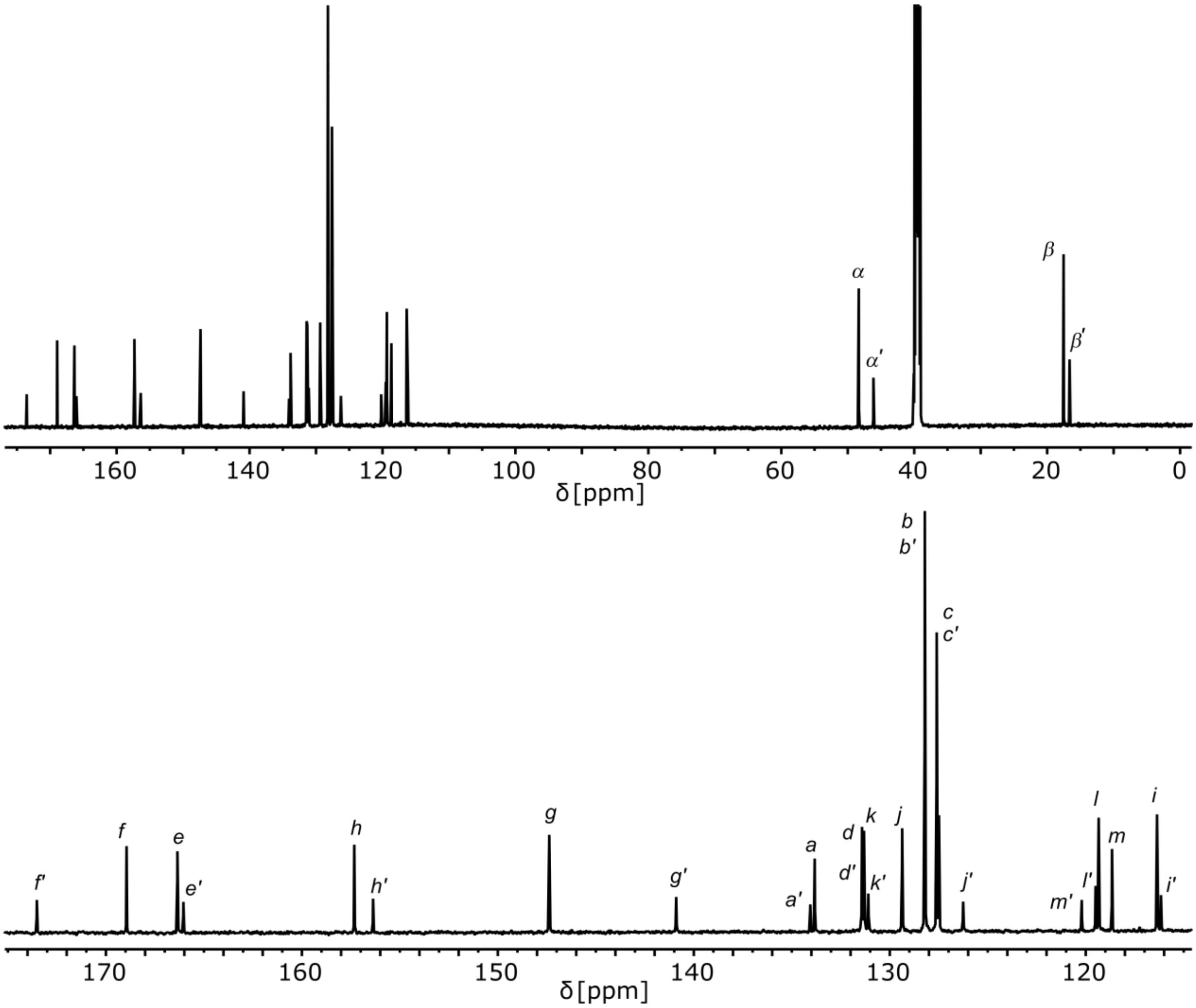

Figure S34. 13C NMR spectrum of *S*-**2a** (dimethyl sulfoxide-d6, 150 MHz, 298 K).

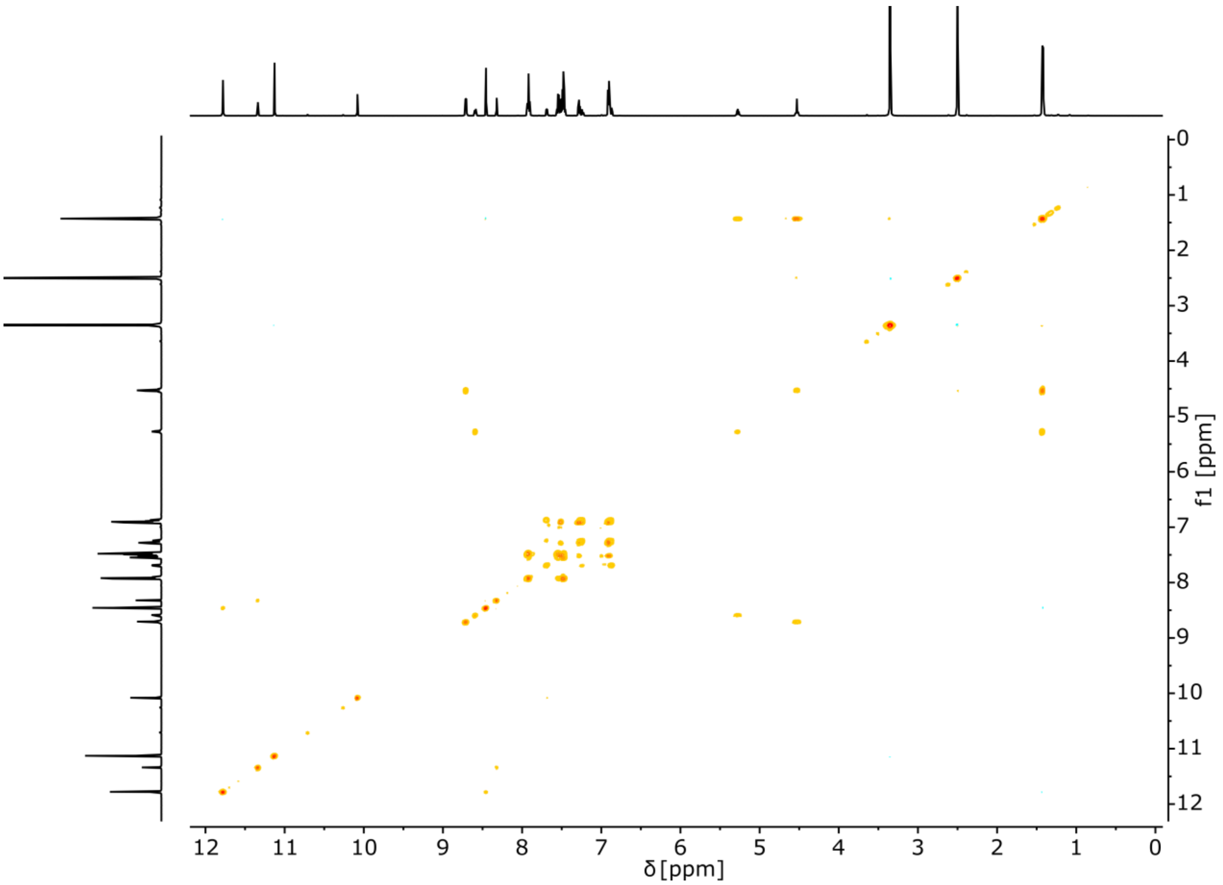

Figure S35. ^{1}H-^{1}H COSY spectrum of *S*-**2b** (dimethyl sulfoxide-d6, 600 MHz, 298 K).

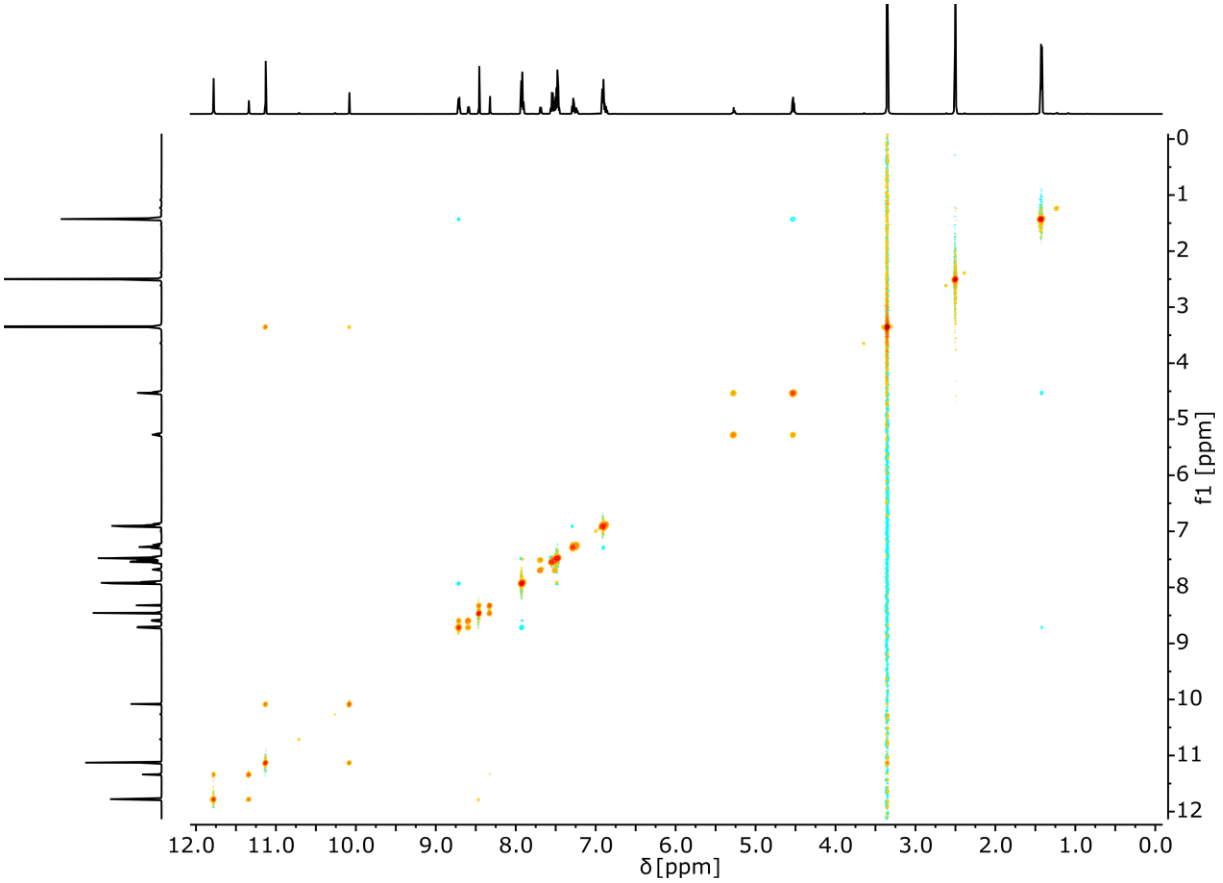

Figure S36. ^{1}H-^{1}H NOESY spectrum of *S*-**2b** (dimethyl sulfoxide-d6, 600 MHz, 298 K).

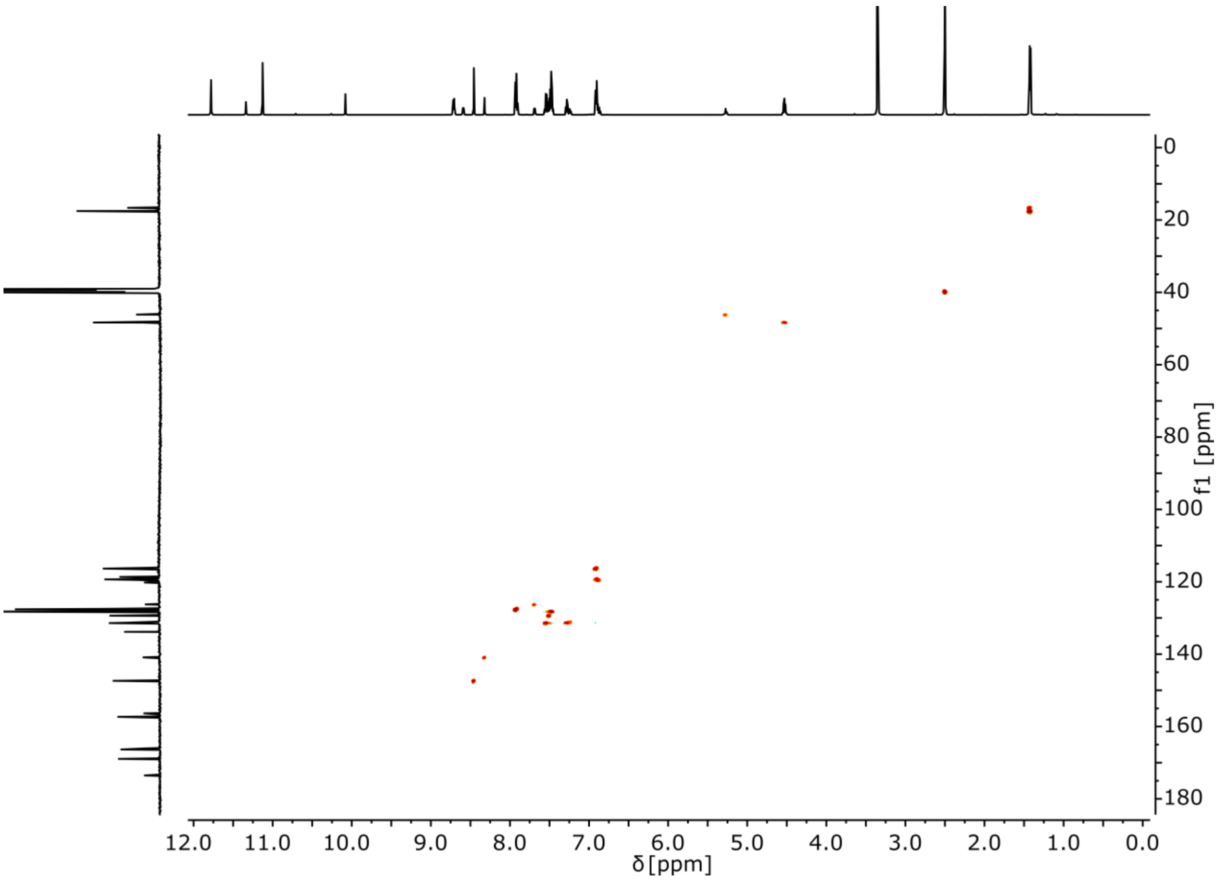

Figure S37. ^{1}H-^{13}C HSQC spectrum of *S*-**2b** (dimethyl sulfoxide-d6, 600 MHz, 298 K).

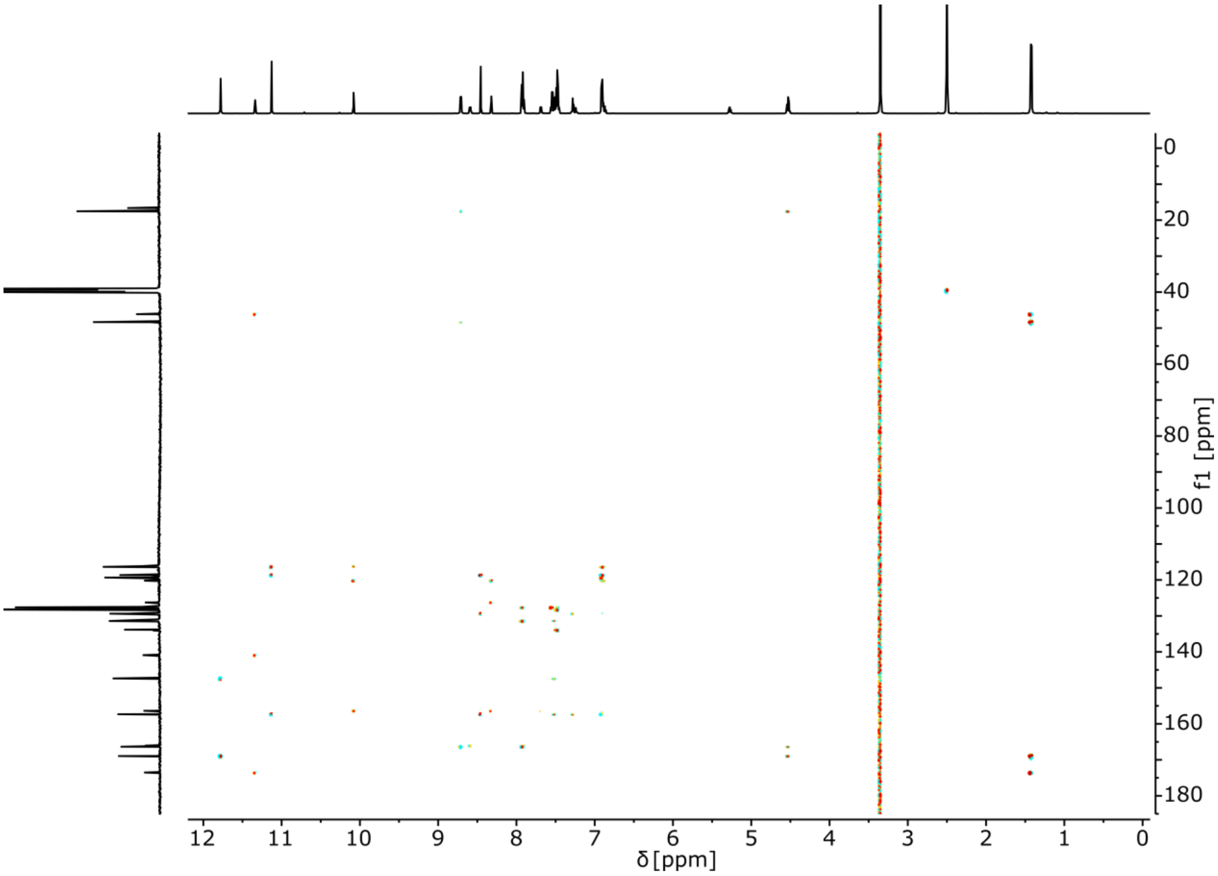

Figure S38. ^{1}H-^{13}C HMBC spectrum of *S*-**2b** (dimethyl sulfoxide-d6, 600 MHz, 298 K).

S-10b

S-**2b** (0.02 mmol, 6.2 mg, 2eq.), $Ga(NO_3)_3 \cdot H_2O$ (0.01 mmol, 2.7 mg, 1 eq.) and NaOH (0.04 mmol, 1.6 mg, 4eq.) were dissolved in methanol (0.7 mL) and heated at 70°C in a sealed tube overnight. The complex was obtained as a mixture of diastereoisomers in 45:55 ratio. Major diastereoisomer ^{1}H NMR (600 MHz, methanol-d4, 298K): δ= 8.55 (s, 2H, *g*), 7.69-7.66 (m, 4H, *b*), 7.49-7.45 (m, 2H, *d*), 7.40-7.35 (m, 4H, *c*), 7.28-7.25 (m, 2H, *l*), 7.15-7.11 (m, 2H, *j*), 6.71-6.64 (m, 4H, *i*+*k*), 4.77-4.71 (m, 2H, α), 1.41 (d, *J* = 7.0 Hz, 6H, β). ^{13}C NMR (150 MHz, methanol-d4, 298K): δ= 175.14 (*f*), 169.2 (*e*), 167.1 (*h*), 158.5 (*g*), 135.7 (*a*), 134.57 (*l*), 134.45 (*j*), 132.5 (*d*), 129.46 (*c*), 128.29 (*b*), 122.2 (*i*), 119.2 (*m*), 117.1 (*k*), 49.9(α), 19.5 (β). Minor diastereoisomer ^{1}H NMR (600 MHz, methanol-d4, 298K): δ= 8.56 (s, 2H, *g*), 7.76-7.73 (m, 4H, *b*), 7.49-7.45 (m, 2H, *d*), 7.40-7.35 (m, 4H, *c*), 7.28-7.25 (m, 2H, *l*), 7.20-7.16 (m, 2H, *j*), 6.71-6.64 (m, 4H, *i*+*k*), 4.77-4.71 (m, 2H, α), 1.35 (d, *J* = 7.0 Hz, 6H, β). ^{13}C NMR (150 MHz, methanol-d4, 298K): δ= 175.11 (*f*), 169.1 (*e*), 167.0 (*h*), 158.6 (*g*), 135.6 (*a*), 134.63 (*l*), 134.41 (*j*), 132.6 (*d*), 129.45 (*c*), 128.31 (*b*), 122.1 (*i*), 119.1 (*m*), 117.0 (*k*), 49.7 (α), 19.6 (β). HRMS (APCI) *m/z* calcd for $C_{34}H_{30}N_6O_6Ga$: 687.1483 $[M]^-$, found 687.1480. IR (KBr, cm^{-1}): 3410, 3060, 3028, 2981, 2935, 2428, 1789, 1624, 1602, 1578, 1529, 1486, 1471, 1446, 1366, 1292, 1200, 1152, 1123, 1075, 1034, 970, 901, 836, 797, 758, 714, 660, 585, 481. Analysis calcd for $C_{34}H_{30}N_6O_6GaNa \cdot 2NaNO_3 \cdot 3H_2O$: C 43.66, H 3.88, N 11.98, found: C 43.66, H 3.67, N 11.88.

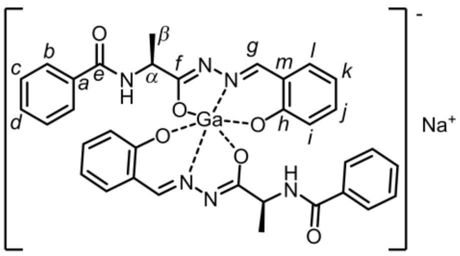

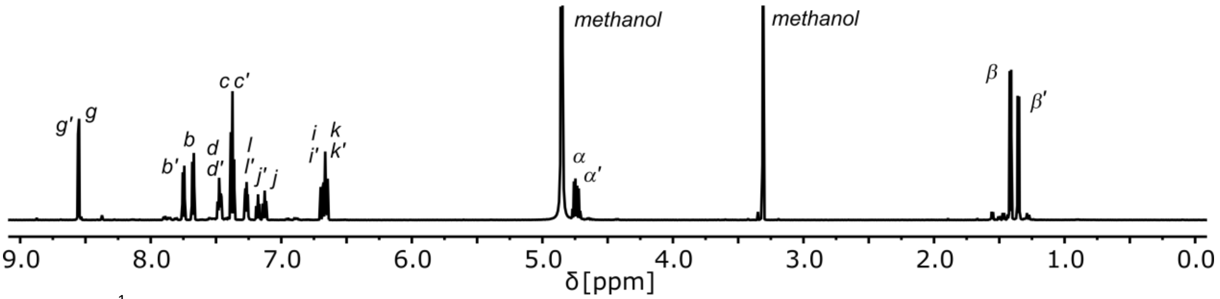

Figure S39. ^{1}H NMR spectrum of S-**10b** (methanol-d4, 600 MHz, 298 K).

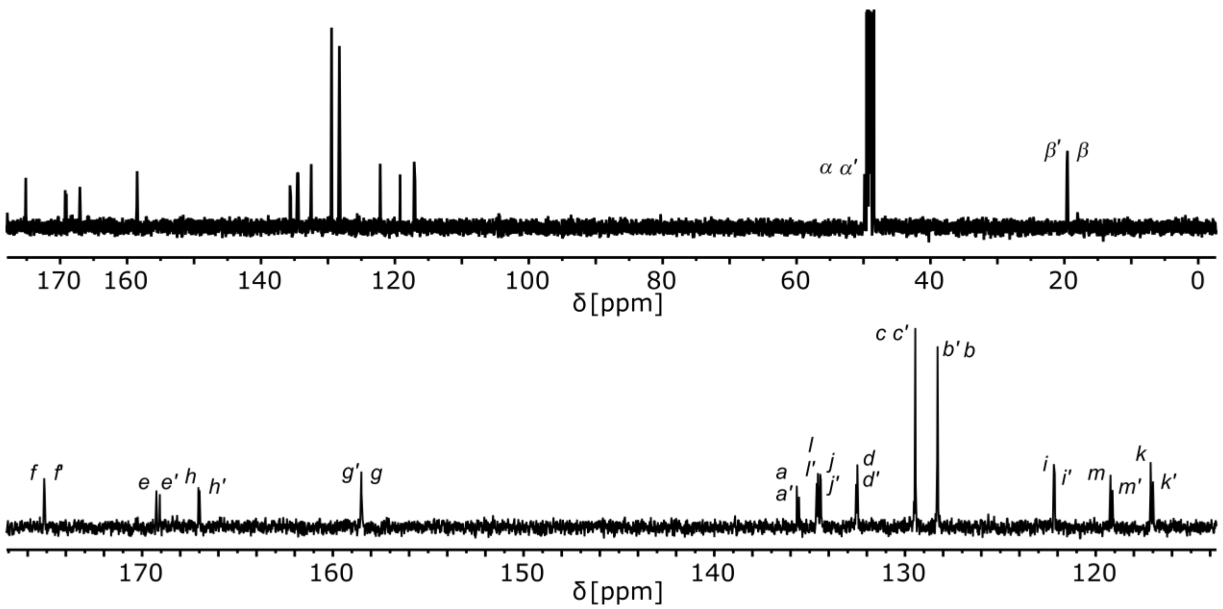

Figure S40. ^{13}C NMR spectrum of *S*-**10b** (methanol-d4, 150 MHz, 298 K).

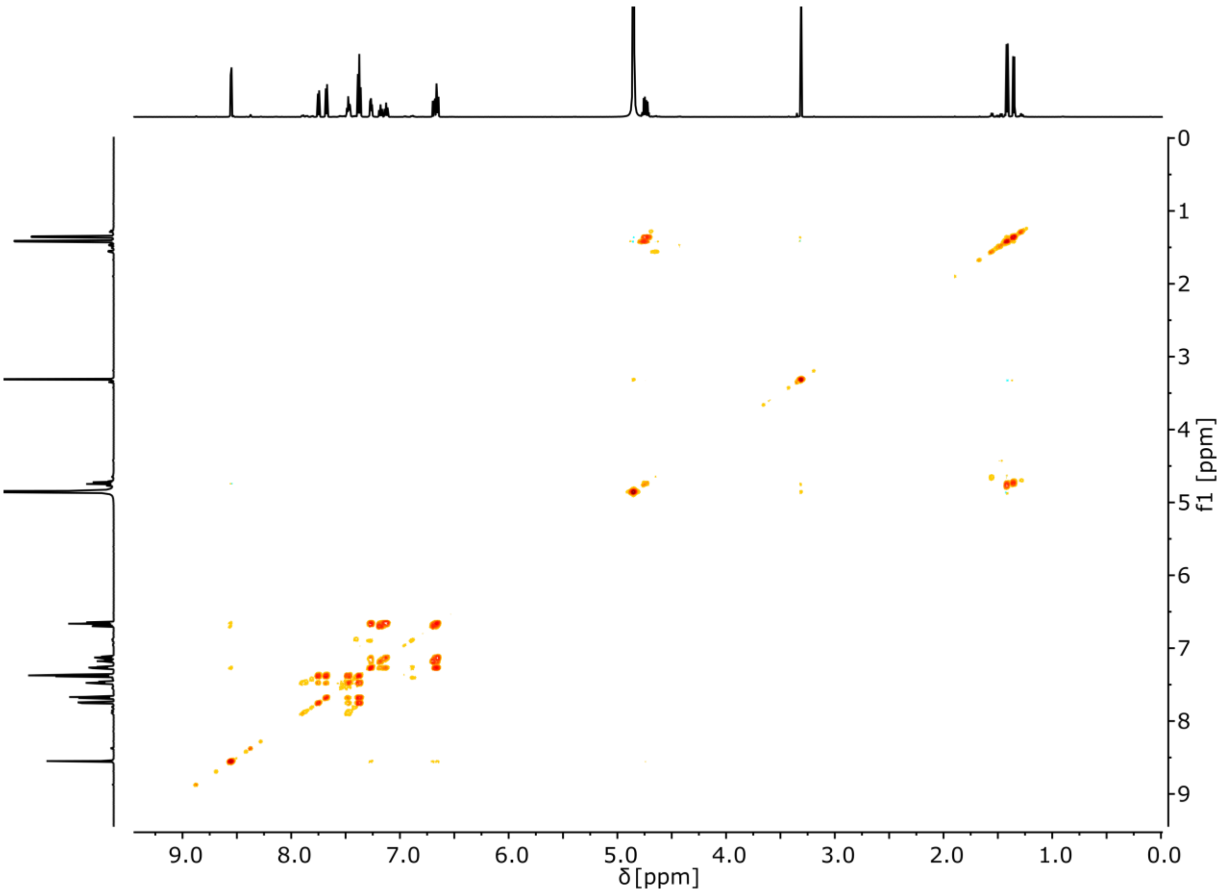

Figure S41. ^{1}H-^{1}H COSY spectrum of *S*-**10b** (methanol-d4, 600 MHz, 298 K).

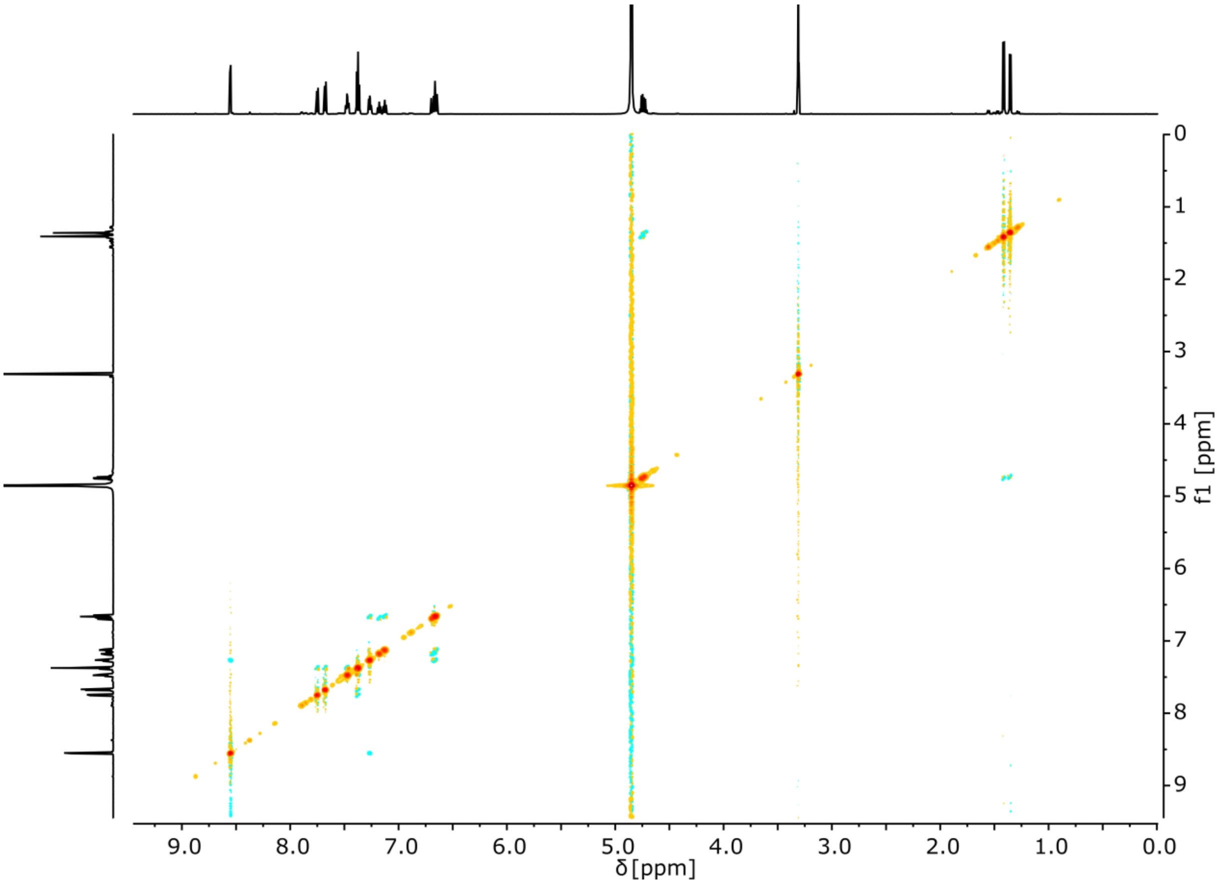

Figure S42. ^1H-^1H NOESY spectrum of *S*-**10b** (methanol-d4, 600 MHz, 298 K).

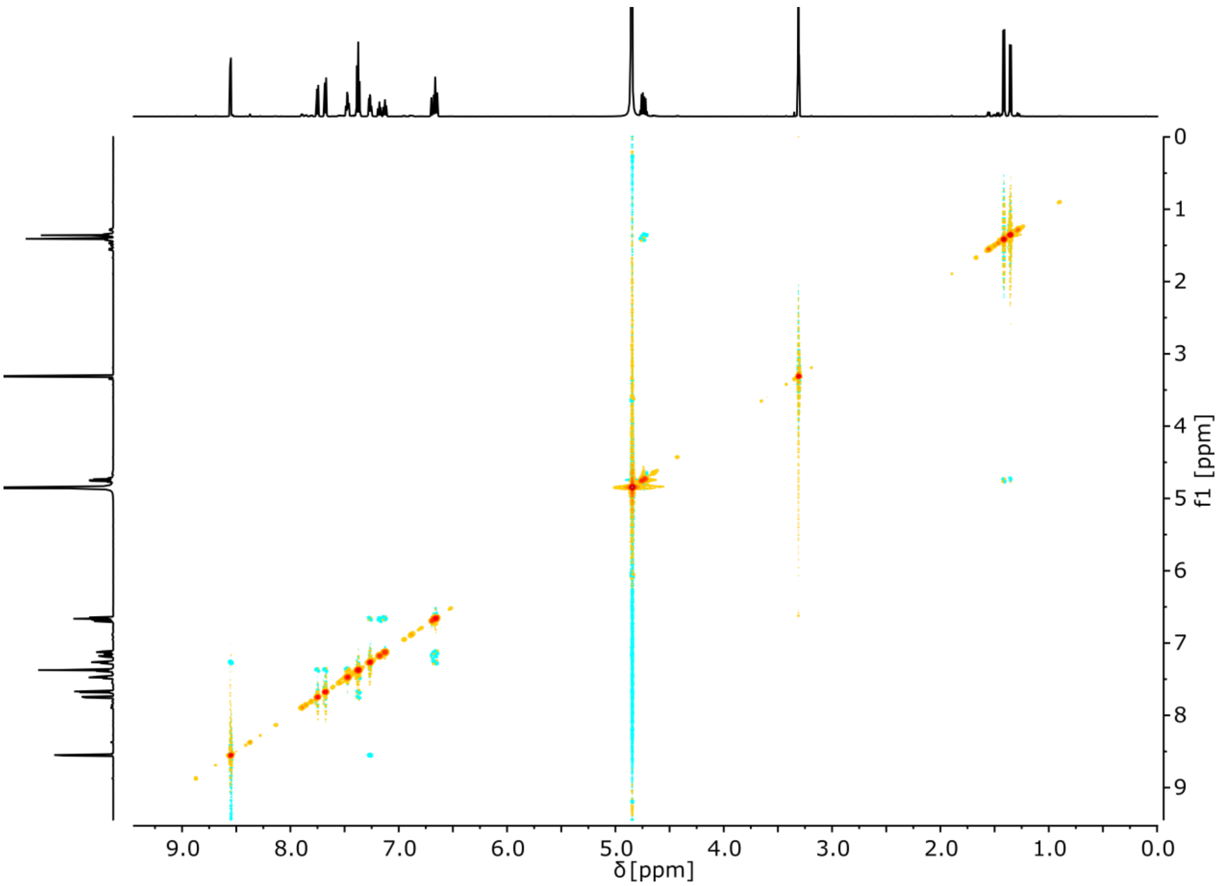

Figure S43. ^1H-^1H ROESY spectrum of *S*-**10b** (methanol-d4, 600 MHz, 298 K).

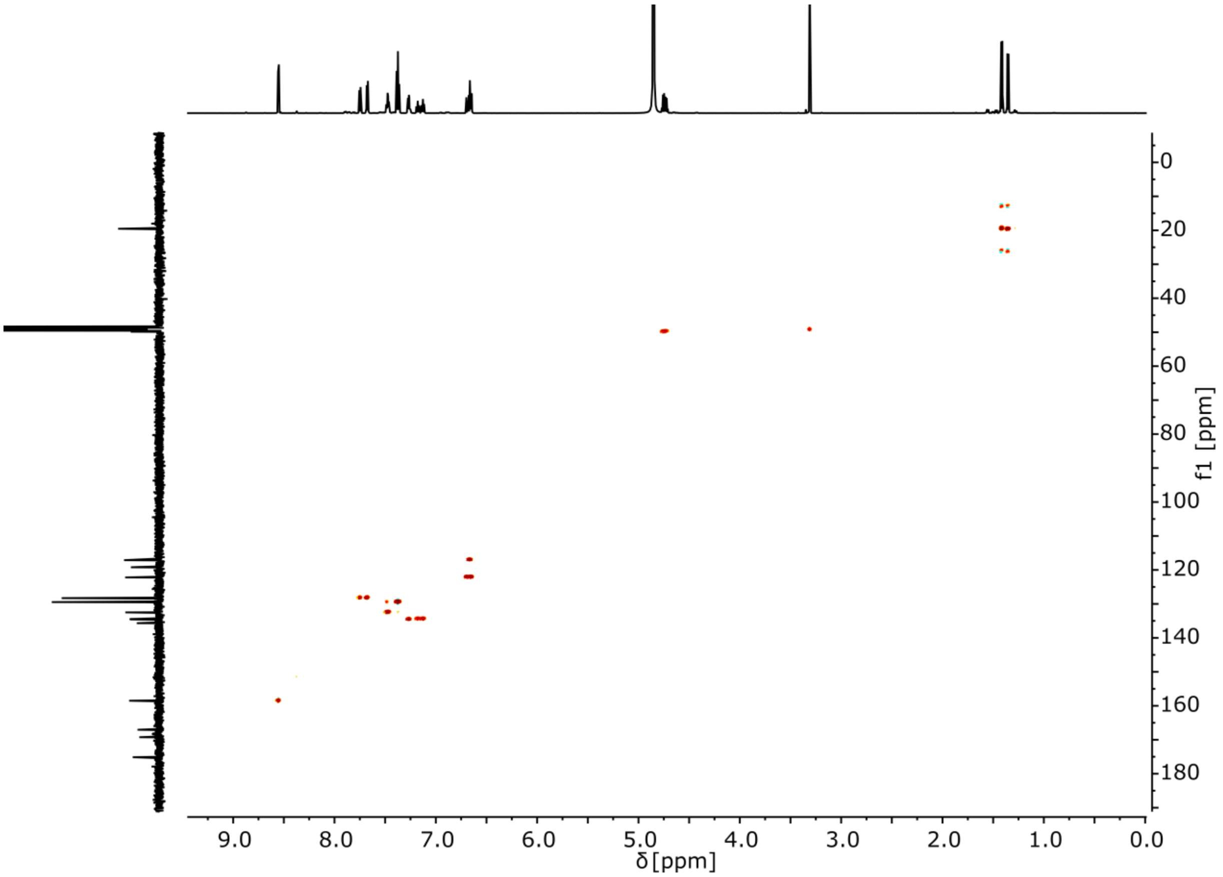

Figure S44. ^{1}H-^{13}C HSQC spectrum of *S*-**10b** (methanol-d4, 600 MHz, 298 K).

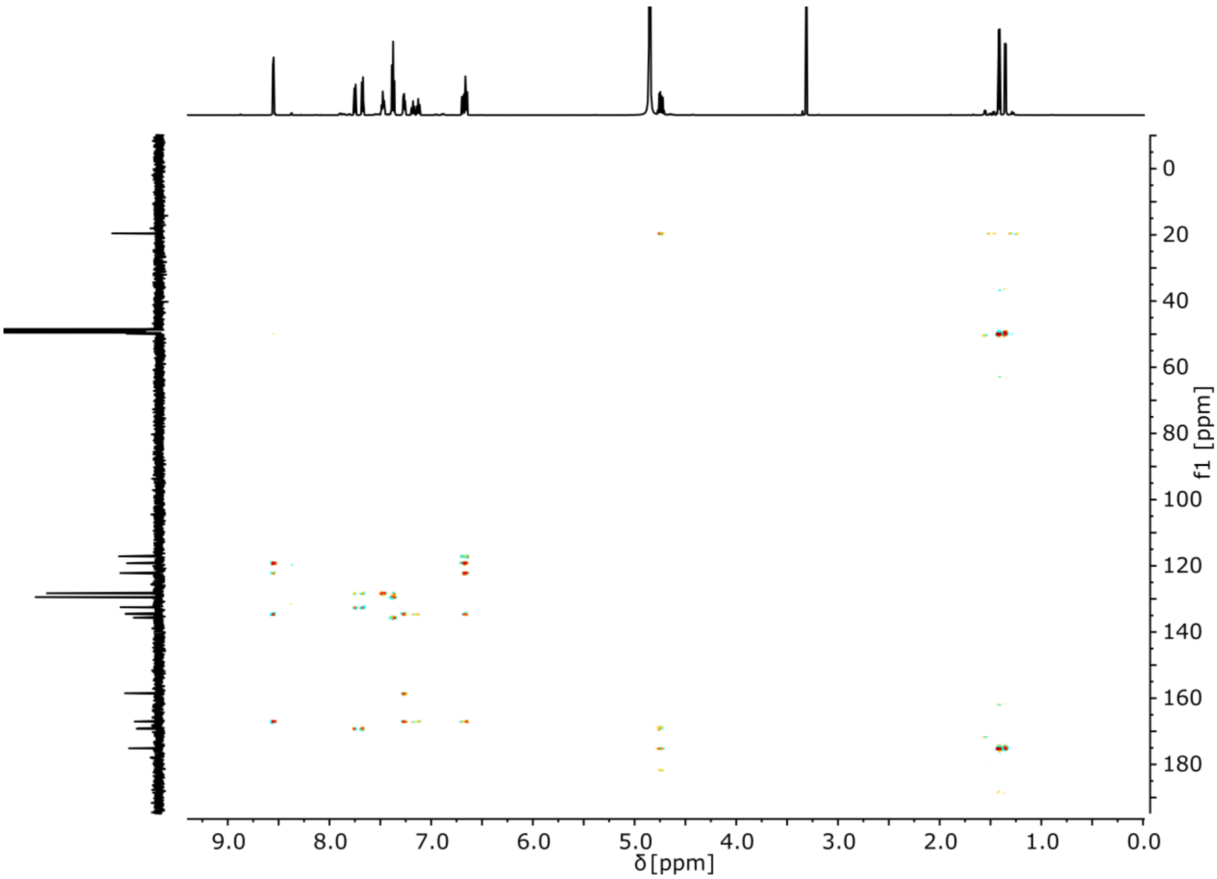

Figure S45. ^{1}H-^{13}C HMBC spectrum of *S*-**10b** (methanol-d4, 600 MHz, 298 K).

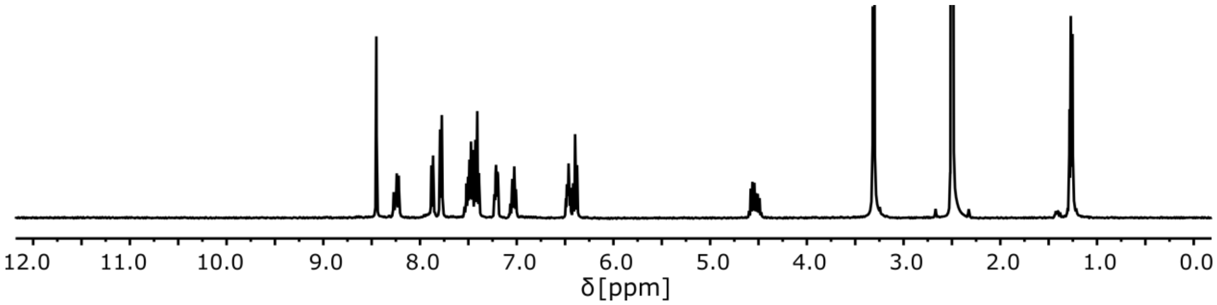

Figure S46. ^{1}H NMR spectrum of *S*-**10b** (dimethyl sulfoxide-d6, 400 MHz, 303 K).

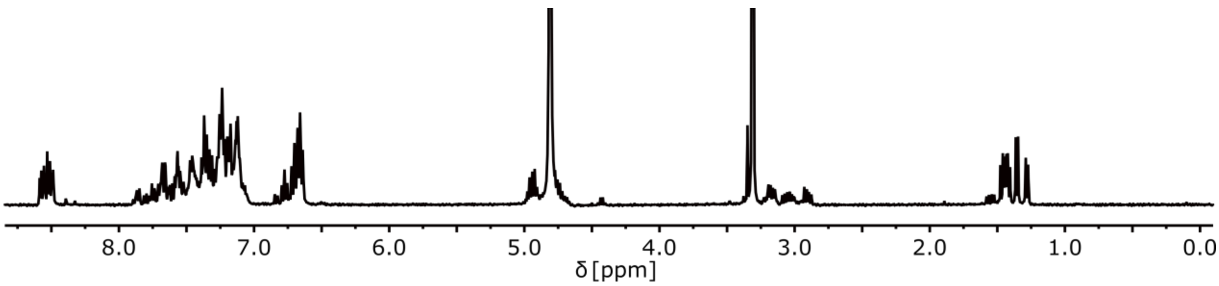

Figure S47. ^{1}H NMR spectrum of mixture of *S*-**10a** and *S*-**10b** (methanol-d4, 400 MHz, 303 K).

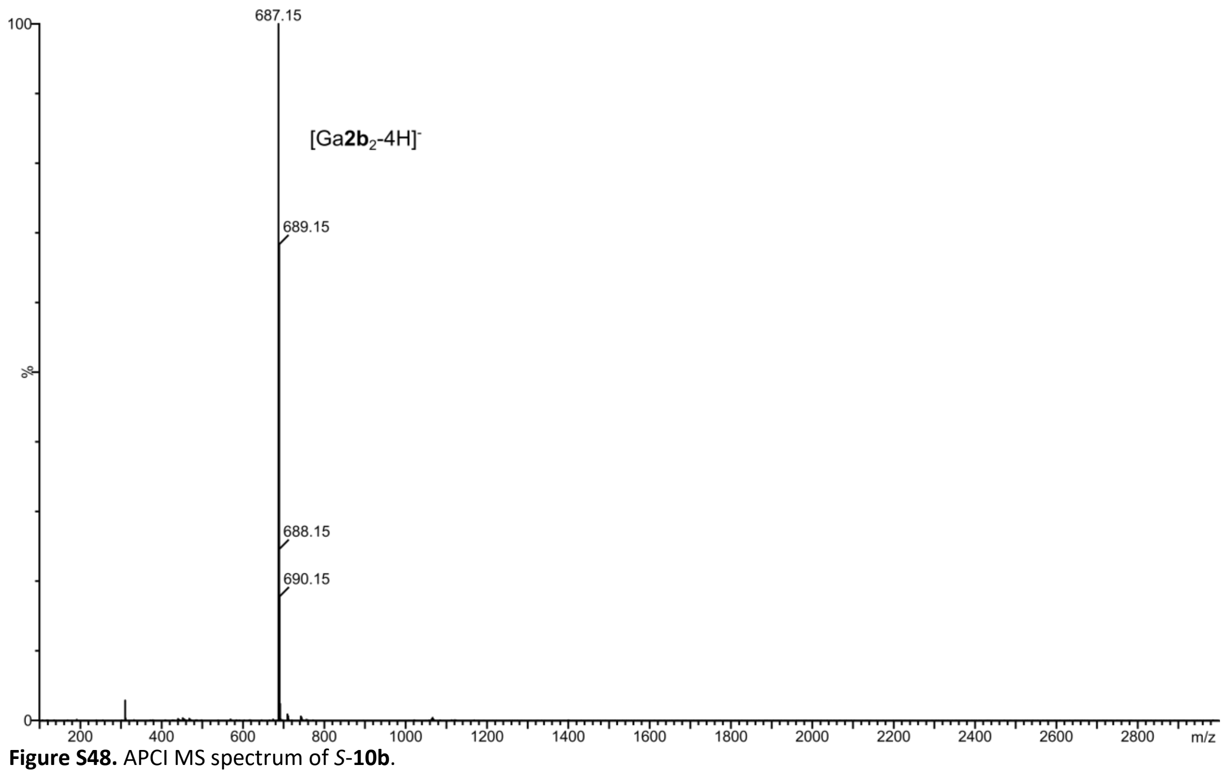

Figure S48. APCI MS spectrum of *S*-**10b**.

ECD and UV spectra

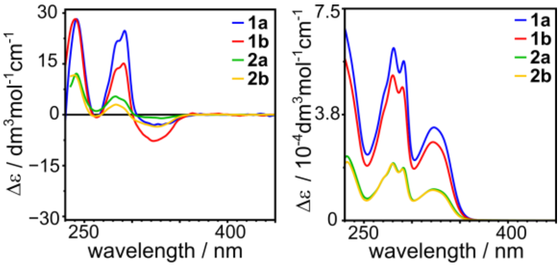

Figure S49. ECD and UV spectra of ligands in THF.

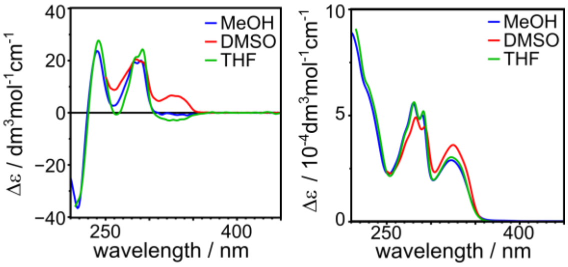

Figure S50. ECD and UV spectra of *S*-**1a**.

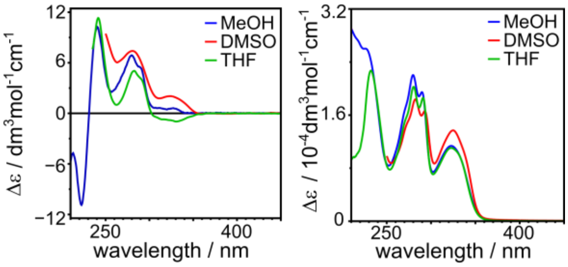

Figure S51. ECD and UV spectra of *S*-**2a**.

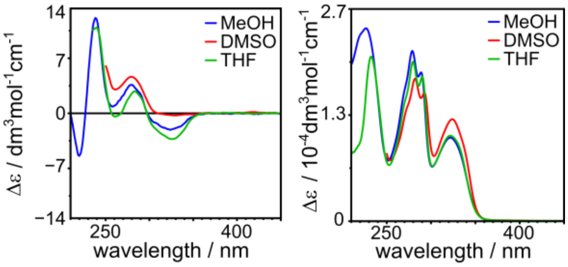

Figure S52. ECD and UV spectra of *S*-**2b**.

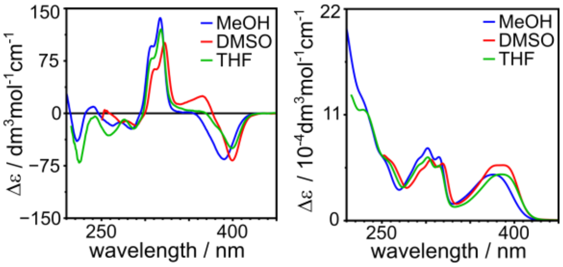

Figure S53. ECD and UV spectra of *S*-**9a**.

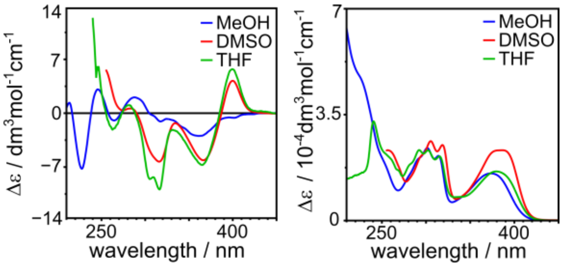

Figure S54. ECD and UV spectra of *S*-**10a**.

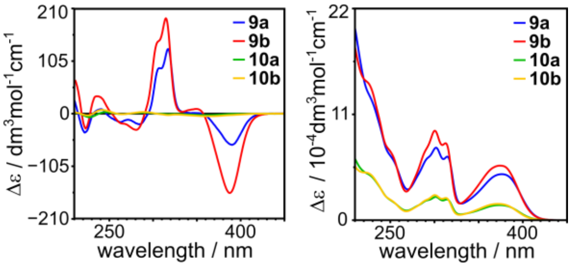

Figure S55. ECD and UV spectra of complexes **9** and **10** in methanol.

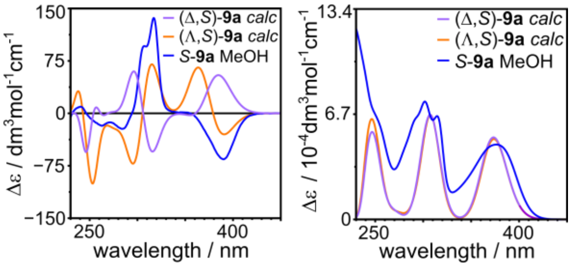

Figure S56. Calculated ECD and UV spectra of *S*-**9a** in methanol.

IR spectra

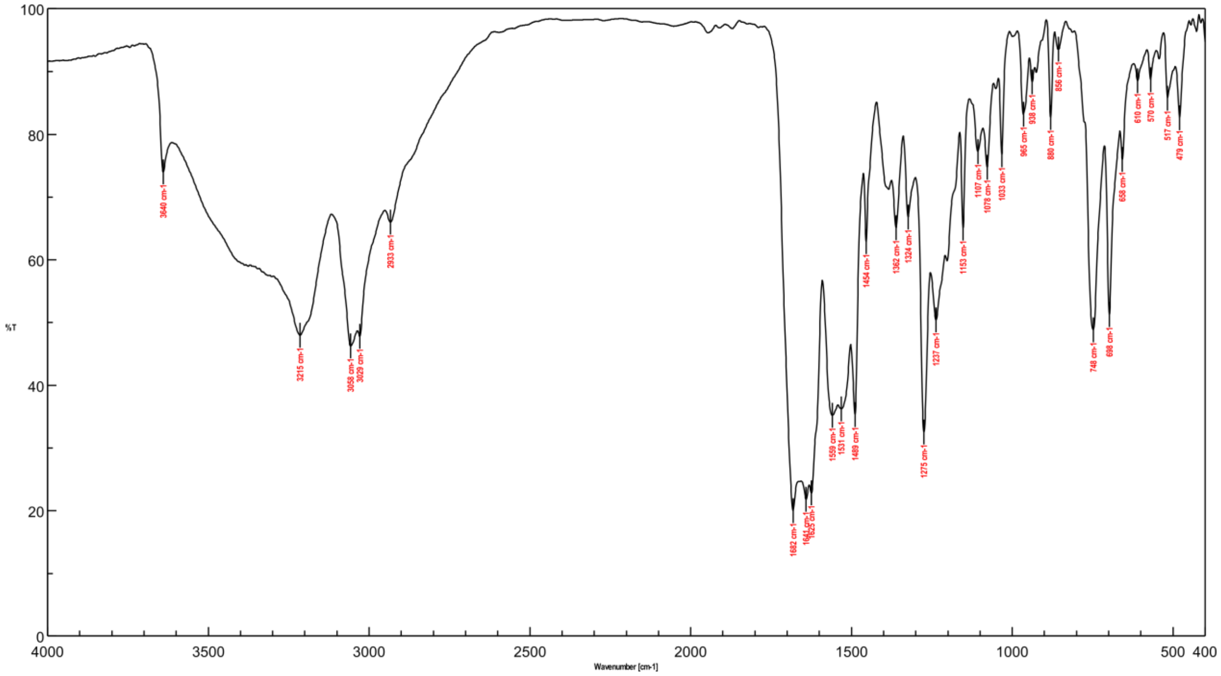

Figure S57. IR spectrum of **1a** (KBr pellet).

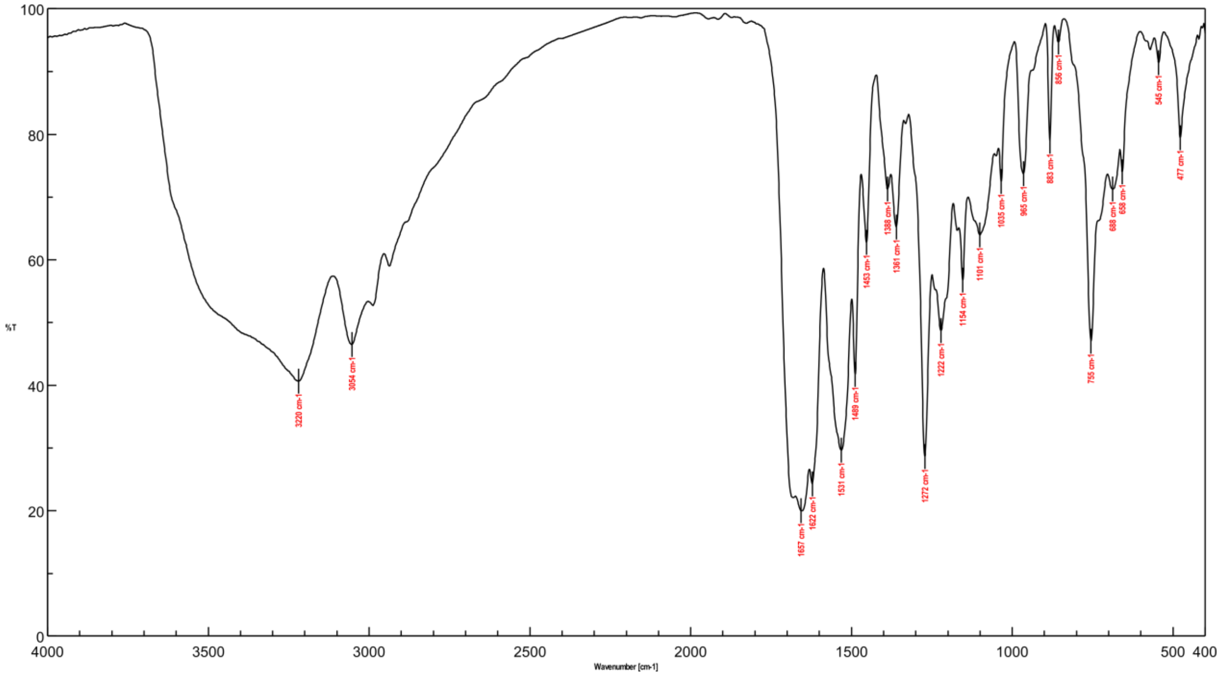

Figure S58. IR spectrum of **1b** (KBr pellet).

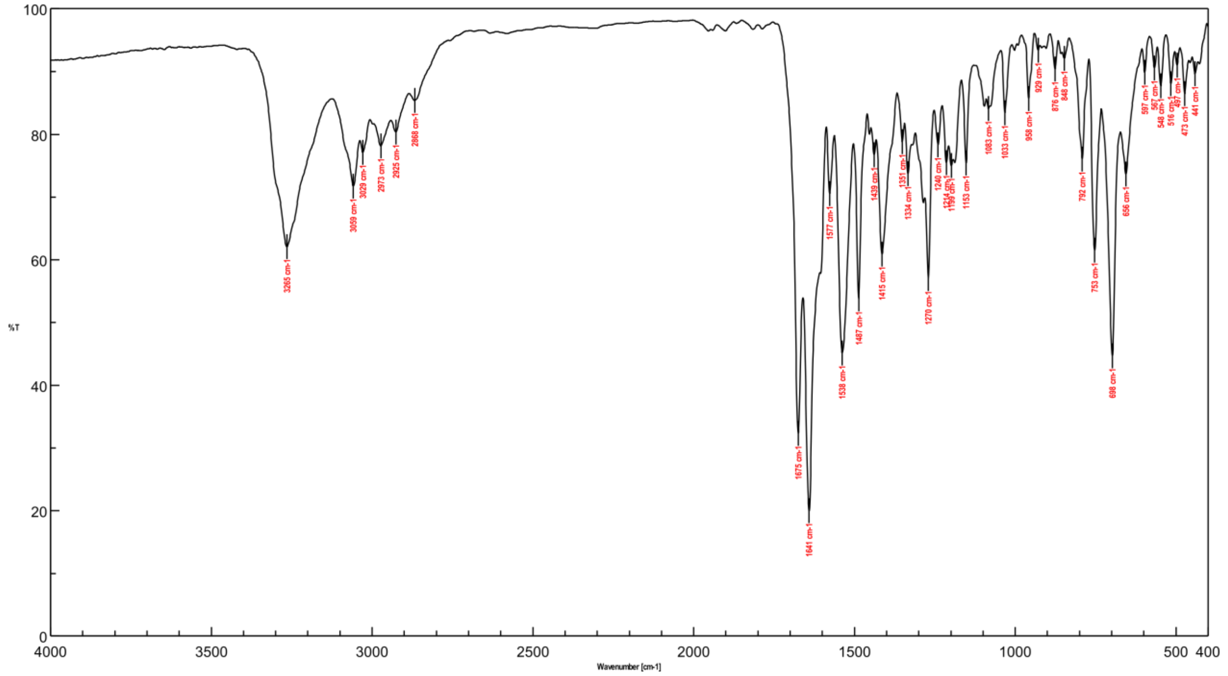

Figure S59. IR spectrum of **2a** (KBr pellet).

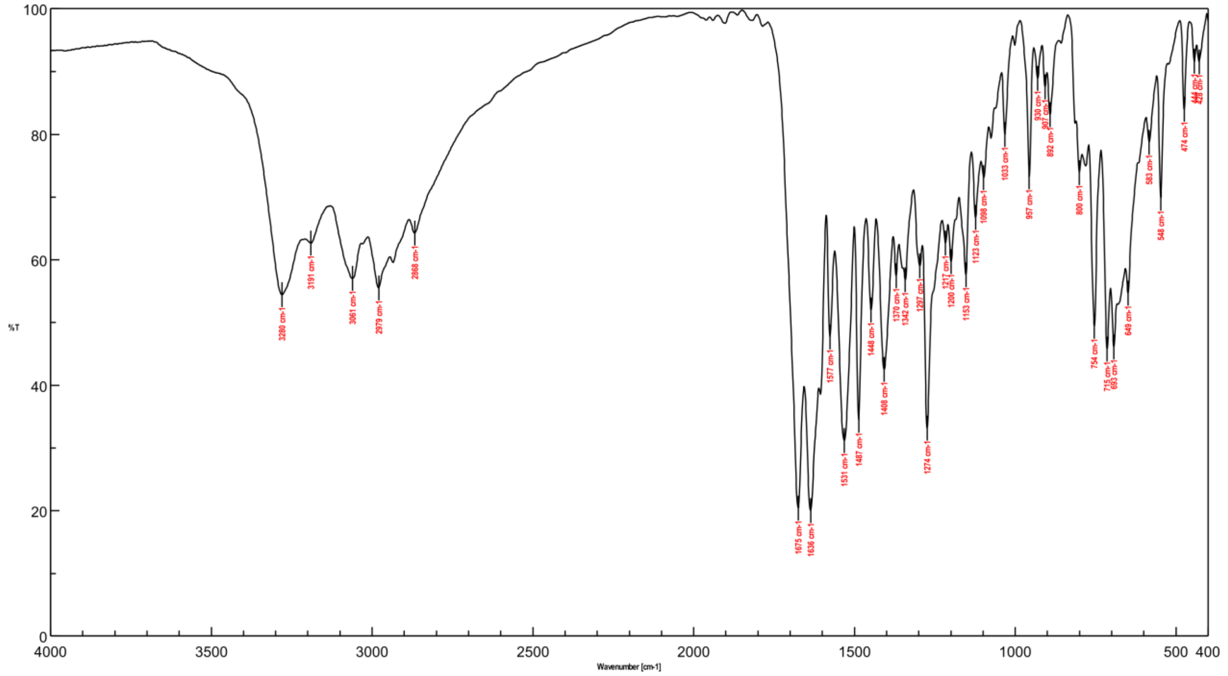

Figure S60. IR spectrum of **2b** (KBr pellet).

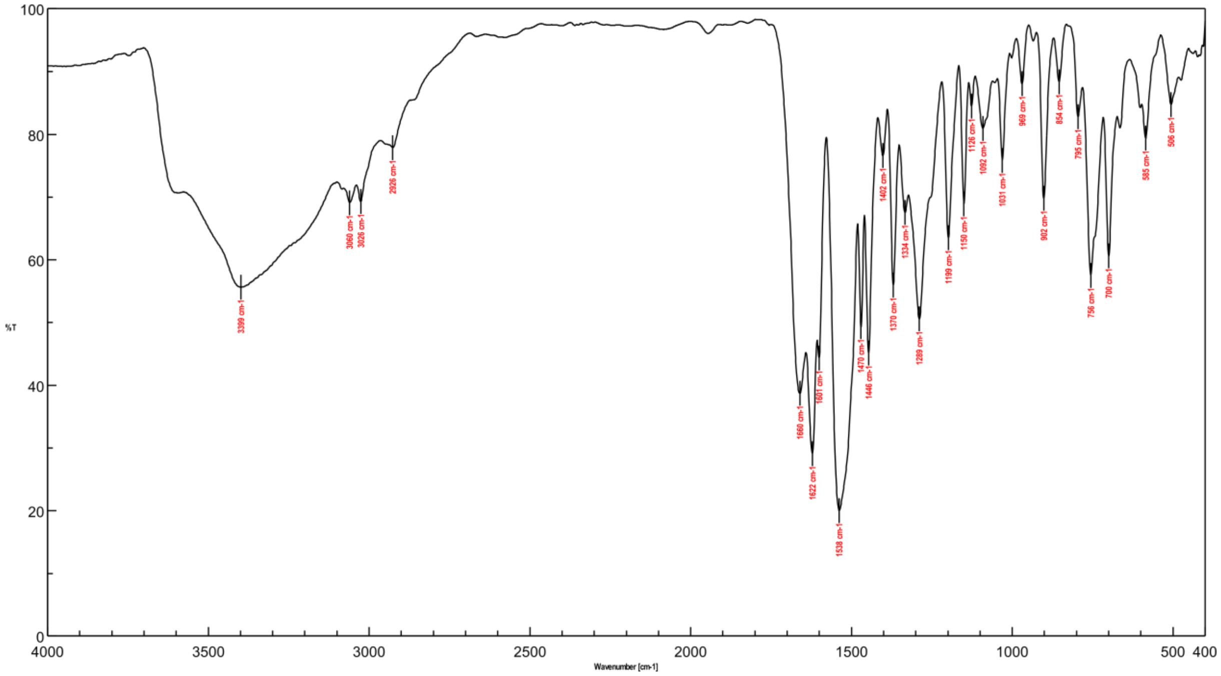

Figure S61. IR spectrum of **9a** (KBr pellet).

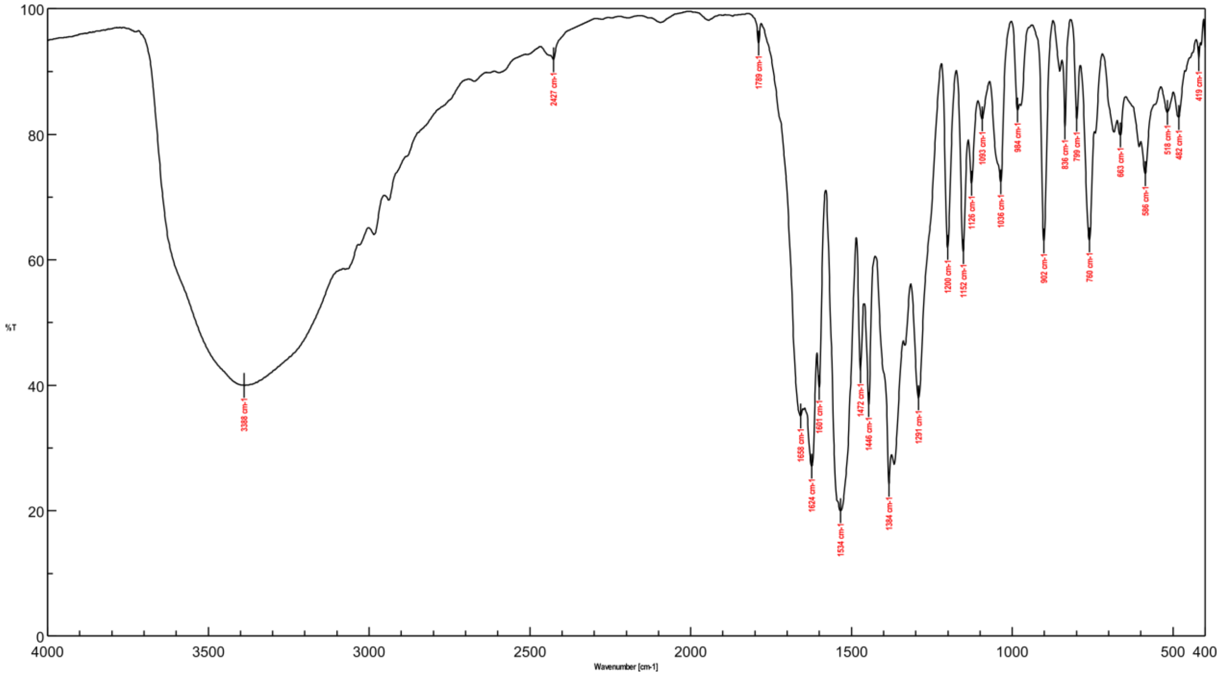

Figure S62. IR spectrum of **9b** (KBr pellet).

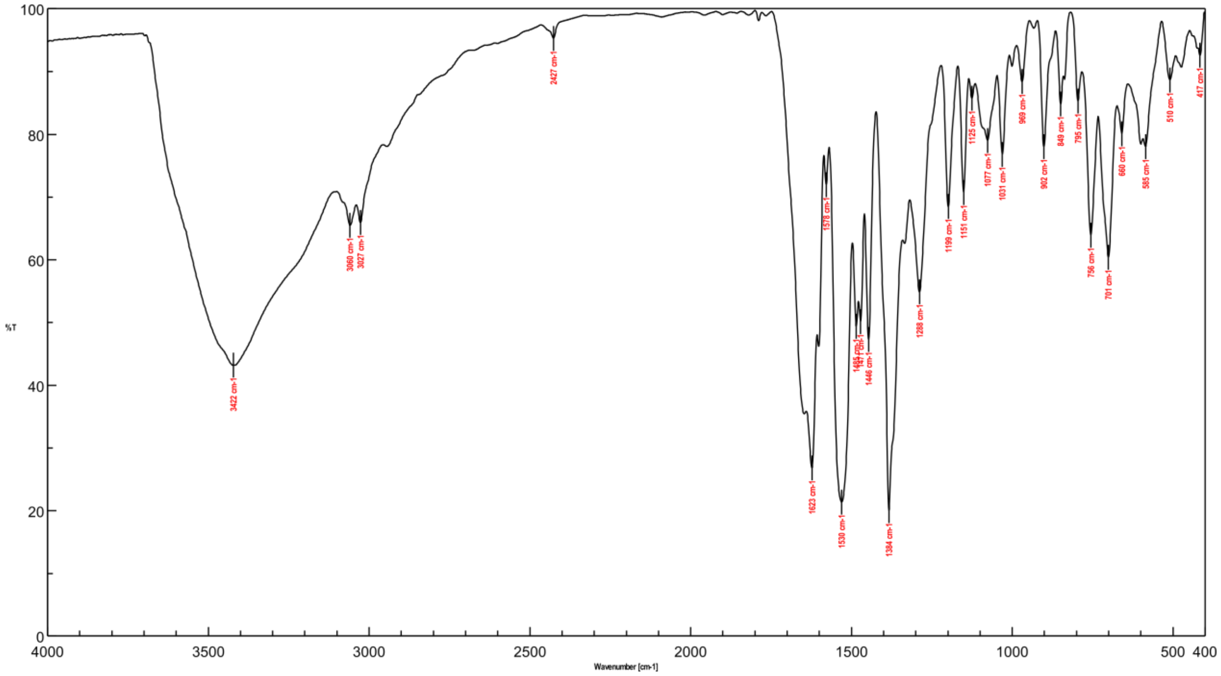

Figure S63. IR spectrum of **10a** (KBr pellet).

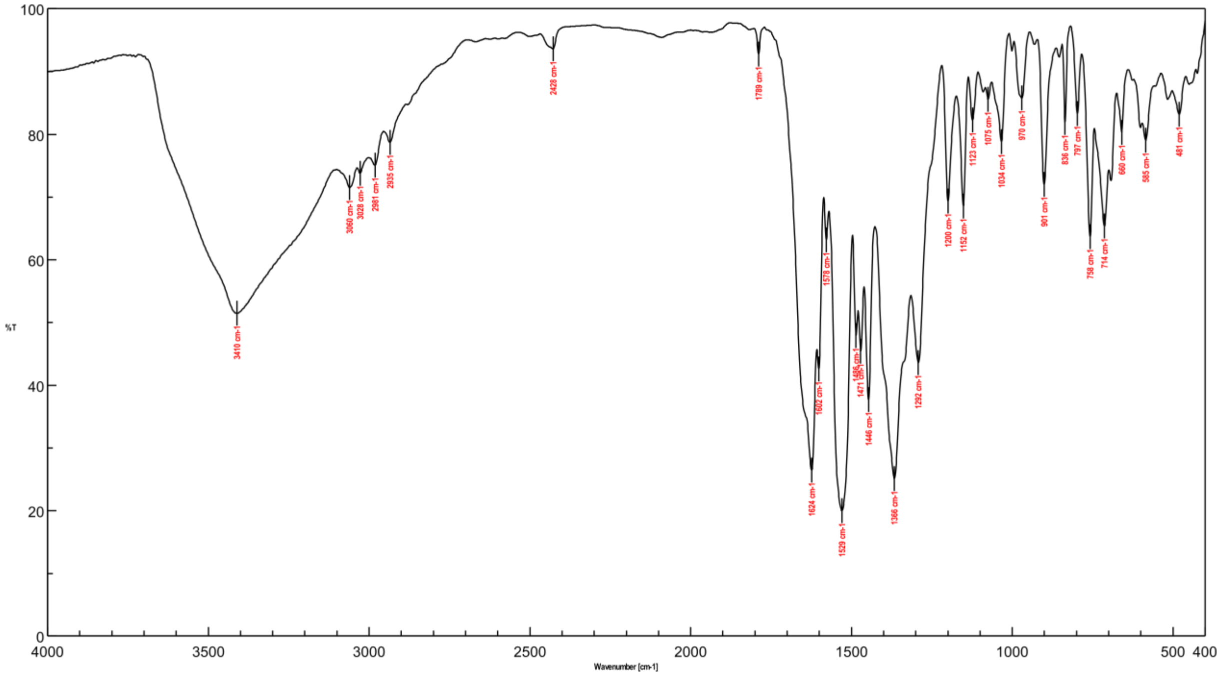

Figure S64. IR spectrum of **10b** (KBr pellet).

Crystal data

The crystals of *rac*-**9a** were grown by slow evaporation of methanol/water 3:1 mixture. X-ray measurements were carried out at the Agilent SuperNova diffractometer at λ=1.54184 Å. The structures were solved by using SHELXS.[2] The structures were refined by using SHELXL with anisotropic displacement parameters for all non-hydrogen atoms. All hydrogen atoms were included at geometrically predicted coordinates. Highly disordered solvent molecules were removed from the electron density by utilizing the solvent masking procedure in OLEX2.[3]

CCDC 2153029 contains the supplementary crystallographic data for this paper. These data are provided free of charge by The Cambridge Crystallographic Data Centre.

**Crystal data**	**9a**
Moiety formula	**9a**(CH3OH)(H_2O)$_8$
Empirical formula	$C_{114}H_{90}N_{18}O_{18}Ga_3Na_3$(CH3OH)($H_2O$)$_8$
Formula weight	2437.20
Temperature (K)	100
X-ray source	SuperNova (Cu)
Wavelength (Å)	1.54184
Crystal system	monoclinic
Space group	$P2_1/c$
Unit cell dimensions a/b/c (Å)	23.2825(4)<br>19.1083(7)<br>29.9978(6)
Unit cell angles α/β/γ (°)	90<br>103.599(2)<br>90
Unit cell volume (Å^3)	12971.6(6)
Z	4
Calculated density (g/cm^3)	1.248
Absorption coefficient (mm^{-1})	1.440
F(000)	5004.0
θ range for data collection (°)	2.3134 - 60.9496
Index ranges	-26 < h < 26<br>-21 < k < 21<br>-33 < l < 33
Reflections collected	19396
Independent reflections	9885 (R_{int} = 0.0757)
Completeness to θ_{max}	0.981
**Refinement statistics**	
Final R indices [>2σ(I)]	0.0776
R indices [all data]	0.1388
Goodness-of-fit	0.971
Extinction coefficient	-
Largest diff. peak and hole (e Å^{-3})	0.654/-0.462

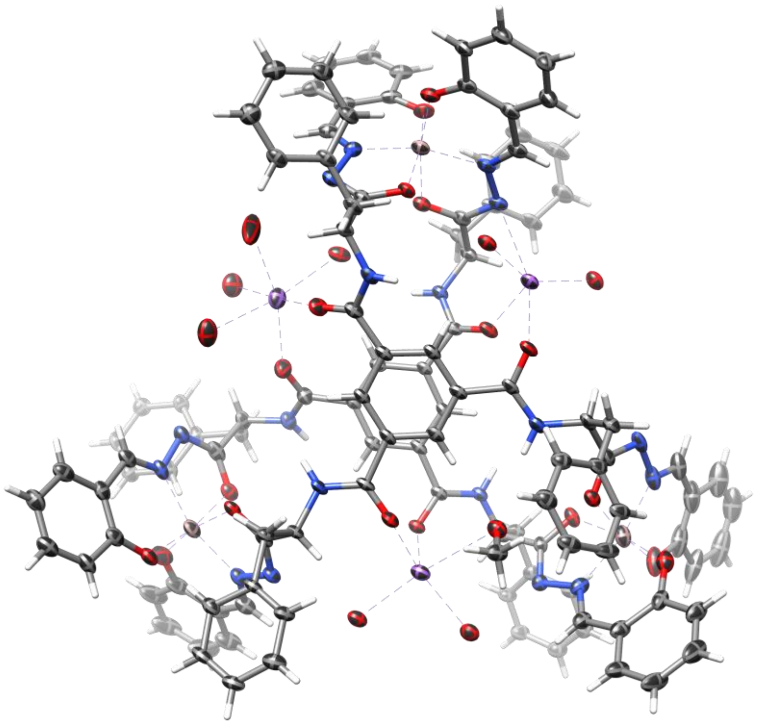

Figure S65. ORTEP representation of crystallographically independent part of **9a**. Thermal ellipsoids are depicted at 50% probability.

Explanation of level B alert for the crystal structure of **9a**

```
THETM01_ALERT_3_B The value of sine(theta_max)/wavelength is less than 0.575
                  Calculated sin(theta_max)/wavelength = 0.5665
```

The above alert comes from weak diffraction of the crystals.

Calculations

All calculations were performed within the density functional theory (DFT) approach using Gaussian 09 program suite.[4] Geometry was optimized with the B3LYP functional, employing the 6-31G basis set for H, C, N, O atoms and cc-PVDZ basis set for Ga, Na atoms. Solvent effects were considered within the SCRF theory using the polarized continuum model (PCM) approach to model the interaction with the solvent. Excited electronic states were determined at the wb97xd/6-31G/cc-PVDZ level by means of the time-dependent DFT (TD DFT) approach (100 excited states in each case). The ECD spectra were simulated by overlapping Gaussian functions for each transition where the width of the band at 1/e height is fixed at 0.2 eV and the resulting intensities of the combined spectra were scaled to the experimental values (using UV-VIS spectra as references).

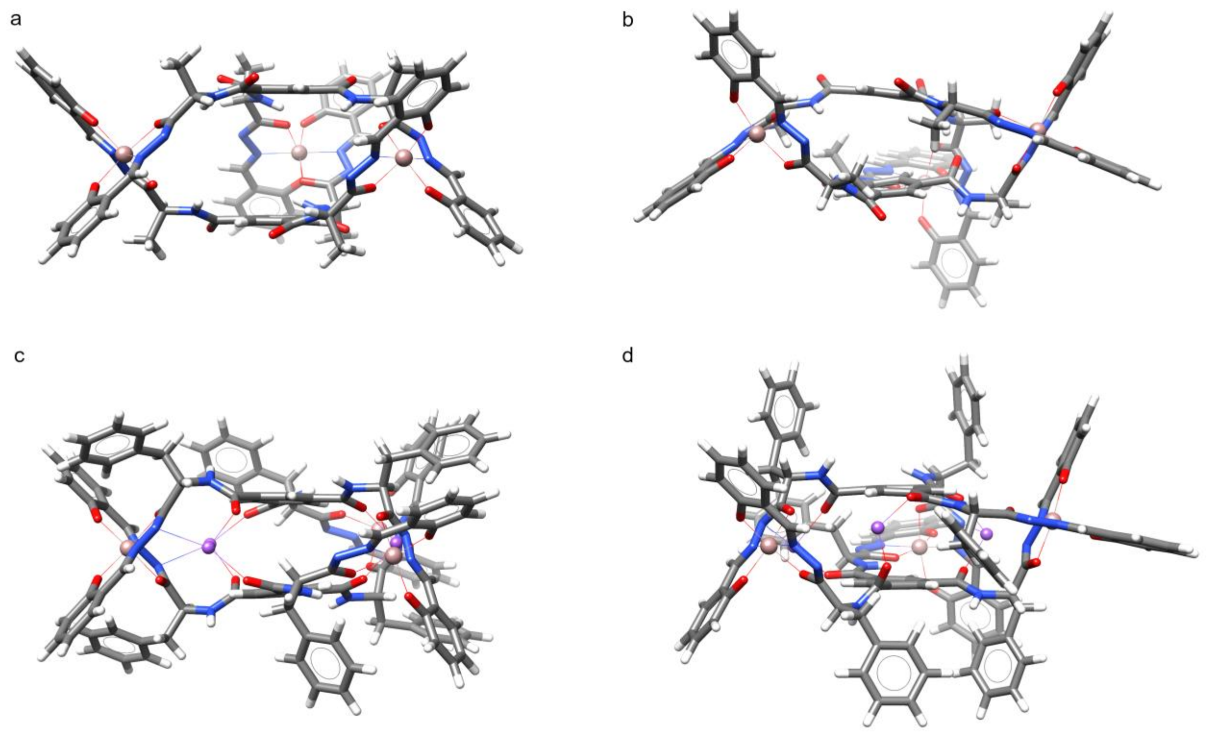

Figure S66. Optimized models of: (a) (Λ,*S*)-[**9b**-3Na]$^{3-}$, (b) (Δ, *S*)-[**9b**-3Na]$^{3-}$, (c) (Λ, *S*)-**9a**, (d) (Δ, *S*)-**9a**.

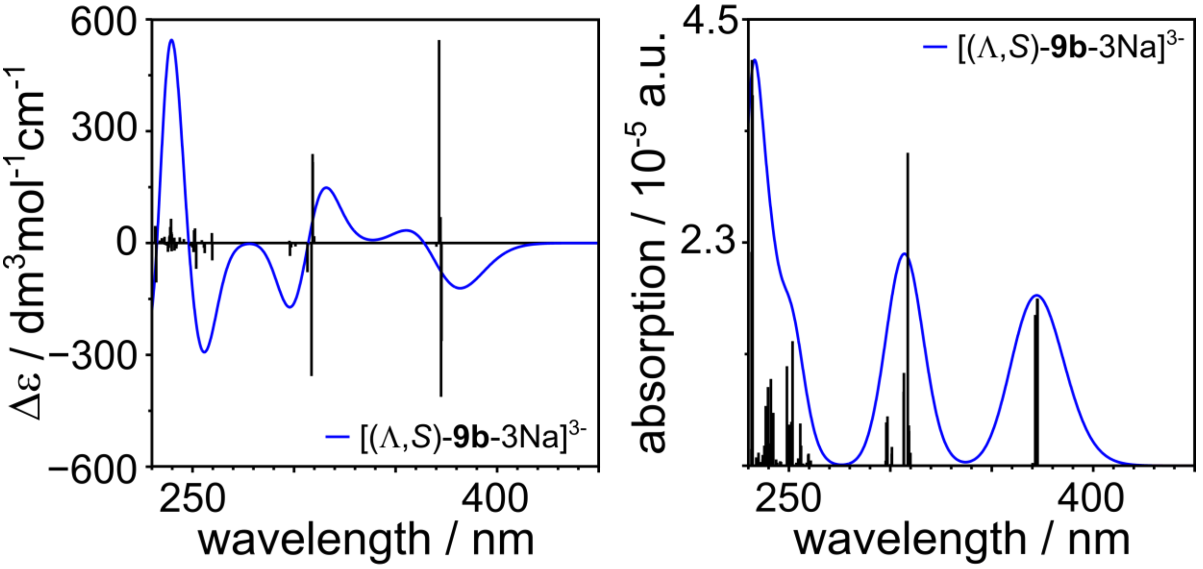

Figure S67. Calculated ECD and UV spectra of (Λ,*S*)-**9b** in methanol (TDDFT wb97xd/6-31G/cc-PVDZ, shift 35 nm).

```
 Excited State  15:      Singlet-A
4.5320 eV  273.58 nm  f=1.2957
<S**2>=0.000
     439 -> 456       -0.15488
     439 -> 458        0.10126
     440 -> 457        0.17640
     441 -> 456       -0.12473
     441 -> 458       -0.14079
     442 -> 452        0.13238
     442 -> 453        0.27365
     443 -> 451       -0.10494
     443 -> 452       -0.26889
     444 -> 451        0.27465

 Excited State  16:      Singlet-A
4.5636 eV  271.68 nm  f=0.3841
<S**2>=0.000
     439 -> 453       -0.12894
     440 -> 452        0.12991
     441 -> 451        0.22767
     441 -> 453        0.19568
     442 -> 458       -0.15840
     443 -> 457       -0.12353
     444 -> 456       -0.19446
     444 -> 458       -0.18975
     445 -> 451        0.16479
     445 -> 453        0.16578
     450 -> 456       -0.11198
     450 -> 458       -0.13979

 Excited State  17:      Singlet-A
4.5659 eV  271.55 nm  f=0.3064
<S**2>=0.000
     439 -> 451        0.12954
     439 -> 452        0.19271
     440 -> 451        0.12075
     440 -> 452       -0.16392
     440 -> 453       -0.15683
     442 -> 457       -0.19725
     443 -> 456        0.21428
     443 -> 458       -0.10582
     444 -> 457        0.10180
     446 -> 451       -0.11976
     446 -> 453        0.11330
     447 -> 452        0.16418
     448 -> 457       -0.11352
     449 -> 456       -0.11500

 Excited State  18:      Singlet-A
4.5768 eV  270.89 nm  f=0.0007
<S**2>=0.000
     439 -> 451        0.15208
     439 -> 453       -0.12260
     440 -> 452        0.19767
     441 -> 451       -0.11863
     441 -> 453       -0.15098
     442 -> 456        0.19563
     443 -> 457       -0.17235
     444 -> 458        0.17216
     445 -> 451       -0.13214
     445 -> 453       -0.11743
     446 -> 451       -0.10106
     447 -> 452       -0.13835
     448 -> 456        0.11081
     449 -> 456        0.10346
     449 -> 457        0.10996
     450 -> 458        0.12393

 Excited State  19:      Singlet-A
4.6642 eV  265.82 nm  f=0.0778
<S**2>=0.000
     440 -> 452       -0.11133
     446 -> 457       -0.10822
     447 -> 456       -0.23544
     447 -> 457       -0.22538
     448 -> 451       -0.10660
     448 -> 453        0.10271
     449 -> 452        0.32793
     449 -> 453        0.14751
     449 -> 456        0.15047
     449 -> 457        0.15413

 Excited State  20:      Singlet-A
4.6670 eV  265.66 nm  f=0.0500
<S**2>=0.000
     446 -> 455       -0.14495
     446 -> 456        0.24437
     446 -> 457       -0.22099
     446 -> 458       -0.13123
     447 -> 457        0.12028
     448 -> 451       -0.24274
     448 -> 452       -0.19210
     448 -> 453        0.21480
     448 -> 456       -0.13546
     448 -> 457        0.13584
     449 -> 452       -0.10517

 Excited State  21:      Singlet-A
4.6744 eV  265.24 nm  f=0.0075
<S**2>=0.000
     445 -> 454       -0.21087
     445 -> 456        0.25179
     445 -> 458        0.39185
     450 -> 451       -0.28546
     450 -> 453       -0.27714

 Excited State  22:      Singlet-A
4.7043 eV  263.56 nm  f=0.2046
<S**2>=0.000
     441 -> 451        0.16820
     441 -> 453        0.15941
     442 -> 458       -0.10991
     444 -> 456       -0.13889
     444 -> 458       -0.18955
     445 -> 451       -0.23139
     445 -> 453       -0.22869
     450 -> 454       -0.16188
     450 -> 456        0.20403
     450 -> 458        0.29403

 Excited State  23:      Singlet-A
4.7145 eV  262.98 nm  f=0.1794
<S**2>=0.000
     439 -> 452       -0.10530
     442 -> 457        0.13282
     443 -> 456       -0.15676
     446 -> 451       -0.16099
     446 -> 452       -0.10446
     446 -> 453        0.15611
     446 -> 455       -0.14341
     446 -> 456        0.14936
     446 -> 457       -0.16350
     447 -> 452        0.19351
     447 -> 456       -0.16747
     447 -> 457       -0.13198
     448 -> 455       -0.11455
     448 -> 456        0.14563
     448 -> 457       -0.15935
     449 -> 456       -0.13946
     449 -> 457       -0.10837
```

Figure S68. Excited states and transitions which contribute to the ECD band that is opposite to the experimental results.

Atomic coordinates for optimized models:

(Λ,*S*)-[**9b**-3Na]$^{3-}$ in vacuo
Total energy = -11125.382194 Hartrees
Number of negative eigenvalues: 0

No	Symbol	X	Y	Z
1	Ga	0.0000000	7.2449290	0.0000000
2	Ga	6.2742920	-3.6224640	0.0000000
3	Ga	-6.2742920	-3.6224640	0.0000000
4	C	0.5714750	1.2762130	3.0362130
5	C	-0.8209180	1.1417600	3.0303400
6	C	-1.3909710	-0.1431950	3.0362130
7	C	-0.5783340	-1.2818160	3.0303400
8	C	0.8194960	-1.1330180	3.0362130
9	C	1.3992520	0.1400560	3.0303400
10	H	1.0559640	2.2453370	3.0867740
11	H	-2.4725010	-0.2081770	3.0867740
12	H	1.4165370	-2.0371600	3.0867740
13	C	2.8806880	0.3747640	3.0816320
14	C	-1.1157890	-2.6821310	3.0816320
15	C	-1.7649000	2.3073670	3.0816320
16	N	3.6969100	-0.6168240	2.6302450
17	N	-2.3826400	-2.8932060	2.6302450
18	N	-1.3142700	3.5100300	2.6302450
19	O	3.3408420	1.4655490	3.5186570
20	O	-2.9396240	2.1604800	3.5186570
21	O	-0.4012180	-3.6260290	3.5186570
22	H	3.3431970	-1.4744380	2.2256020
23	H	-2.9484990	-2.1580750	2.2256020
24	H	-0.3946980	3.6325130	2.2256020
25	C	5.1573090	-0.4585730	2.6233990
26	H	5.3828380	0.5851340	2.3935140
27	C	-2.1815190	4.6956470	2.6233990
28	H	-3.1981610	4.3691080	2.3935140
29	C	-2.9757900	-4.2370740	2.6233990
30	H	-2.1846780	-4.9542420	2.3935140
31	C	5.7668350	-0.8167840	3.9973370
32	H	6.8518490	-0.6672760	3.9766770
33	H	5.3305030	-0.1709270	4.7653800
34	H	5.5599720	-1.8641640	4.2393370
35	C	-3.5907740	-4.5858340	3.9973370
36	H	-4.0038030	-5.6002380	3.9766770
37	H	-2.8132790	-4.5308880	4.7653800
38	H	-4.3944000	-3.8829950	4.2393370
39	C	-2.1760620	5.4026180	3.9973370
40	H	-2.8480470	6.2675140	3.9766770
41	H	-2.5172250	4.7018150	4.7653800
42	H	-1.1655730	5.7471590	4.2393370
43	C	5.7329260	-1.3341000	1.5320510
44	C	-4.0218270	-4.2978100	1.5320510
45	C	-1.7110990	5.6319100	1.5320510
46	O	-0.4107300	5.7807050	1.3678730
47	O	-4.8008730	-3.2460550	1.3678730
48	O	5.2116020	-2.5346500	1.3678730
49	N	-2.6488020	6.2692430	0.8447910
50	N	-4.1049230	-5.4285520	0.8447910
51	N	6.7537250	-0.8406910	0.8447910
52	N	-2.0512580	7.1573940	-0.0762970
53	N	7.2241140	-1.8022550	-0.0762970
54	N	-5.1728560	-5.3551390	-0.0762970
55	C	-5.3494620	-6.3821480	-0.8596190
56	H	-4.6478450	-7.2099110	-0.7445960
57	C	8.2018330	-1.4416950	-0.8596190
58	H	8.5678880	-0.4201960	-0.7445960
59	C	-2.8523720	7.8238440	-0.8596190
60	H	-3.9200430	7.6301070	-0.7445960
61	C	8.8311100	-2.2759520	-1.8504950
62	C	8.4429540	-3.6392120	-2.0901160
63	C	9.8832260	-1.7132230	-2.6127380
64	C	9.1504700	-4.3594540	-3.0926010
65	C	10.5574230	-2.4380860	-3.5865210
66	H	10.1573640	-0.6782680	-2.4182260
67	C	10.1777320	-3.7762860	-3.8201280
68	H	8.8462020	-5.3863550	-3.2636430
69	H	11.3585570	-1.9817250	-4.1599290
70	H	10.6933790	-4.3583430	-4.5809020
71	C	-6.3865880	-6.5099900	-1.8504950
72	C	-7.3731270	-5.4922070	-2.0901160
73	C	-6.4253080	-7.7025140	-2.6127380
74	C	-8.3506330	-5.7448130	-3.0926010
75	C	-7.3901560	-7.9239530	-3.5865210
76	H	-5.6660790	-8.4574010	-2.4182260
77	C	-8.3592260	-6.9260310	-3.8201280
78	H	-9.0878210	-4.9678580	-3.2636430
79	H	-7.3955030	-8.8459360	-4.1599290
80	H	-9.1211260	-7.0815660	-4.5809020
81	C	-2.4445230	8.7859420	-1.8504950
82	C	-1.0698270	9.1314180	-2.0901160
83	C	-3.4579190	9.4157360	-2.6127380
84	C	-0.7998380	10.1042670	-3.0926010
85	C	-3.1672670	10.3620390	-3.5865210
86	H	-4.4912840	9.1356690	-2.4182260
87	C	-1.8185060	10.7023180	-3.8201280
88	H	0.2416200	10.3542130	-3.2636430
89	H	-3.9630540	10.8276620	-4.1599290
90	H	-1.5722530	11.4399090	-4.5809020
91	O	7.4715340	-4.2596470	-1.4319470
92	O	-7.4247290	-4.3407150	-1.4319470
93	O	-0.0468050	8.6003610	-1.4319470
94	C	1.3909710	-0.1431950	-3.0362130
95	C	0.5783340	-1.2818160	-3.0303400
96	C	-0.8194960	-1.1330180	-3.0362130
97	C	-1.3992520	0.1400560	-3.0303400
98	C	-0.5714750	1.2762130	-3.0362130
99	C	0.8209180	1.1417600	-3.0303400
100	H	2.4725010	-0.2081770	-3.0867740
101	H	-1.4165370	-2.0371600	-3.0867740
102	H	-1.0559640	2.2453370	-3.0867740
103	C	1.7649000	2.3073670	-3.0816320
104	C	-2.8806880	0.3747640	-3.0816320
105	C	1.1157890	-2.6821310	-3.0816320
106	N	1.3142700	3.5100300	-2.6302450
107	N	-3.6969100	-0.6168240	-2.6302450
108	N	2.3826400	-2.8932060	-2.6302450
109	O	2.9396240	2.1604800	-3.5186570
110	O	0.4012180	-3.6260290	-3.5186570
111	O	-3.3408420	1.4655490	-3.5186570
112	H	0.3946980	3.6325130	-2.2256020
113	H	-3.3431970	-1.4744380	-2.2256020
114	H	2.9484990	-2.1580750	-2.2256020
115	C	2.1815190	4.6956470	-2.6233990
116	H	3.1981610	4.3691080	-2.3935140
117	C	2.9757900	-4.2370740	-2.6233990
118	H	2.1846780	-4.9542420	-2.3935140
119	C	-5.1573090	-0.4585730	-2.6233990
120	H	-5.3828380	0.5851340	-2.3935140
121	C	2.1760620	5.4026180	-3.9973370
122	H	2.8480470	6.2675140	-3.9766770
123	H	2.5172250	4.7018150	-4.7653800
124	H	1.1655730	5.7471590	-4.2393370
125	C	-5.7668350	-0.8167840	-3.9973370
126	H	-6.8518490	-0.6672760	-3.9766770
127	H	-5.3305030	-0.1709270	-4.7653800
128	H	-5.5599720	-1.8641640	-4.2393370
129	C	3.5907740	-4.5858340	-3.9973370
130	H	4.0038030	-5.6002380	-3.9766770
131	H	2.8132790	-4.5308880	-4.7653800
132	H	4.3944000	-3.8829950	-4.2393370
133	C	1.7110990	5.6319100	-1.5320510
134	C	-5.7329260	-1.3341000	-1.5320510
135	C	4.0218270	-4.2978100	-1.5320510
136	O	4.8008730	-3.2460550	-1.3678730
137	O	-5.2116020	-2.5346500	-1.3678730
138	O	0.4107300	5.7807050	-1.3678730
139	N	4.1049230	-5.4285520	-0.8447910
140	N	-6.7537250	-0.8406910	-0.8447910
141	N	2.6488020	6.2692430	-0.8447910
142	N	5.1728560	-5.3551390	0.0762970
143	N	2.0512580	7.1573940	0.0762970
144	N	-7.2241140	-1.8022550	0.0762970
145	C	-8.2018330	-1.4416950	0.8596190
146	H	-8.5678880	-0.4201960	0.7445960
147	C	2.8523720	7.8238440	0.8596190
148	H	3.9200430	7.6301070	0.7445960
149	C	5.3494620	-6.3821480	0.8596190

150	H	4.6478450	-7.2099110	0.7445960
151	C	2.4445230	8.7859420	1.8504950
152	C	1.0698270	9.1314180	2.0901160
153	C	3.4579190	9.4157360	2.6127380
154	C	0.7998380	10.1042670	3.0926010
155	C	3.1672670	10.3620390	3.5865210
156	H	4.4912840	9.1356690	2.4182260
157	C	1.8185060	10.7023180	3.8201280
158	H	-0.2416200	10.3542130	3.2636430
159	H	3.9630540	10.8276620	4.1599290
160	H	1.5722530	11.4399090	4.5809020
161	C	-8.8311100	-2.2759520	1.8504950
162	C	-8.4429540	-3.6392120	2.0901160
163	C	-9.8832260	-1.7132230	2.6127380
164	C	-9.1504700	-4.3594540	3.0926010
165	C	-10.5574230	-2.4380860	3.5865210
166	H	-10.1573640	-0.6782680	2.4182260
167	C	-10.1777320	-3.7762860	3.8201280
168	H	-8.8462020	-5.3863550	3.2636430
169	H	-11.3585570	-1.9817250	4.1599290
170	H	-10.6933790	-4.3583430	4.5809020
171	C	6.3865880	-6.5099900	1.8504950
172	C	7.3731270	-5.4922070	2.0901160
173	C	6.4253080	-7.7025140	2.6127380
174	C	8.3506330	-5.7448130	3.0926010
175	C	7.3901560	-7.9239530	3.5865210
176	H	5.6660790	-8.4574010	2.4182260
177	C	8.3592260	-6.9260310	3.8201280
178	H	9.0878210	-4.9678580	3.2636430
179	H	7.3955030	-8.8459360	4.1599290
180	H	9.1211260	-7.0815660	4.5809020
181	O	0.0468050	8.6003610	1.4319470
182	O	-7.4715340	-4.2596470	1.4319470
183	O	7.4247290	-4.3407150	1.4319470

(Δ, *S*)-[**9b**-3Na]$^{3-}$ in vacuo
Total energy = -11125.353873 Hartrees
Number of negative eigenvalues: 0

No	Symbol	X	Y	Z
1	Ga	-7.7825360	-0.2685630	0.1061950
2	Ga	3.7426290	5.5511890	0.0433310
3	C	-1.8086760	-1.0116630	2.0568770
4	C	-1.6274150	0.3135570	2.4741470
5	C	-0.3630480	0.9163530	2.3521850
6	C	0.6834260	0.2280880	1.7280120
7	C	0.4772070	-1.0830870	1.2748880
8	C	-0.7467320	-1.7294040	1.4831410
9	H	-2.7578770	-1.5248640	2.1759520
10	H	-0.2437870	1.9379170	2.6983790
11	H	1.2942810	-1.5655770	0.7519430
12	C	-0.9740100	-3.1741290	1.1157310
13	C	2.0382840	0.8200350	1.4744670
14	C	-2.7362010	1.1559410	3.0432120
15	N	0.1420440	-3.8683100	0.7897540
16	N	2.5864200	1.5550870	2.4794940
17	N	-3.9436600	1.0203520	2.4321060
18	O	-2.1308110	-3.6778210	1.1437480
19	O	-2.5087840	1.9729940	3.9764520
20	O	2.6534270	0.5827300	0.4002190
21	H	1.0673150	-3.4749340	0.9282160
22	H	2.0282730	1.7256600	3.3021460
23	H	-4.0742760	0.2985930	1.7346640
24	C	0.2038250	-5.2649170	0.3486520
25	H	-0.1824590	-5.9124910	1.1518140
26	C	-5.1399260	1.8492270	2.7043460
27	H	-5.5266950	1.5806290	3.7000840
28	C	3.9776390	2.0754390	2.4795930
29	H	4.0663820	2.5995490	3.4395920
30	C	-0.6118200	-5.5287550	-0.9278170
31	H	-1.6692830	-5.3353210	-0.7376740
32	H	-0.4680410	-6.5658350	-1.2411640
33	H	-0.2705640	-4.8751080	-1.7362080
34	C	5.0067120	0.9374560	2.4269890
35	H	4.8678950	0.2795210	3.2933640
36	H	6.0184090	1.3527280	2.4521470
37	H	4.9040050	0.3484620	1.5146970
38	C	-4.8374580	3.3532740	2.6673140
39	H	-4.1043030	3.6006660	3.4356390
40	H	-5.7615670	3.9143720	2.8300360
41	H	-4.4453470	3.6354100	1.6864700
42	C	1.6941130	-5.5612700	0.1569100
43	C	4.1632420	3.1548800	1.4223230
44	C	-6.1778810	1.4228640	1.6736450
45	O	-6.3799060	0.1260610	1.5325860
46	O	3.4392820	4.2447350	1.5679640
47	O	2.5494220	-4.6997020	0.6715630
48	N	-6.7731420	2.3658320	0.9598440
49	N	5.0505560	2.9505350	0.4588020
50	N	2.0358440	-6.6432980	-0.5252120
51	N	-7.6432940	1.7824680	0.0145730
52	N	3.4398970	-6.6994520	-0.6666310
53	N	5.0562580	4.0459630	-0.4281520
54	C	5.8682890	3.9855310	-1.4466220
55	H	6.4945730	3.0955080	-1.5230460
56	C	3.9279000	-7.7230210	-1.3088810
57	H	3.2112530	-8.4676240	-1.6600070
58	C	-8.2731270	2.5984180	-0.7841170
59	H	-8.0742840	3.6628960	-0.6500830
60	C	5.3222310	-7.9429450	-1.5984390
61	C	6.3562320	-7.0053090	-1.2512860
62	C	5.6723100	-9.1367230	-2.2739390
63	C	7.6931290	-7.3374330	-1.6089520
64	C	6.9860490	-9.4382750	-2.6088620
65	H	4.8734250	-9.8289940	-2.5332240
66	C	8.0001130	-8.5193480	-2.2671570
67	H	8.4618020	-6.6199960	-1.3430510
68	H	7.2267550	-10.361420	-3.1276970
69	H	9.0347100	-8.7369540	-2.5238810
70	C	5.9885730	4.9876750	-2.4745790
71	C	5.1686290	6.1676300	-2.5238330
72	C	6.9538630	4.7755890	-3.4886810
73	C	5.3768990	7.0711110	-3.6021140
74	C	7.1400020	5.6761890	-4.5290560
75	H	7.5570410	3.8715400	-3.4379150
76	C	6.3358970	6.8337560	-4.5762770
77	H	4.7484140	7.9546420	-3.6281240
78	H	7.8853180	5.4880920	-5.2957750
79	H	6.4648970	7.5476630	-5.3868920
80	C	-9.1944290	2.2090180	-1.8183340
81	C	-9.5216260	0.8376160	-2.0977670
82	C	-9.7935030	3.2341930	-2.5898710
83	C	-10.444126	0.5813510	-3.1504200
84	C	-10.692945	2.9571090	-3.6103350
85	H	-9.5265100	4.2649190	-2.3652600
86	C	-11.013480	1.6108060	-3.8851460
87	H	-10.678850	-0.4578800	-3.3531270
88	H	-11.136086	3.7609220	-4.1902880
89	H	-11.712933	1.3755500	-4.6843800
90	O	6.1364920	-5.8549130	-0.6284980
91	O	4.2291770	6.4584770	-1.6298850
92	O	-9.0158660	-0.1930960	-1.4331430
93	Ga	4.4860810	-5.1118110	0.1447180
94	C	-1.9083540	0.3854950	-2.4827030
95	C	-1.7370110	-0.9525080	-2.8578150
96	C	-0.4394490	-1.4476550	-3.0537610
97	C	0.6793370	-0.6529690	-2.7711100
98	C	0.4976850	0.6767710	-2.3549220
99	C	-0.7975640	1.2102920	-2.2435850
100	H	-2.8922780	0.8348830	-2.3956800
101	H	-0.3042870	-2.4617200	-3.4102160
102	H	1.3715220	1.2913690	-2.1648340
103	C	-1.0939430	2.6437520	-1.9076020
104	C	2.0410990	-1.2567460	-2.9893820
105	C	-2.8791840	-1.9116510	-3.0404600
106	N	-0.1348210	3.3687170	-1.2724060
107	N	2.9772970	-1.0024080	-2.0292850
108	N	-3.9595260	-1.7294920	-2.2306360
109	O	-2.2193670	3.1432450	-2.1914980
110	O	-2.7964860	-2.8758020	-3.8493450
111	O	2.2645040	-1.9635710	-4.0065900
112	H	0.7965050	3.0117180	-1.0973370
113	H	2.7457490	-0.4576870	-1.1968960
114	H	-4.0387540	-0.9182430	-1.6325120
115	C	-0.3558440	4.7701060	-0.8945020
116	H	-0.7148850	5.3073400	-1.7848810
117	C	-5.0570940	-2.7038500	-2.1382710

118	H	-5.4918020	-2.8236650	-3.1428940
119	C	4.3583400	-1.5118640	-2.1346190
120	H	4.4097290	-2.0103730	-3.1062970
121	C	-1.4205840	4.9125090	0.2107240
122	H	-2.3688210	4.5064230	-0.1475140
123	H	-1.5369950	5.9666670	0.4730870
124	H	-1.1091300	4.3684470	1.1084690
125	C	5.3579080	-0.3483100	-2.0579360
126	H	5.1946610	0.3269320	-2.9054410
127	H	6.3869340	-0.7225410	-2.0944480
128	H	5.2335950	0.2187030	-1.1314900
129	C	-4.5599680	-4.0773720	-1.6474830
130	H	-3.8528660	-4.4836400	-2.3732910
131	H	-5.4101310	-4.7530970	-1.5303610
132	H	-4.0605540	-3.9712300	-0.6791640
133	C	0.9841890	5.3598540	-0.4755340
134	C	4.6025990	-2.5963400	-1.0894000
135	C	-6.1333270	-2.1248860	-1.2267990
136	O	-6.2116830	-0.8153730	-1.0936630
137	O	4.2326360	-3.8229560	-1.3907270
138	O	2.0775810	4.7036660	-0.7960380
139	N	-6.9358150	-2.9925790	-0.6208360
140	N	5.1603150	-2.2444110	0.0685150
141	N	0.9572100	6.5011600	0.2047780
142	N	-7.8692780	-2.3191020	0.1871940
143	N	2.2606270	6.8739880	0.5831160
144	N	5.2591650	-3.3651130	0.9183650
145	C	5.8094960	-3.1843570	2.0864490
146	H	6.1712540	-2.1791820	2.3091220
147	C	2.3928690	7.9940320	1.2371780
148	H	1.4820480	8.5705900	1.4083870
149	C	-8.7008660	-3.0589390	0.8668520
150	H	-8.6007510	-4.1388540	0.7460790
151	C	3.6293820	8.5160320	1.7622620
152	C	4.8794550	7.8102670	1.6746190
153	C	3.5905050	9.7714020	2.4148320
154	C	6.0240020	8.4232860	2.2567100
155	C	4.7241550	10.3499450	2.9719470
156	H	2.6332870	10.2861180	2.4742070
157	C	5.9498610	9.6579220	2.8859080
158	H	6.9594690	7.8791010	2.1842300
159	H	4.6663050	11.3149970	3.4666000
160	H	6.8479620	10.0945220	3.3177310
161	C	5.9635640	-4.1966170	3.1011970
162	C	5.4663560	-5.5379600	2.9565200
163	C	6.6206980	-3.8307700	4.3003250
164	C	5.6654750	-6.4353370	4.0419520
165	C	6.8063570	-4.7282930	5.3443420
166	H	6.9839860	-2.8090710	4.3916430
167	C	6.3179340	-6.0432570	5.2024760
168	H	5.2820940	-7.4426290	3.9197780
169	H	7.3132290	-4.4205730	6.2540060
170	H	6.4507050	-6.7589440	6.0110680
171	C	-9.7242840	-2.5719380	1.7554400
172	C	-9.9441090	-1.1737020	2.0049310
173	C	-10.539473	-3.5263210	2.4097300
174	C	-10.983314	-0.8205850	2.9100350
175	C	-11.549604	-3.1552520	3.2881740
176	H	-10.354201	-4.5797300	2.2085350
177	C	-11.764227	-1.7832130	3.5339400
178	H	-11.136268	0.2379830	3.0903160
179	H	-12.159936	-3.9077610	3.7785380
180	H	-12.549433	-1.4742680	4.2206730
181	O	5.0211770	6.6246530	1.0954820
182	O	4.8299540	-5.9819170	1.8780250
183	O	-9.2359010	-0.2029630	1.4414190

(Λ, *S*)-[**9b**-3Na]$^{3-}$ in methanol
Total energy = -11125.742158 Hartrees
Number of negative eigenvalues: 0

No	Symbol	X	Y	Z
1	Ga	4.6230420	-5.6254900	-0.0155950
2	Ga	2.6780000	6.7378460	0.0275250
3	Ga	-7.2674500	-1.1127830	0.0062480
4	C	1.1271430	-0.6267970	-2.3027230
5	C	-0.1086060	-1.2540360	-2.1054040
6	C	-1.2646050	-0.4709920	-1.9569680
7	C	-1.1878320	0.9262780	-1.9880050
8	C	0.0601430	1.5392850	-2.1746350
9	C	1.2210430	0.7731960	-2.3277600
10	H	2.0450120	-1.1852660	-2.4443810
11	H	-2.1967310	-1.0010410	-1.8021650
12	H	0.0676480	2.6223360	-2.1874750
13	C	2.5939470	1.3649260	-2.4777960
14	C	-2.3603800	1.8432480	-1.7807440
15	C	-0.2843020	-2.7430430	-2.0296660
16	N	2.7177860	2.7026970	-2.2996600
17	N	-3.5883870	1.2922760	-1.6311200
18	N	0.8219110	-3.5046020	-1.8462110
19	O	3.5923200	0.6269010	-2.7213420
20	O	-1.4391360	-3.2557670	-2.1121990
21	O	-2.1871910	3.0972320	-1.7291200
22	H	1.9205240	3.2911490	-2.0974800
23	H	-3.7544310	0.2974220	-1.7114750
24	H	1.7443930	-3.1041070	-1.7332190
25	C	4.0143230	3.3991730	-2.2713830
26	H	4.7646080	2.7025900	-1.8919900
27	C	0.7559160	-4.9683650	-1.7401700
28	H	-0.1470950	-5.2298950	-1.1830480
29	C	-4.7790480	2.1004430	-1.3297510
30	H	-4.4836160	2.8952060	-0.6406910
31	C	4.4280730	3.8974230	-3.6706190
32	H	5.4028120	4.3946650	-3.6244230
33	H	4.5002900	3.0489610	-4.3573700
34	H	3.6894080	4.6065050	-4.0568550
35	C	-5.3838990	2.7259500	-2.6056890
36	H	-6.2525960	3.3410600	-2.3494150
37	H	-4.6365330	3.3575580	-3.0935740
38	H	-5.6994670	1.9421130	-3.3017070
39	C	0.7037570	-5.6347580	-3.1333410
40	H	0.6211380	-6.7213190	-3.0297140
41	H	-0.1668550	-5.2661520	-3.6829060
42	H	1.6097170	-5.4014780	-3.7019990
43	C	3.8558190	4.5395320	-1.2866100
44	C	-5.7746540	1.1953330	-0.6339740
45	C	1.9648150	-5.4349780	-0.9542150
46	O	3.1026540	-4.7837580	-1.1267500
47	O	-5.8722740	-0.0497290	-1.0682410
48	O	2.7674910	5.2830120	-1.4099480
49	N	1.8048820	-6.4911790	-0.1735270
50	N	-6.4965730	1.7172310	0.3441960
51	N	4.7881770	4.7067010	-0.3641480
52	N	3.0343280	-6.8338670	0.4456420
53	N	4.4448750	5.7983690	0.4785850
54	N	-7.3917810	0.7447500	0.8610380
55	C	-8.1821910	1.1242020	1.8299560
56	H	-8.1078740	2.1643490	2.1483980
57	C	5.2737480	6.0795090	1.4489630
58	H	6.1741970	5.4691120	1.5228300
59	C	3.0209650	-7.8623370	1.2520630
60	H	2.0702040	-8.3827090	1.3706960
61	C	5.0896400	7.1180710	2.4263130
62	C	3.9333190	7.9672270	2.4698030
63	C	6.1098000	7.2875610	3.3991090
64	C	3.8655880	8.9464930	3.4938570
65	C	6.0204070	8.2543120	4.3896380
66	H	6.9773480	6.6344430	3.3530950
67	C	4.8825190	9.0877280	4.4295150
68	H	2.9880170	9.5834660	3.5198610
69	H	6.8111930	8.3674970	5.1227350
70	H	4.7984860	9.8483320	5.2001290
71	C	-9.1297110	0.2832930	2.5106440
72	C	-9.2897120	-1.1154120	2.2312780
73	C	-9.9273560	0.8827610	3.5203410
74	C	-10.248513	-1.8389130	2.9857680
75	C	-10.860798	0.1536970	4.2423310
76	H	-9.7915030	1.9422180	3.7214590
77	C	-11.015593	-1.2210800	3.9654640
78	H	-10.361941	-2.8957390	2.7692640
79	H	-11.460834	0.6316510	5.0085730
80	H	-11.741440	-1.8048060	4.5238890
81	C	4.1491970	-8.3567820	1.9936790
82	C	5.4460070	-7.7430300	1.9618590
83	C	3.9467870	-9.5057070	2.8025820
84	C	6.4750450	-8.3220950	2.7480580
85	C	4.9704640	-10.054028	3.5612050
86	H	2.9579230	-9.9566220	2.8171630
87	C	6.2445470	-9.4489380	3.5271630

88	H	7.4522470	-7.8521910	2.7194090
89	H	4.7946960	-10.932763	4.1715780
90	H	7.0554260	-9.8667490	4.1165490
91	O	2.9165420	7.8840020	1.6013690
92	O	-8.5809140	-1.7736370	1.3039850
93	O	5.7342590	-6.6544510	1.2360440
94	C	0.8194490	0.9095140	2.2463080
95	C	-0.5473420	1.1307200	2.0402780
96	C	-1.4111630	0.0316120	1.9096760
97	C	-0.9160580	-1.2763720	1.9697910
98	C	0.4585630	-1.4806870	2.1618170
99	C	1.3324530	-0.3958820	2.2969960
100	H	1.5265860	1.7204410	2.3751030
101	H	-2.4585390	0.2513740	1.7408570
102	H	0.7953310	-2.5097890	2.1969200
103	C	2.8187340	-0.5402460	2.4642810
104	C	-1.7614540	-2.5056810	1.7900710
105	C	-1.1669020	2.4949730	1.9380300
106	N	3.3490800	-1.7738400	2.2815140
107	N	-3.1060460	-2.3517150	1.7323480
108	N	-0.3423960	3.5592540	1.7830500
109	O	3.5388870	0.4658070	2.7291640
110	O	-2.4257920	2.6293670	1.9768360
111	O	-1.2184640	-3.6451360	1.6865410
112	H	2.7736610	-2.5739460	2.0547400
113	H	-3.5544410	-1.4531600	1.8537100
114	H	0.6624110	3.4635510	1.7105070
115	C	4.7966070	-2.0419880	2.2967470
116	H	5.3106840	-1.1488770	1.9358330
117	C	-0.8513950	4.9307520	1.6535150
118	H	-1.7723420	4.8980580	1.0667070
119	C	-4.0209620	-3.4732300	1.4703680
120	H	-3.5071880	-4.1810790	0.8171290
121	C	5.2968020	-2.3940750	3.7123150
122	H	6.3767530	-2.5758880	3.7001330
123	H	5.0895210	-1.5642730	4.3943830
124	H	4.7924110	-3.2924020	4.0811530
125	C	-4.4362580	-4.1849030	2.7753540
126	H	-5.0996140	-5.0273780	2.5533690
127	H	-3.5466760	-4.5642130	3.2864200
128	H	-4.9597680	-3.4895650	3.4391440
129	C	-1.1521100	5.5581630	3.0332770
130	H	-1.5592680	6.5664240	2.9075210
131	H	-1.8865700	4.9450680	3.5631390
132	H	-0.2381780	5.6181090	3.6331080
133	C	5.0266230	-3.1741830	1.3171290
134	C	-5.2187890	-2.9070710	0.7369440
135	C	0.1800720	5.7432430	0.8962030
136	O	1.4568790	5.4654990	1.0996450
137	O	-5.7038180	-1.7561070	1.1719580
138	O	4.2197180	-4.2187500	1.4166240
139	N	-0.2725490	6.7053830	0.1087790
140	N	-5.7045730	-3.6004910	-0.2795650
141	N	5.9925690	-3.0439860	0.4235320
142	N	0.8101210	7.4078180	-0.4798720
143	N	6.0301960	-4.1878920	-0.4186500
144	N	-6.8155870	-2.9209550	-0.8446380
145	C	-7.4108310	-3.4975440	-1.8549650
146	H	-7.0272400	-4.4701650	-2.1645480
147	C	6.9382000	-4.2005960	-1.3584540
148	H	7.6111140	-3.3440430	-1.4080540
149	C	0.5062000	8.3922820	-1.2841300
150	H	-0.5541490	8.6031330	-1.4255190
151	C	7.1125060	-5.2435390	-2.3330030
152	C	6.2693730	-6.4024900	-2.4104320
153	C	8.1693360	-5.0931370	-3.2691010
154	C	6.5356670	-7.3519060	-3.4301580
155	C	8.4108980	-6.0378760	-4.2554950
156	H	8.7963010	-4.2082110	-3.1981670
157	C	7.5796360	-7.1756310	-4.3293210
158	H	5.8926370	-8.2239230	-3.4824730
159	H	9.2239830	-5.9038910	-4.9601180
160	H	7.7559830	-7.9228460	-5.0974210
161	C	-8.5168520	-2.9497040	-2.5929560
162	C	-9.0730920	-1.6513830	-2.3372690
163	C	-9.0595920	-3.7408810	-3.6392560
164	C	-10.151077	-1.2179590	-3.1511330
165	C	-10.116668	-3.2953120	-4.4190100
166	H	-8.6243450	-4.7198330	-3.8220810
167	C	-10.661353	-2.0188330	-4.1650640
168	H	-10.564502	-0.2349800	-2.9526910
169	H	-10.516939	-3.9166940	-5.2122670
170	H	-11.488217	-1.6560320	-4.7684500
171	C	1.4498930	9.2101120	-1.9969220
172	C	2.8704280	9.0157970	-1.9334110
173	C	0.9293400	10.2518860	-2.8088010
174	C	3.6947440	9.8837510	-2.6947000
175	C	1.7573310	11.0894940	-3.5415100
176	H	-0.1487540	10.3846290	-2.8464000
177	C	3.1533640	10.8953590	-3.4779240
178	H	4.7671750	9.7297210	-2.6424920
179	H	1.3390000	11.8799420	-4.1545470
180	H	3.8138940	11.5427210	-4.0472070
181	O	5.2459600	-6.6345630	-1.5775520
182	O	-8.6263740	-0.8265760	-1.3802290
183	O	3.4568210	8.0617050	-1.1984210

(Δ, *S*)-[**9b**-3Na]$^{3-}$ in methanol

Total energy = -11125.722477 Hartrees

Number of negative eigenvalues: 0

No	Symbol	X	Y	Z
1	Ga	-7.7869130	-0.2516340	0.0697870
2	Ga	3.7171480	5.5581480	0.0221420
3	C	-1.7832630	-0.8288120	2.0249690
4	C	-1.5880720	0.4992070	2.4259830
5	C	-0.3008220	1.0604210	2.3805270
6	C	0.7632990	0.3225960	1.8494000
7	C	0.5470660	-0.9916850	1.4123450
8	C	-0.7105780	-1.5930780	1.5388960
9	H	-2.7554790	-1.3039420	2.0960540
10	H	-0.1653780	2.0838490	2.7126010
11	H	1.3896050	-1.5094810	0.9713710
12	C	-0.9704810	-3.0311760	1.1716260
13	C	2.1417640	0.8793730	1.6557950
14	C	-2.7136820	1.3755090	2.8970640
15	N	0.1145810	-3.7509830	0.8089910
16	N	2.6366850	1.6650500	2.6427860
17	N	-3.8754570	1.2562660	2.2051410
18	O	-2.1411170	-3.5093850	1.2210710
19	O	-2.5441360	2.2018540	3.8372550
20	O	2.8044680	0.5798890	0.6195220
21	H	1.0496660	-3.3756780	0.9234550
22	H	2.0674960	1.8155920	3.4636590
23	H	-3.9436860	0.5578120	1.4773200
24	C	0.1372930	-5.1627100	0.4029050
25	H	-0.2156010	-5.7806190	1.2436130
26	C	-5.0868260	2.0812030	2.4302830
27	H	-5.4514490	1.8746060	3.4472460
28	C	4.0104780	2.2346590	2.6754330
29	H	4.0341670	2.8186460	3.6021060
30	C	-0.7409330	-5.4550560	-0.8230580
31	H	-1.7854260	-5.2425710	-0.5915310
32	H	-0.6347820	-6.5046280	-1.1062290
33	H	-0.4312920	-4.8351320	-1.6700870
34	C	5.0852190	1.1430170	2.7534700
35	H	4.9248580	0.5397480	3.6530020
36	H	6.0765220	1.6009570	2.8140190
37	H	5.0578180	0.4913530	1.8800100
38	C	-4.8054100	3.5830660	2.2928250
39	H	-4.0541750	3.8838410	3.0239930
40	H	-5.7240400	4.1496970	2.4652770
41	H	-4.4457310	3.8156300	1.2872130
42	C	1.6138720	-5.4829540	0.1547450
43	C	4.1921530	3.2512610	1.5557390
44	C	-6.1314770	1.5652910	1.4505570
45	O	-6.3817860	0.2658690	1.4690660
46	O	3.4405050	4.3362560	1.6257450
47	O	2.5069590	-4.6682270	0.6950200
48	N	-6.6922120	2.4271460	0.6187550
49	N	5.0808460	3.0147330	0.6032530
50	N	1.9149130	-6.5315070	-0.5898260
51	N	-7.5879300	1.7594470	-0.2524430
52	N	3.3213870	-6.6304980	-0.7508600
53	N	5.0525620	4.0528740	-0.3592090
54	C	5.8842420	3.9591410	-1.3630300
55	H	6.5567890	3.1008740	-1.3716270
56	C	3.7631790	-7.6418500	-1.4509550
57	H	3.0192430	-8.3444470	-1.8275940
58	C	-8.1921470	2.4946490	-1.1491660
59	H	-7.9676120	3.5615590	-1.1440730

60	C	5.1422850	-7.8954410	-1.7706860
61	C	6.2151850	-7.0181530	-1.3973200
62	C	5.4352780	-9.0698800	-2.5123540
63	C	7.5312060	-7.3722800	-1.7910860
64	C	6.7318570	-9.3956600	-2.8823300
65	H	4.6108780	-9.7213190	-2.7903680
66	C	7.7843300	-8.5313860	-2.5137610
67	H	8.3363860	-6.7033890	-1.5066410
68	H	6.9335950	-10.298666	-3.4475810
69	H	8.8043750	-8.7726480	-2.7979300
70	C	5.9727070	4.8887290	-2.4572340
71	C	5.1071410	6.0245230	-2.6012940
72	C	6.9598050	4.6416840	-3.4472190
73	C	5.2808190	6.8530600	-3.7393040
74	C	7.1124110	5.4703290	-4.5492570
75	H	7.6052670	3.7758470	-3.3244170
76	C	6.2586700	6.5847510	-4.6889050
77	H	4.6208340	7.7078660	-3.8412430
78	H	7.8737770	5.2638700	-5.2931310
79	H	6.3651290	7.2411060	-5.5475540
80	C	-9.1148690	2.0106180	-2.1382950
81	C	-9.4827770	0.6290250	-2.2636350
82	C	-9.6724150	2.9603910	-3.0345830
83	C	-10.394523	0.2746900	-3.2913560
84	C	-10.564189	2.5883580	-4.0293120
85	H	-9.3817600	4.0020710	-2.9264160
86	C	-10.922337	1.2288200	-4.1517290
87	H	-10.665988	-0.7715880	-3.3815340
88	H	-10.978527	3.3292150	-4.7038570
89	H	-11.618134	0.9229750	-4.9274600
90	O	6.0403270	-5.8833720	-0.7068990
91	O	4.1415400	6.3394200	-1.7268380
92	O	-9.0138260	-0.3384900	-1.4656350
93	Ga	4.4285270	-5.1406240	0.1309660
94	C	-1.8801320	0.2413050	-2.2423390
95	C	-1.6660640	-1.0991660	-2.5849870
96	C	-0.3557920	-1.5517330	-2.7863560
97	C	0.7368790	-0.7066330	-2.5548680
98	C	0.5149840	0.6275900	-2.1774780
99	C	-0.7975080	1.1131030	-2.0500460
100	H	-2.8794960	0.6516910	-2.1504410
101	H	-0.1880510	-2.5716920	-3.1089460
102	H	1.3671200	1.2842150	-2.0403670
103	C	-1.1354400	2.5470050	-1.7587610
104	C	2.1087780	-1.2705870	-2.7906550
105	C	-2.7793610	-2.0920480	-2.7518510
106	N	-0.1921760	3.3157020	-1.1651010
107	N	3.0757640	-0.9345070	-1.8963270
108	N	-3.8720290	-1.9138190	-1.9679500
109	O	-2.2811520	3.0001880	-2.0527800
110	O	-2.6608780	-3.0686190	-3.5470420
111	O	2.3116640	-2.0359210	-3.7764790
112	H	0.7387830	2.9702420	-0.9671920
113	H	2.8534180	-0.3899280	-1.0589620
114	H	-3.9462740	-1.1037160	-1.3676220
115	C	-0.4086620	4.7355170	-0.8530520
116	H	-0.7566810	5.2342460	-1.7694550
117	C	-5.0129630	-2.8480790	-1.9302340
118	H	-5.4107600	-2.9340200	-2.9532430
119	C	4.4588970	-1.4447390	-2.0210470
120	H	4.5131190	-1.8992490	-3.0132930
121	C	-1.4729940	4.9375010	0.2415570
122	H	-2.4240640	4.5201510	-0.0937500
123	H	-1.5935070	6.0033300	0.4465750
124	H	-1.1670410	4.4387590	1.1669530
125	C	5.4729620	-0.3014710	-1.9074320
126	H	5.2971640	0.4237550	-2.7085800
127	H	6.4924990	-0.6874660	-2.0051640
128	H	5.3949130	0.2137650	-0.9475280
129	C	-4.5986210	-4.2443530	-1.4396900
130	H	-3.8731210	-4.6727930	-2.1329870
131	H	-5.4747510	-4.8933870	-1.3816350
132	H	-4.1453870	-4.1744540	-0.4466240
133	C	0.9392710	5.3295510	-0.4591930
134	C	4.6687650	-2.5788720	-1.0222630
135	C	-6.0915440	-2.2102070	-1.0630820
136	O	-6.2119500	-0.8929300	-1.0959250
137	O	4.2022870	-3.7691900	-1.3577090
138	O	2.0309660	4.6455700	-0.7502310
139	N	-6.8489220	-3.0148680	-0.3350820
140	N	5.2683180	-2.3117770	0.1304360
141	N	0.9257710	6.4984660	0.1621760
142	N	-7.8075710	-2.2704540	0.3933400
143	N	2.2409640	6.8927490	0.5053240
144	N	5.3018720	-3.4711540	0.9460870
145	C	5.8794990	-3.3601810	2.1130740
146	H	6.3105520	-2.3898960	2.3620210
147	C	2.3735630	8.0487370	1.1011380
148	H	1.4647450	8.6313720	1.2538360
149	C	-8.6150570	-2.9505160	1.1646960
150	H	-8.4855040	-4.0330760	1.1791700
151	C	3.6105550	8.6041130	1.5803660
152	C	4.8653880	7.9114330	1.5111910
153	C	3.5634700	9.8938340	2.1713630
154	C	6.0112630	8.5568680	2.0428160
155	C	4.6997700	10.5054600	2.6807440
156	H	2.6043800	10.4031170	2.2189740
157	C	5.9319890	9.8219320	2.6116680
158	H	6.9554550	8.0256470	1.9887680
159	H	4.6419460	11.4920480	3.1268260
160	H	6.8295820	10.2876380	3.0078310
161	C	5.9800900	-4.4069390	3.0946080
162	C	5.4056100	-5.7106970	2.9213440
163	C	6.6693670	-4.1097260	4.2993780
164	C	5.5545220	-6.6484060	3.9752590
165	C	6.8050110	-5.0462370	5.3138590
166	H	7.0959800	-3.1168480	4.4159200
167	C	6.2375960	-6.3262890	5.1413530
168	H	5.1153190	-7.6304910	3.8367430
169	H	7.3367160	-4.7982080	6.2256910
170	H	6.3341620	-7.0692580	5.9275790
171	C	-9.6500290	-2.3908980	1.9899150
172	C	-9.9146520	-0.9831860	2.0767180
173	C	-10.436564	-3.2920550	2.7545710
174	C	-10.959930	-0.5548050	2.9350160
175	C	-11.455293	-2.8479420	3.5845120
176	H	-10.223096	-4.3549040	2.6764950
177	C	-11.711618	-1.4629440	3.6697770
178	H	-11.152480	0.5109110	2.9964390
179	H	-12.044945	-3.5530220	4.1596600
180	H	-12.505426	-1.1009770	4.3165390
181	O	5.0088520	6.6886980	0.9826130
182	O	4.7322780	-6.0867570	1.8255910
183	O	-9.2293250	-0.0573660	1.3939340

(Λ, *S*)-**9a** in methanol
Total energy = -12998.609380 Hartrees
Number of negative eigenvalues: 0

No	Symbol	X	Y	Z
1	C	0.3045490	0.8292910	2.3155760
2	C	1.5661280	0.2170150	2.3261500
3	C	1.6810030	-1.1625900	2.1142700
4	C	0.5396980	-1.9382900	1.8707630
5	C	-0.7171620	-1.3206540	1.8714400
6	C	-0.8447510	0.0553670	2.1149390
7	C	2.7824800	1.0691110	2.4522660
8	O	2.8379070	2.2113280	1.8983700
9	N	3.8245540	0.5655140	3.1458330
10	C	5.1261060	1.2467060	3.1905300
11	C	5.8869130	0.7994780	4.4711110
12	C	7.1115330	1.6411490	4.7732970
13	C	8.3645820	1.3271170	4.2169300
14	C	9.4850810	2.1198710	4.4953230
15	C	9.3705830	3.2355330	5.3347330
16	C	8.1269290	3.5560540	5.8942610
17	C	7.0073280	2.7631000	5.6150160
18	C	5.9180750	0.9274570	1.9346750
19	O	6.0941560	-0.3382740	1.6263700
20	N	6.4329270	1.9455050	1.2519430
21	N	7.3247070	1.4521150	0.2582550
22	C	7.9191450	2.3341700	-0.5010420
23	C	8.8842500	2.0363640	-1.5227980
24	C	9.3433640	0.7092430	-1.8199960
25	C	10.3235490	0.5561470	-2.8333520
26	C	10.8228890	1.6496130	-3.5290050
27	C	10.3697490	2.9552390	-3.2438210

28	C	9.4177920	3.1328060	-2.2520190
29	O	8.9061380	-0.3902920	-1.1883010
30	Ga	7.4786660	-0.6042330	0.1321010
31	C	0.7196280	-3.3872260	1.5286680
32	O	1.8607260	-3.8439030	1.1948850
33	N	-0.3835260	-4.1540330	1.5588230
34	C	-0.4968250	-5.4992420	0.9970520
35	C	-0.1144510	-6.6251700	2.0067840
36	C	-0.9560950	-6.6692140	3.2656180
37	C	-0.5805620	-5.9388440	4.4078910
38	C	-1.3584320	-5.9754660	5.5716310
39	C	-2.5258690	-6.7482220	5.6125300
40	C	-2.9080740	-7.4844480	4.4835600
41	C	-2.1293060	-7.4449980	3.3207770
42	C	-1.9269660	-5.6286470	0.4760810
43	O	-2.7733790	-4.6500440	0.7530020
44	N	-2.2302270	-6.7126620	-0.2134220
45	N	-3.5899350	-6.6680180	-0.6181100
46	C	-4.0411460	-7.7012360	-1.2798700
47	C	-5.3777640	-7.8460800	-1.7878890
48	C	-6.3843230	-6.8313020	-1.6649410
49	C	-7.6687770	-7.0989680	-2.2028260
50	C	-7.9506880	-8.3012140	-2.8396630
51	C	-6.9608870	-9.2981780	-2.9693190
52	C	-5.6979300	-9.0622070	-2.4472280
53	O	-6.1742300	-5.6436160	-1.0781920
54	Ga	-4.6205100	-4.9701770	-0.0907970
55	C	-2.2306170	0.6179340	2.0870190
56	O	-3.1745390	-0.0523160	1.5634670
57	N	-2.4550650	1.8299070	2.6440160
58	C	-3.8033280	2.4330920	2.6799360
59	C	-4.0110960	3.1572990	4.0318740
60	C	-5.4560900	3.5474820	4.2845870
61	C	-5.8761690	4.8834950	4.1769800
62	C	-7.2115100	5.2354280	4.4133110
63	C	-8.1469380	4.2536960	4.7608430
64	C	-7.7389510	2.9173610	4.8742450
65	C	-6.4040320	2.5691790	4.6398500
66	C	-3.9918280	3.3543760	1.4822830
67	O	-3.3913540	4.5220600	1.4951370
68	N	-4.7407000	2.9038060	0.4780650
69	N	-4.8114310	3.8803940	-0.5535220
70	C	-5.5444420	3.6000060	-1.5990170
71	C	-5.7375260	4.4518420	-2.7396520
72	C	-5.1325160	5.7462780	-2.8762760
73	C	-5.3987220	6.4840640	-4.0580520
74	C	-6.2207240	5.9785400	-5.0566640
75	C	-6.8196740	4.7072270	-4.9248830
76	C	-6.5743070	3.9656310	-3.7798470
77	O	-4.3319630	6.2918350	-1.9516580
78	Ga	-3.7976620	5.6357660	-0.1865840
79	C	-0.4595950	1.4875080	-1.9440270
80	C	-0.7912910	0.1444810	-2.1823940
81	C	0.2286020	-0.8026760	-2.3283200
82	C	1.5707120	-0.3985110	-2.2845810
83	C	1.8930560	0.9479940	-2.0748460
84	C	0.8782660	1.8975250	-1.8947830
85	C	-2.2457990	-0.1997330	-2.2085910
86	O	-3.0942750	0.5998770	-1.7043020
87	N	-2.6326240	-1.3576480	-2.7914040
88	C	-4.0554060	-1.7460930	-2.8744160
89	C	-4.3353240	-2.4187980	-4.2420660
90	C	-5.8155850	-2.5062790	-4.5672020
91	C	-6.6138580	-3.5486720	-4.0620050
92	C	-7.9813920	-3.6049250	-4.3591840
93	C	-8.5719970	-2.6223240	-5.1641780
94	C	-7.7857320	-1.5801010	-5.6719100
95	C	-6.4186270	-1.5249270	-5.3750040
96	C	-4.4153230	-2.6443880	-1.6993590
97	O	-4.0043360	-3.8916290	-1.7269210
98	N	-5.1171770	-2.1059100	-0.7054090
99	N	-5.3765670	-3.0873270	0.2916530
100	C	-6.1115800	-2.7285120	1.3116260
101	C	-6.4819850	-3.5723640	2.4135210
102	C	-6.0733430	-4.9425500	2.5372470
103	C	-6.5022770	-5.6647470	3.6799980
104	C	-7.2971040	-5.0733180	4.6529690
105	C	-7.7038940	-3.7269830	4.5334620
106	C	-7.2959670	-2.9986770	3.4269920
107	O	-5.3098800	-5.5736830	1.6358380
108	C	2.6374340	-1.4364400	-2.3602470
109	O	2.4834160	-2.5695800	-1.8060790
110	N	3.7729790	-1.1138540	-3.0137130
111	C	4.9415260	-2.0050320	-3.0222250

112	C	5.8087060	-1.6945740	-4.2751130
113	C	6.8676230	-2.7453500	-4.5484750
114	C	8.1495210	-2.6484160	-3.9788210
115	C	9.1141310	-3.6335310	-4.2247960
116	C	8.8119380	-4.7283090	-5.0447460
117	C	7.5379730	-4.8331080	-5.6183570
118	C	6.5747010	-3.8475980	-5.3717500
119	C	5.7349750	-1.8304100	-1.7386000
120	O	6.1261970	-0.6180080	-1.4177070
121	N	6.0292080	-2.9235910	-1.0408140
122	N	6.9546840	-2.5982520	-0.0093790
123	C	7.3418330	-3.5702530	0.7737610
124	C	8.2998890	-3.4504040	1.8377130
125	C	8.9866880	-2.2306260	2.1556510
126	C	9.9320950	-2.2561840	3.2123620
127	C	10.1851050	-3.4182910	3.9297360
128	C	9.5073020	-4.6177310	3.6244170
129	C	8.5842440	-4.6214850	2.5903730
130	O	8.7918190	-1.0735700	1.5052540
131	C	1.2654040	3.3079950	-1.5646640
132	O	2.4032630	3.5692100	-1.0642320
133	N	0.3355870	4.2524280	-1.8043500
134	C	0.3544190	5.6246360	-1.2991610
135	C	0.7116180	6.6695480	-2.4021160
136	C	2.1125760	6.5235370	-2.9566930
137	C	3.2048510	7.1246110	-2.3039380
138	C	4.5059950	6.9759160	-2.7983160
139	C	4.7355060	6.2244210	-3.9595870
140	C	3.6547400	5.6295930	-4.6234140
141	C	2.3541550	5.7809280	-4.1255700
142	C	-1.0361440	5.9084130	-0.7287160
143	O	-1.9947790	5.0297580	-0.9710650
144	N	-1.1988980	7.0384840	-0.0648360
145	N	-2.5514480	7.1764270	0.3414910
146	C	-2.8594990	8.2567660	1.0102990
147	C	-4.1669650	8.5830460	1.5099430
148	C	-5.3113510	7.7327070	1.3464450
149	C	-6.5495310	8.1727140	1.8811560
150	C	-6.6597300	9.3873470	2.5468710
151	C	-5.5357760	10.2242100	2.7120340
152	C	-4.3141100	9.8169130	2.1974640
153	O	-5.2747460	6.5508270	0.7160940
154	H	5.1794260	0.8687370	5.3054220
155	H	6.1641670	-0.2550500	4.3562200
156	H	8.4626640	0.4686340	3.5586370
157	H	6.0466400	3.0119210	6.0582500
158	H	10.4458010	1.8645470	4.0586720
159	H	8.0294800	4.4159990	6.5495920
160	H	10.2407020	3.8468010	5.5524770
161	H	4.9453110	2.3228660	3.2300320
162	H	-0.1944700	-7.5723220	1.4635230
163	H	0.9405040	-6.4777770	2.2630070
164	H	0.3298470	-5.3467650	4.3872920
165	H	-2.4286730	-8.0192060	2.4494520
166	H	-1.0507290	-5.4076810	6.4443410
167	H	-3.8065480	-8.0931980	4.5096640
168	H	-3.1274870	-6.7813080	6.5153470
169	H	0.1953150	-5.5755570	0.1514200
170	H	-3.3709120	4.0451360	4.0590670
171	H	-3.6701080	2.4741140	4.8190950
172	H	-5.1565170	5.6491970	3.9063600
173	H	-6.0923360	1.5329730	4.7436100
174	H	-7.5159380	6.2732930	4.3242320
175	H	-8.4560740	2.1509110	5.1512760
176	H	-9.1813920	4.5255100	4.9454030
177	H	-4.5137430	1.6097930	2.5904570
178	H	-3.8291960	-1.8225300	-5.0090460
179	H	-3.8818960	-3.4160250	-4.2381890
180	H	-6.1677460	-4.3099690	-3.4300920
181	H	-5.8117830	-0.7186670	-5.7785320
182	H	-8.5821320	-4.4191340	-3.9660810
183	H	-8.2327770	-0.8164020	-6.3007800
184	H	-9.6313190	-2.6699490	-5.3960340
185	H	-4.6347870	-0.8252650	-2.7932330
186	H	5.1264560	-1.6298290	-5.1305630
187	H	6.2706590	-0.7094220	-4.1413800
188	H	8.3895280	-1.8077970	-3.3346380
189	H	5.5906280	-3.9301640	-5.8255860
190	H	10.0996320	-3.5445680	-3.7779700
191	H	7.2967970	-5.6753260	-6.2594120
192	H	9.5611950	-5.4896260	-5.2375130
193	H	4.5818430	-3.0346510	-3.0771760
194	H	0.5807100	7.6570650	-1.9478150
195	H	-0.0287120	6.5787050	-3.2057620

196	H	3.0333260	7.7164930	-1.4086000
197	H	1.5195080	5.3234620	-4.6487770
198	H	5.3360550	7.4532410	-2.2863750
199	H	3.8221290	5.0558820	-5.5297940
200	H	5.7430020	6.1142860	-4.3481420
201	H	1.0992120	5.6843040	-0.5009970
202	H	0.2574170	1.9060810	2.4312850
203	H	2.6519010	-1.6427490	2.0771130
204	H	-1.6268190	-1.8631420	1.6448430
205	H	7.6678590	3.3831010	-0.3380170
206	H	10.6729650	-0.4480380	-3.0462880
207	H	11.5714860	1.4957780	-4.3003210
208	H	10.7633880	3.8054880	-3.7890730
209	H	9.0621820	4.1314690	-2.0122970
210	H	-3.3416960	-8.5188460	-1.4553570
211	H	-8.4272760	-6.3310800	-2.0957820
212	H	-8.9450590	-8.4738350	-3.2403440
213	H	-7.1852120	-10.234268	-3.4681010
214	H	-6.0569600	2.6376250	-1.6097190
215	H	-4.9376280	7.4610490	-4.1536470
216	H	-6.4044160	6.5703110	-5.9483930
217	H	-7.4617070	4.3181190	-5.7069390
218	H	-7.0276640	2.9850110	-3.6598900
219	H	-1.2757420	2.1748280	-1.7548860
220	H	0.0160460	-1.8592730	-2.4431950
221	H	2.9253870	1.2692420	-1.9953600
222	H	-6.4793370	-1.7019910	1.3293780
223	H	-6.1865960	-6.6984230	3.7661980
224	H	-7.6092270	-5.6553360	5.5149180
225	H	-8.3255480	-3.2707330	5.2954490
226	H	-7.6000480	-1.9609650	3.3169500
227	H	6.9075390	-4.5554820	0.5983280
228	H	10.4532410	-1.3336910	3.4429330
229	H	10.9138200	-3.4013720	4.7345250
230	H	9.7088540	-5.5220700	4.1871930
231	H	8.0580250	-5.5374980	2.3348340
232	H	-2.0537570	8.9663640	1.1991740
233	H	-7.4097320	7.5256640	1.7496230
234	H	-7.6231420	9.6949620	2.9423820
235	H	-5.6267710	11.1708960	3.2325020
236	H	-3.4374840	10.4487400	2.3137830
237	H	-0.5629170	3.9783500	-2.1825460
238	H	-1.7036700	2.3378360	3.0895480
239	H	-1.9541730	-1.9690760	-3.2238560
240	H	-1.2789040	-3.7599530	1.8234230
241	H	3.8499270	-0.2226510	-3.4843530
242	H	3.7387310	-0.3283480	3.6101430
243	H	-4.9230640	-9.8191350	-2.5349540
244	Na	-4.6809730	0.3837890	-0.0936500
245	Na	3.5771740	-3.8630280	-0.2818890
246	Na	4.1382630	3.2470710	0.3340390

(Δ, *S*)-**9a** in methanol
Total energy = -12998.571040 Hartrees
Number of negative eigenvalues: 0

No	Symbol	X	Y	Z
1	C	-0.3902760	1.4105650	2.1703590
2	C	-1.6695420	0.8353460	2.2057600
3	C	-1.8157700	-0.5572350	2.2371420
4	C	-0.6904560	-1.3914670	2.2033810
5	C	0.5817880	-0.8092380	2.2150000
6	C	0.7415590	0.5847660	2.2069670
7	C	-2.8855810	1.6953390	2.0795660
8	O	-2.9495020	2.6072100	1.1983210
9	N	-3.9102350	1.4127620	2.9106520
10	C	-5.2536310	2.0225230	2.8222400
11	C	-5.2679870	3.5448030	3.1039990
12	C	-4.7358320	3.8889530	4.4827830
13	C	-3.4489840	4.4314440	4.6410770
14	C	-2.9521040	4.7417420	5.9134780
15	C	-3.7377850	4.5123680	7.0495230
16	C	-5.0238590	3.9743540	6.9046600
17	C	-5.5176000	3.6672200	5.6317120
18	C	-5.9479530	1.5840250	1.5338610
19	O	-6.1565790	0.2920350	1.3978530
20	N	-6.3627400	2.5024180	0.6659870
21	N	-7.1850680	1.8973820	-0.3268760
22	C	-7.6632670	2.6764530	-1.2629560
23	C	-8.5412350	2.2643690	-2.3218030
24	C	-9.0531040	0.9292810	-2.4523950
25	C	-9.9255370	0.6542470	-3.5363560
26	C	-10.277206	1.6394370	-4.4494320
27	C	-9.7764860	2.9533960	-4.3240760
28	C	-8.9251140	3.2494090	-3.2713190
29	O	-8.7623610	-0.0628090	-1.6014660
30	Ga	-7.5013880	-0.1203510	-0.1044390
31	C	-0.9084980	-2.8713840	2.0704800
32	O	-2.0746890	-3.3740160	2.1852500
33	N	0.1712730	-3.6095570	1.7744660
34	C	0.1977660	-5.0534550	1.5278520
35	C	-0.5556340	-5.4279720	0.2036760
36	C	-1.2025980	-6.7992010	0.2155640
37	C	-2.2487550	-7.0845260	1.1149340
38	C	-2.8748720	-8.3364550	1.1123100
39	C	-2.4678840	-9.3248590	0.2057400
40	C	-1.4300580	-9.0519170	-0.6938210
41	C	-0.8026070	-7.7995010	-0.6859530
42	C	1.6800370	-5.4195810	1.4432030
43	O	2.5473580	-4.4187060	1.4882070
44	N	2.0082640	-6.6838920	1.2626580
45	N	3.4080210	-6.7929120	1.0449990
46	C	3.8933520	-7.9969200	0.8938690
47	C	5.2665620	-8.3075210	0.6009330
48	C	6.2704850	-7.3108520	0.3583980
49	C	7.5822600	-7.7490500	0.0472700
50	C	7.8982410	-9.1003890	-0.0169710
51	C	6.9148110	-10.083054	0.2228520
52	C	5.6221740	-9.6799690	0.5228390
53	O	6.0335730	-5.9905490	0.3960740
54	Ga	4.4484640	-5.0257410	1.0101220
55	C	2.1523870	1.0889110	2.1468880
56	O	3.0614480	0.3232670	1.7034070
57	N	2.4126090	2.3416580	2.5804280
58	C	3.7400150	3.0098660	2.5542040
59	C	4.8735130	2.2146850	3.2513250
60	C	4.5734820	1.9057380	4.7061460
61	C	4.1673500	0.6191380	5.1004150
62	C	3.8800930	0.3383910	6.4424610
63	C	3.9948410	1.3427860	7.4113540
64	C	4.4021630	2.6287230	7.0308250
65	C	4.6897750	2.9055960	5.6895370
66	C	4.0909130	3.4934070	1.1464200
67	O	3.6934570	4.7144180	0.8574180
68	N	4.7658820	2.7121150	0.3061730
69	N	4.9715590	3.3975520	-0.9262850
70	C	5.7772090	2.8482190	-1.7972920
71	C	6.0613700	3.3615810	-3.1089710
72	C	5.4312750	4.5304280	-3.6534430
73	C	5.7724410	4.9170580	-4.9741510
74	C	6.6938870	4.1964520	-5.7228950
75	C	7.3185230	3.0485770	-5.1897300
76	C	6.9969080	2.6456320	-3.9029200
77	O	4.5296500	5.2636910	-2.9856320
78	Ga	4.0689810	5.2428270	-1.0840690
79	C	0.4194070	0.7977470	-2.1082700
80	C	0.6728750	-0.5815770	-2.1088410
81	C	-0.4006660	-1.4815760	-2.0527210
82	C	-1.7153080	-0.9917620	-2.0663600
83	C	-1.9565720	0.3885710	-2.0921950
84	C	-0.8878150	1.2957490	-2.0719820
85	C	2.1167150	-0.9864520	-2.0901180
86	O	2.9829740	-0.1639100	-1.6627100
87	N	2.4516420	-2.2119850	-2.5489430
88	C	3.8244160	-2.7806240	-2.5774240
89	C	4.8677810	-1.8965600	-3.3069880
90	C	4.4916900	-1.5996240	-4.7466160
91	C	3.9908080	-0.3387820	-5.1141340
92	C	3.6369040	-0.0674970	-6.4421500
93	C	3.7778040	-1.0562810	-7.4235370
94	C	4.2781080	-2.3167810	-7.0695680
95	C	4.6329990	-2.5838150	-5.7425060
96	C	4.2602570	-3.2504160	-1.1889230
97	O	3.9597160	-4.4997390	-0.9034290
98	N	4.9088610	-2.4333010	-0.3621040
99	N	5.2067630	-3.1169290	0.8526520

100	C	5.9834540	-2.5135260	1.7139750
101	C	6.3465780	-3.0213290	3.0084600
102	C	5.8412380	-4.2521360	3.5460820
103	C	6.2535250	-4.6291530	4.8491420
104	C	7.1251660	-3.8399200	5.5882220
105	C	7.6261970	-2.6297650	5.0624520
106	C	7.2336900	-2.2360010	3.7925410
107	O	4.9928110	-5.0546540	2.8883270
108	C	-2.8761760	-1.9277200	-1.9347430
109	O	-2.9319860	-2.7687510	-0.9845090
110	N	-3.8572190	-1.7894730	-2.8478330
111	C	-5.1686090	-2.4648830	-2.7800280
112	C	-5.0835680	-4.0047590	-2.9170710
113	C	-4.4503860	-4.4465070	-4.2228280
114	C	-3.1178520	-4.8920730	-4.2614960
115	C	-2.5293620	-5.2927220	-5.4678080
116	C	-3.2676350	-5.2525310	-6.6570160
117	C	-4.5981390	-4.8129900	-6.6313690
118	C	-5.1833110	-4.4154940	-5.4237840
119	C	-5.9488140	-1.9606660	-1.5688120
120	O	-6.0091770	-0.6520750	-1.4074030
121	N	-6.5869850	-2.8371220	-0.8054100
122	N	-7.4215570	-2.1571550	0.1207240
123	C	-8.0533830	-2.8837730	1.0051520
124	C	-8.9789790	-2.3816570	1.9828670
125	C	-9.3816020	-1.0054280	2.0592830
126	C	-10.318736	-0.6411730	3.0593500
127	C	-10.835148	-1.5794020	3.9436720
128	C	-10.442619	-2.9330890	3.8710230
129	C	-9.5305670	-3.3161210	2.8997030
130	O	-8.9341860	-0.0531650	1.2293350
131	C	-1.1921940	2.7599140	-1.9266520
132	O	-2.3906630	3.1901690	-1.9855740
133	N	-0.1452800	3.5621140	-1.6800540
134	C	-0.1913720	4.9978510	-1.3948830
135	C	-0.8585340	5.2809140	-0.0033280
136	C	-1.5440370	6.6280110	0.1045090
137	C	-2.6588090	6.9260410	-0.7033760
138	C	-3.3201660	8.1542760	-0.5882330
139	C	-2.8799460	9.1058130	0.3419740
140	C	-1.7740440	8.8197370	1.1518940
141	C	-1.1118150	7.5913220	1.0310680
142	C	1.2657970	5.4579740	-1.4003880
143	O	2.1913950	4.5113900	-1.4655220
144	N	1.5215810	6.7441130	-1.2618370
145	N	2.9200170	6.9429980	-1.1051040
146	C	3.3349090	8.1775340	-0.9964400
147	C	4.6967830	8.5784920	-0.7676080
148	C	5.7703520	7.6507860	-0.5519870
149	C	7.0641300	8.1751490	-0.3056910
150	C	7.2970490	9.5445160	-0.2775020
151	C	6.2446210	10.4596020	-0.4903790
152	C	4.9683530	9.9715660	-0.7277600
153	O	5.6146710	6.3178680	-0.5569240
154	H	-6.3080910	3.8738400	3.0069930
155	H	-4.6919110	4.0695160	2.3405740
156	H	-2.8368890	4.6163020	3.7625450
157	H	-6.5200090	3.2596070	5.5282540
158	H	-1.9567930	5.1629800	6.0160190
159	H	-5.6421330	3.8000380	7.7798110
160	H	-3.3553180	4.7537890	8.0361510
161	H	-5.8270310	1.5335620	3.6178530
162	H	0.1460590	-5.3501320	-0.6332490
163	H	-1.3244690	-4.6659440	0.0442790
164	H	-2.5708650	-6.3225930	1.8200040
165	H	0.0063830	-7.5962170	-1.3810630
166	H	-3.6785070	-8.5394630	1.8137250
167	H	-1.1070860	-9.8118830	-1.3985970
168	H	-2.9536730	-10.295428	0.2016670
169	H	-0.2722420	-5.5763970	2.3676050
170	H	5.0732630	1.2957290	2.7010780
171	H	5.7721470	2.8398410	3.1808050
172	H	4.0787350	-0.1630250	4.3521850
173	H	5.0142220	3.9030520	5.4039840
174	H	3.5707070	-0.6618340	6.7296060
175	H	4.5007230	3.4112500	7.7768450
176	H	3.7742780	1.1259250	8.4517640
177	H	3.5839330	3.9302300	3.1224700
178	H	5.8138930	-2.4508290	-3.2751880
179	H	5.0154760	-0.9686160	-2.7555980
180	H	3.8816330	0.4317320	-4.3565030
181	H	5.0305420	-3.5606060	-5.4787670
182	H	3.2553820	0.9133460	-6.7085440
183	H	4.3970710	-3.0867460	-7.8256150
184	H	3.5054540	-0.8468870	-8.4531430
185	H	3.7155050	-3.7056130	-3.1491360
186	H	-6.1073070	-4.3847960	-2.8443600
187	H	-4.5273300	-4.4144120	-2.0719270
188	H	-2.5426150	-4.9293160	-3.3405410
189	H	-6.2191300	-4.0859640	-5.4112550
190	H	-1.4998140	-5.6371200	-5.4779450
191	H	-5.1796030	-4.7857390	-7.5477410
192	H	-2.8139200	-5.5643730	-7.5924160
193	H	-5.7171300	-2.0848340	-3.6508810
194	H	-1.5881960	4.4850190	0.1719330
195	H	-0.0939050	5.1837710	0.7750440
196	H	-3.0080050	6.1926740	-1.4259020
197	H	-0.2506640	7.3784800	1.6574470
198	H	-4.1768480	8.3673780	-1.2204450
199	H	-1.4250620	9.5511400	1.8742850
200	H	-3.3929470	10.0578790	0.4335360
201	H	-0.7560480	5.5130650	-2.1783820
202	H	-0.3037310	2.4880490	2.0858240
203	H	-2.8011920	-1.0064650	2.2261020
204	H	1.4879460	-1.4012140	2.2183640
205	H	-7.3720110	3.7275530	-1.2332290
206	H	-10.309321	-0.3560550	-3.6265040
207	H	-10.946349	1.3946680	-5.2687960
208	H	-10.056207	3.7186910	-5.0390780
209	H	-8.5340070	4.2568100	-3.1550190
210	H	3.1943340	-8.8282620	0.9872900
211	H	8.3336680	-6.9890100	-0.1374020
212	H	8.9140870	-9.4013720	-0.2549730
213	H	7.1656760	-11.136516	0.1710290
214	H	6.2760700	1.9215230	-1.5096020
215	H	5.2899300	5.7999530	-5.3789410
216	H	6.9356070	4.5217230	-6.7302740
217	H	8.0367280	2.4899110	-5.7790100
218	H	7.4651160	1.7619640	-3.4770740
219	H	1.2857570	1.4462030	-2.1249220
220	H	-0.2392120	-2.5509630	-1.9737000
221	H	-2.9716860	0.7675140	-2.0701440
222	H	6.3921490	-1.5421270	1.4326560
223	H	5.8657240	-5.5602010	5.2478340
224	H	7.4236430	-4.1593920	6.5821460
225	H	8.3057210	-2.0172660	5.6440560
226	H	7.6064980	-1.3054120	3.3723470
227	H	-7.8640510	-3.9579270	0.9936560
228	H	-10.619016	0.3997960	3.1090990
229	H	-11.549825	-1.2659060	4.6986860
230	H	-10.850445	-3.6617130	4.5624390
231	H	-9.2207060	-4.3551380	2.8242060
232	H	2.5820650	8.9620690	-1.0750270
233	H	7.8689620	7.4668770	-0.1414920
234	H	8.3011340	9.9125050	-0.0889270
235	H	6.4309050	11.5273740	-0.4666510
236	H	4.1440550	10.6609840	-0.8899080
237	H	0.8038580	3.2017010	-1.6385710
238	H	1.6626670	2.8725260	3.0006700
239	H	1.7292140	-2.7891990	-2.9556800
240	H	1.0946480	-3.1937850	1.6862730
241	H	-3.7146830	-1.1351740	-3.6042830
242	H	-3.7637150	0.7257760	3.6365400
243	H	4.8500780	-10.422463	0.7063420
244	Na	4.4630760	0.1380590	-0.0251820
245	Na	-3.9289040	-3.5589230	0.8784520
246	Na	-4.1693680	3.3341580	-0.5743710

(Λ, *S*)-**10b** in methanol
Total energy = -4180.472547 Hartrees
Number of negative eigenvalues: 0

No	Symbol	X	Y	Z
1	Ga	1.7760810	-0.0027260	-0.0000730
2	C	-5.2910050	-6.1387370	0.1107560
3	C	-4.0626360	-6.5089170	-0.4514350

4	C	-3.1576690	-5.5282710	-0.8690170
5	C	-3.4759660	-4.1649850	-0.7246550
6	C	-4.7038100	-3.8004610	-0.1437640
7	C	-5.6088580	-4.7824930	0.2653190
8	H	-5.9925870	-6.9014420	0.4319140
9	H	-2.1967760	-5.8326160	-1.2710760
10	H	-4.9392950	-2.7489920	-0.0261340
11	C	-2.5432330	-3.0796050	-1.1437960
12	N	-1.6679120	-3.3484170	-2.1392420
13	O	-2.5757400	-1.9378580	-0.5822340
14	H	-1.7272460	-4.2360090	-2.6171200
15	C	-0.6609120	-2.3884260	-2.6447070
16	H	0.0128530	-2.9945560	-3.2591700
17	C	-1.2921330	-1.3071300	-3.5319970
18	H	-0.5183610	-0.6431870	-3.9264390
19	H	-1.8071490	-1.7821010	-4.3725360
20	H	-2.0122150	-0.7042680	-2.9742470
21	C	0.1955550	-1.8260330	-1.5059620
22	O	0.2428230	-0.5113520	-1.3139100
23	N	0.8786190	-2.6994780	-0.7887570
24	N	1.6720700	-2.0384020	0.1830950
25	C	2.3013970	-2.7959430	1.0437880
26	H	2.1357430	-3.8707730	0.9680190
27	C	3.1886690	-2.3280340	2.0718750
28	C	3.5440100	-0.9484670	2.2395260
29	C	3.7434860	-3.2971300	2.9493210
30	C	4.4380720	-0.6122960	3.2866250
31	C	4.6151440	-2.9429060	3.9678370
32	H	3.4674610	-4.3385150	2.8069950
33	C	4.9599100	-1.5843940	4.1308900
34	H	4.7017430	0.4329290	3.4058050
35	H	5.0266060	-3.6975370	4.6285400
36	H	5.6417070	-1.2933220	4.9242940
37	O	3.0852290	0.0361750	1.4516870
38	C	-4.6894320	3.8144700	0.1432960
39	C	-5.5924580	4.7983320	-0.2658490
40	C	-5.2718970	6.1539230	-0.1111680
41	C	-4.0428470	6.5216020	0.4511770
42	C	-3.1398890	5.5391200	0.8688070
43	C	-3.4609210	4.1764920	0.7243480
44	H	-4.9269980	2.7634800	0.0255750
45	H	-5.9719060	6.9180600	-0.4323560
46	H	-2.1784240	5.8415170	1.2709590
47	C	-2.5305580	3.0891290	1.1436130
48	N	-1.6547310	3.3559570	2.1391480
49	O	-2.5655020	1.9474450	0.5821020
50	H	-1.7121500	4.2436050	2.6171710
51	C	-0.6504810	2.3933250	2.6451070
52	H	0.0254130	2.9978940	3.2587550
53	C	-1.2848220	1.3148120	3.5335570
54	H	-0.5130600	0.6486960	3.9282830
55	H	-1.7980270	1.7921120	4.3738820
56	H	-2.0070620	0.7137890	2.9765890
57	C	0.2037450	1.8271670	1.5065560
58	O	0.2462500	0.5122330	1.3152790
59	N	0.8899430	2.6977400	0.7888660
60	N	1.6805020	2.0333490	-0.1830750
61	C	2.3129620	2.7883160	-1.0437440
62	H	2.1520760	3.8638470	-0.9676440
63	C	3.1978620	2.3167740	-2.0722080
64	C	3.5466070	0.9356360	-2.2407230
65	C	3.7570660	3.2837160	-2.9492550
66	C	4.4388500	0.5958090	-3.2882010
67	C	4.6268330	2.9259350	-3.9681370
68	H	3.4859950	4.3263120	-2.8062940
69	C	4.9651250	1.5658900	-4.1320210
70	H	4.6974940	-0.4506000	-3.4080320
71	H	5.0417250	3.6789750	-4.6285130
72	H	5.6453750	1.2720390	-4.9257290
73	O	3.0831690	-0.0473140	-1.4535000
74	H	-6.5580170	-4.4924810	0.7031180
75	H	-3.8054500	-7.5573140	-0.5569880
76	H	-6.5421440	4.5102450	-0.7037750
77	H	-3.7835690	7.5694750	0.5568090
78	Na	-1.6614680	0.0016390	0.0028550

(Δ, *S*)-**10b** in methanol
Total energy = -4180.479073 Hartrees
Number of negative eigenvalues: 0

No	Symbol	X	Y	Z
1	Ga	1.6931740	-0.0741580	-0.0023710
2	C	-5.5249400	-6.0353170	-0.3862400
3	C	-4.3107390	-6.4142210	0.2005780
4	C	-3.4197930	-5.4405670	0.6617600
5	C	-3.7380360	-4.0752220	0.5371400
6	C	-4.9511440	-3.7015960	-0.0684750
7	C	-5.8422870	-4.6769670	-0.5214870
8	H	-6.2157320	-6.7929040	-0.7410940
9	H	-2.4698390	-5.7526400	1.0834130
10	H	-5.1861960	-2.6484230	-0.1699580
11	C	-2.8235580	-2.9977340	1.0075150
12	N	-1.9491430	-3.2843140	1.9985200
13	O	-2.8652810	-1.8293660	0.5006030
14	H	-1.9618730	-4.1940090	2.4369070
15	C	-0.9972350	-2.2879760	2.5136890
16	H	-1.5597880	-1.3996360	2.8204860
17	C	-0.2538370	-2.8724260	3.7254590
18	H	-0.9621380	-3.1455580	4.5144960
19	H	0.4397640	-2.1309190	4.1311960
20	H	0.3205970	-3.7571360	3.4332220
21	C	-0.0255910	-1.8209960	1.4343900
22	O	0.1241740	-0.5057760	1.2859980
23	N	0.6180370	-2.7272410	0.7263410
24	N	1.4984960	-2.1051170	-0.1968250
25	C	2.1302080	-2.8931990	-1.0275210
26	H	1.9131670	-3.9592090	-0.9575730
27	C	3.0799530	-2.4688900	-2.0176360
28	C	3.4830740	-1.1030500	-2.1903800
29	C	3.6430880	-3.4683430	-2.8549470
30	C	4.4320840	-0.8110050	-3.2019680
31	C	4.5697890	-3.1570810	-3.8381220
32	H	3.3290740	-4.4984920	-2.7093780
33	C	4.9619070	-1.8119950	-4.0063810
34	H	4.7316010	0.2239700	-3.3254960
35	H	4.9875160	-3.9343490	-4.4679110
36	H	5.6869520	-1.5543950	-4.7725460
37	O	3.0170950	-0.0916190	-1.4422350
38	C	-4.5835600	4.1169700	0.0618690
39	C	-5.3932330	5.1576010	0.5223570
40	C	-4.9667590	6.4867810	0.3980300
41	C	-3.7252850	6.7706690	-0.1853350
42	C	-2.9155920	5.7316920	-0.6539230
43	C	-3.3435270	4.3957530	-0.5404220
44	H	-4.9024430	3.0853430	0.1554540
45	H	-5.5941890	7.2950780	0.7586270
46	H	-1.9429780	5.9693940	-1.0720990
47	C	-2.5206360	3.2497270	-1.0181650
48	N	-1.6200330	3.4693080	-2.0030050
49	O	-2.6655100	2.0846140	-0.5230500
50	H	-1.5520730	4.3807930	-2.4325310
51	C	-0.7570250	2.3988420	-2.5269390
52	H	-1.3945480	1.5670640	-2.8455340
53	C	0.0390950	2.9289560	-3.7301230
54	H	-0.6394840	3.2724830	-4.5177920
55	H	0.6658170	2.1336000	-4.1426500
56	H	0.6884750	3.7562270	-3.4269700
57	C	0.1650410	1.8354140	-1.4501140
58	O	0.1929560	0.5107760	-1.3128250
59	N	0.8863070	2.6727970	-0.7321680
60	N	1.6973490	1.9655690	0.1931750
61	C	2.3972580	2.6869620	1.0299670
62	H	2.2879360	3.7692620	0.9582990
63	C	3.2905320	2.1709380	2.0290780
64	C	3.5493990	0.7714810	2.2093650
65	C	3.9445090	3.1100340	2.8702270
66	C	4.4533990	0.3866420	3.2311250
67	C	4.8245770	2.7083980	3.8634360
68	H	3.7386910	4.1664480	2.7200720
69	C	5.0751200	1.3307010	4.0385410
70	H	4.6431440	-0.6733100	3.3604810
71	H	5.3132980	3.4406650	4.4960720
72	H	5.7621220	1.0026960	4.8128220
73	O	2.9873810	-0.1890540	1.4604960
74	H	-6.7802800	-4.3800810	-0.9782630
75	H	-4.0538310	-7.4639850	0.2920710

76	H	-6.3525880	4.9341120	0.9765850
77	H	-3.3841610	7.7968730	-0.2683660
78	Na	-1.8150510	0.0736780	-0.0364330

(Λ, *S*)-**10b**-Na in methanol

Total energy = -4018.189538 Hartrees

Number of negative eigenvalues: 0

No	Symbol	X	Y	Z
1	Ga	-0.0009770	1.0138450	0.0014510
2	C	-9.2316510	-2.2020320	-0.8270720
3	C	-8.0607590	-2.0419980	-1.5790760
4	C	-6.8108890	-2.0868060	-0.9523740
5	C	-6.7211150	-2.2922110	0.4364540
6	C	-7.9003860	-2.4672950	1.1812760
7	C	-9.1482820	-2.4171630	0.5553950
8	H	-10.200140	-2.1652050	-1.3151860
9	H	-5.9149870	-1.9909710	-1.5568510
10	H	-7.8190700	-2.6390110	2.2484080
11	C	-5.4158460	-2.3565200	1.1691770
12	N	-4.3348090	-1.8048000	0.5615320
13	O	-5.3343690	-2.8929090	2.3143430
14	H	-4.4156560	-1.2498820	-0.2834860
15	C	-3.0049280	-1.7910830	1.1768580
16	H	-3.1162130	-1.5298170	2.2371520
17	C	-2.3088740	-3.1667540	1.0903110
18	H	-1.3224600	-3.1179310	1.5597000
19	H	-2.9141360	-3.9141770	1.6090770
20	H	-2.1892470	-3.4672110	0.0441650
21	C	-2.1636160	-0.7153140	0.5173380
22	O	-0.9755760	-0.4735480	1.0292040
23	N	-2.6658320	-0.1017410	-0.5455810
24	N	-1.7558180	0.8534960	-1.0490600
25	C	-2.1135200	1.5168790	-2.1171480
26	H	-3.0931530	1.2879760	-2.5375220
27	C	-1.3201260	2.5155020	-2.7805390
28	C	-0.0078260	2.9010520	-2.3452090
29	C	-1.8788120	3.1368690	-3.9284460
30	C	0.6749060	3.8954670	-3.0917560
31	C	-1.1907850	4.1080360	-4.6404670
32	H	-2.8732190	2.8330530	-4.2449720
33	C	0.0991860	4.4847230	-4.2101390
34	H	1.6662700	4.1803750	-2.7562260
35	H	-1.6360960	4.5704340	-5.5142160
36	H	0.6498420	5.2434070	-4.7583660
37	O	0.6046310	2.3759380	-1.2760490
38	C	7.9150060	-2.4473580	-1.1841210
39	C	9.1572940	-2.4114190	-0.5462080
40	C	9.2289000	-2.2152030	0.8397270
41	C	8.0518750	-2.0599320	1.5830910
42	C	6.8076550	-2.0906220	0.9444270
43	C	6.7297240	-2.2768850	-0.4478150
44	H	7.8428270	-2.6044690	-2.2541470
45	H	10.1930130	-2.1894320	1.3371250
46	H	5.9066830	-1.9989040	1.5419550
47	C	5.4308210	-2.3256160	-1.1930100
48	N	4.3462010	-1.7796200	-0.5866080
49	O	5.3575970	-2.8447720	-2.3466400
50	H	4.4212220	-1.2373570	0.2671100
51	C	3.0210580	-1.7551520	-1.2117080
52	H	3.1400010	-1.4749210	-2.2663630
53	C	2.3249990	-3.1323410	-1.1548390
54	H	1.3415050	-3.0750040	-1.6293200
55	H	2.9338490	-3.8699080	-1.6833990
56	H	2.1989760	-3.4518520	-0.1151010
57	C	2.1741330	-0.6919130	-0.5391780
58	O	0.9870590	-0.4465650	-1.0515050
59	N	2.6705060	-0.0925740	0.5345690
60	N	1.7547540	0.8507000	1.0500790
61	C	2.1083680	1.5025240	2.1266160
62	H	3.0897900	1.2750530	2.5435820
63	C	1.3084300	2.4870270	2.8031210
64	C	-0.0079920	2.8667900	2.3752530
65	C	1.8646600	3.0996620	3.9569170
66	C	-0.6968670	3.8472200	3.1345310
67	C	1.1705980	4.0572140	4.6814010
68	H	2.8621900	2.8002530	4.2677650
69	C	-0.1232850	4.4284660	4.2581820
70	H	-1.6912370	4.1279600	2.8044380
71	H	1.6142360	4.5132310	5.5593430
72	H	-0.6786700	5.1765830	4.8160750
73	O	-0.6192030	2.3483590	1.3021020
74	H	-10.052367	-2.5456620	1.1414580
75	H	-8.1191730	-1.8899750	-2.6517570
76	H	10.0661510	-2.5362930	-1.1256360
77	H	8.1010880	-1.9226400	2.6582170

(Δ, *S*)-**10b**-Na in methanol

Total energy = -4018.189243 Hartrees

Number of negative eigenvalues: 0

No	Symbol	X	Y	Z
1	Ga	0.0000030	1.2874310	0.0001360
2	C	8.5308470	-3.4169970	-0.8653980
3	C	7.5566380	-2.5622350	-1.3969040
4	C	6.3518020	-2.3590070	-0.7158250
5	C	6.1100020	-3.0119900	0.5067170
6	C	7.0864910	-3.8799260	1.0253000
7	C	8.2919320	-4.0774110	0.3474420
8	H	9.4654560	-3.5712900	-1.3948020
9	H	5.5989350	-1.7160060	-1.1604450
10	H	6.8839870	-4.3894930	1.9602740
11	C	4.8375930	-2.8485560	1.2814430
12	N	4.1030040	-1.7351900	1.0358560
13	O	4.4758790	-3.7124810	2.1350800
14	H	4.3940430	-1.0335380	0.3653360
15	C	2.8038190	-1.4786930	1.6621120
16	H	2.2193200	-2.4070250	1.6415150
17	C	2.9403210	-1.0267140	3.1331860
18	H	3.4419320	-1.8086650	3.7081850
19	H	1.9510190	-0.8453230	3.5622280
20	H	3.5277330	-0.1045420	3.1947570
21	C	2.0591320	-0.4388340	0.8458440
22	O	0.8103530	-0.1875350	1.1763350
23	N	2.7196470	0.1733950	-0.1279740
24	N	1.8966490	1.1258740	-0.7681090
25	C	2.4148200	1.7922150	-1.7661080
26	H	3.4487640	1.5666270	-2.0289540
27	C	1.7318240	2.7910680	-2.5427060
28	C	0.3655930	3.1707550	-2.3190420
29	C	2.4625050	3.4191050	-3.5856510
30	C	-0.1938070	4.1664040	-3.1605680
31	C	1.8922540	4.3909960	-4.3942130
32	H	3.4955430	3.1197030	-3.7421750
33	C	0.5492090	4.7619390	-4.1718850
34	H	-1.2265810	4.4479670	-2.9853420
35	H	2.4685430	4.8582280	-5.1848630
36	H	0.0893460	5.5213620	-4.7973140
37	O	-0.4066550	2.6389860	-1.3623460
38	C	-7.0853270	-3.8816630	-1.0242280
39	C	-8.2908160	-4.0793060	-0.3465040
40	C	-8.5304360	-3.4180110	0.8657130
41	C	-7.5568700	-2.5622140	1.3967300
42	C	-6.3519790	-2.3588510	0.7157880
43	C	-6.1094670	-3.0127200	-0.5061380
44	H	-6.8822760	-4.3918710	-1.9587310
45	H	-9.4650840	-3.5724120	1.3950160
46	H	-5.5996590	-1.7150110	1.1601130
47	C	-4.8370030	-2.8492110	-1.2807870
48	N	-4.1026180	-1.7356590	-1.0354790
49	O	-4.4750830	-3.7132810	-2.1341960
50	H	-4.3935580	-1.0340130	-0.3649030
51	C	-2.8034240	-1.4791570	-1.6617390
52	H	-2.2187500	-2.4073810	-1.6407880
53	C	-2.9398900	-1.0276850	-3.1329550

54	H	-3.4413390	-1.8098990	-3.7077300
55	H	-1.9505800	-0.8462780	-3.5619720
56	H	-3.5274370	-0.1056220	-3.1948740
57	C	-2.0589740	-0.4389280	-0.8457150
58	O	-0.8103170	-0.1872620	-1.1763910
59	N	-2.7195600	0.1732200	0.1281050
60	N	-1.8967360	1.1258900	0.7681490
61	C	-2.4152380	1.7927620	1.7656230
62	H	-3.4493170	1.5674280	2.0281530
63	C	-1.7324680	2.7919660	2.5419650
64	C	-0.3660130	3.1711800	2.3188660
65	C	-2.4636400	3.4209100	3.5840230
66	C	0.1930850	4.1673300	3.1600030
67	C	-1.8936720	4.3932700	4.3922190
68	H	-3.4968330	3.1218350	3.7401500
69	C	-0.5504170	4.7637750	4.1704230
70	H	1.2260310	4.4485290	2.9852100
71	H	-2.4703380	4.8611980	5.1821830
72	H	-0.0907760	5.5235680	4.7955640
73	O	0.4067820	2.6383880	1.3631820
74	H	9.0419150	-4.7434020	0.7613640
75	H	7.7306900	-2.0601440	-2.3428280
76	H	-9.0402900	-4.7461030	-0.7600520
77	H	-7.7314640	-2.0594050	2.3421720

(Λ, *S*)-**10b** in THF
Total energy = -4180.464803 Hartrees
Number of negative eigenvalues: 0

No	Symbol	X	Y	Z
1	Ga	-1.7828690	0.0000410	-0.0000090
2	C	5.2941480	-6.1166980	-0.1120020
3	C	4.0587300	-6.4891830	0.4325640
4	C	3.1537110	-5.5103510	0.8539490
5	C	3.4790120	-4.1467520	0.7314920
6	C	4.7135680	-3.7797260	0.1672240
7	C	5.6186390	-4.7599770	-0.2457000
8	H	5.9958110	-6.8780190	-0.4364240
9	H	2.1864290	-5.8155930	1.2400610
10	H	4.9526080	-2.7275160	0.0645060
11	C	2.5472900	-3.0625520	1.1568910
12	N	1.6641370	-3.3407770	2.1431660
13	O	2.5907190	-1.9140580	0.6116080
14	H	1.7114570	-4.2390190	2.6015490
15	C	0.6500410	-2.3873590	2.6495550
16	H	-0.0228460	-2.9997100	3.2587150
17	C	1.2728720	-1.3055380	3.5417770
18	H	0.4945860	-0.6433940	3.9299390
19	H	1.7849760	-1.7783730	4.3855490
20	H	1.9937580	-0.7002250	2.9879020
21	C	-0.2057510	-1.8246680	1.5104220
22	O	-0.2426270	-0.5111040	1.3110910
23	N	-0.8943320	-2.6981350	0.7996310
24	N	-1.6838100	-2.0372790	-0.1750850
25	C	-2.3186490	-2.7954580	-1.0307920
26	H	-2.1602100	-3.8709030	-0.9473380
27	C	-3.2013800	-2.3284240	-2.0623730
28	C	-3.5449140	-0.9468340	-2.2396790
29	C	-3.7625560	-3.2987830	-2.9340760
30	C	-4.4339810	-0.6109930	-3.2918180
31	C	-4.6289260	-2.9448710	-3.9567980
32	H	-3.4954640	-4.3415270	-2.7833780
33	C	-4.9617210	-1.5843990	-4.1299500
34	H	-4.6886450	0.4356030	-3.4172650
35	H	-5.0457170	-3.7007340	-4.6129270
36	H	-5.6397970	-1.2933870	-4.9267530
37	O	-3.0805770	0.0384310	-1.4592030
38	C	4.7136740	3.7795640	-0.1671030
39	C	5.6187830	4.7598080	0.2457550
40	C	5.2943890	6.1165340	0.1118700
41	C	4.0590290	6.4890330	-0.4328210
42	C	3.1539730	5.5102070	-0.8541410
43	C	3.4791780	4.1466030	-0.7314930
44	H	4.9526400	2.7273510	-0.0642360
45	H	5.9960800	6.8778490	0.4362400
46	H	2.1867340	5.8154570	-1.2403560
47	C	2.5474020	3.0624220	-1.1568180
48	N	1.6643470	3.3406070	-2.1431940
49	O	2.5906650	1.9140080	-0.6113550
50	H	1.7118110	4.2387770	-2.6017060
51	C	0.6502010	2.3872260	-2.6495470
52	H	-0.0226550	2.9995860	-3.2587320
53	C	1.2729820	1.3053450	-3.5417320
54	H	0.4946710	0.6432050	-3.9298510
55	H	1.7850860	1.7781260	-4.3855350
56	H	1.9938650	0.7000340	-2.9878490
57	C	-0.2056190	1.8246210	-1.5103940
58	O	-0.2426230	0.5110600	-1.3110670
59	N	-0.8941260	2.6981530	-0.7996130
60	N	-1.6836610	2.0373510	0.1750910
61	C	-2.3184310	2.7955700	1.0308120
62	H	-2.1598980	3.8710020	0.9473750
63	C	-3.2011990	2.3285940	2.0623880
64	C	-3.5448470	0.9470300	2.2396700
65	C	-3.7622940	3.2989840	2.9341080
66	C	-4.4339440	0.6112440	3.2918020
67	C	-4.6286950	2.9451270	3.9568220
68	H	-3.4951140	4.3417090	2.7834290
69	C	-4.9616050	1.5846790	4.1299490
70	H	-4.6886960	-0.4353330	3.4172290
71	H	-5.0454240	3.7010120	4.6129650
72	H	-5.6397070	1.2937100	4.9267460
73	O	-3.0805860	-0.0382600	1.4591790
74	H	6.5729480	-4.4681490	-0.6710430
75	H	3.7959360	-7.5378760	0.5205560
76	H	6.5730470	4.4679710	0.6711940
77	H	3.7963100	7.5377310	-0.5209600
78	Na	1.6480040	-0.0000770	-0.0000180

(Δ, *S*)-**10b** in THF
Total energy = -4180.471449 Hartrees
Number of negative eigenvalues: 0

No	Symbol	X	Y	Z
1	Ga	1.7039100	-0.0090000	0.0006510
2	C	-5.3156810	-6.1771110	-0.4033760
3	C	-4.0808410	-6.5255160	0.1583620
4	C	-3.2198030	-5.5314130	0.6322820
5	C	-3.5889460	-4.1761480	0.5460350
6	C	-4.8223940	-3.8325760	-0.0353420
7	C	-5.6835310	-4.8284940	-0.5011090
8	H	-5.9830890	-6.9506570	-0.7685590
9	H	-2.2525220	-5.8185280	1.0319650
10	H	-5.0947620	-2.7861310	-0.1090810
11	C	-2.7090580	-3.0764700	1.0316240
12	N	-1.8116940	-3.3532670	2.0049380
13	O	-2.8038810	-1.8986900	0.5558610
14	H	-1.7748180	-4.2770850	2.4107120
15	C	-0.8844400	-2.3362050	2.5271840
16	H	-1.4717330	-1.4702840	2.8506100
17	C	-0.1139450	-2.9156140	3.7238290
18	H	-0.8066000	-3.2260920	4.5133400
19	H	0.5576910	-2.1580940	4.1365810
20	H	0.4896170	-3.7739220	3.4121390
21	C	0.0625580	-1.8255840	1.4454890
22	O	0.1531690	-0.5054440	1.2957170
23	N	0.7398210	-2.7042070	0.7348740
24	N	1.5874730	-2.0460120	-0.1931870
25	C	2.2488580	-2.8074640	-1.0254750
26	H	2.0789430	-3.8817900	-0.9504520
27	C	3.1725640	-2.3454310	-2.0225400
28	C	3.5121840	-0.9630130	-2.2019860
29	C	3.7743350	-3.3209750	-2.8609210
30	C	4.4395050	-0.6319580	-3.2223960
31	C	4.6784690	-2.9717050	-3.8518890

32	H	3.5083830	-4.3639220	-2.7096180
33	C	5.0077420	-1.6104760	-4.0271710
34	H	4.6903860	0.4152410	-3.3500230
35	H	5.1267020	-3.7314020	-4.4823580
36	H	5.7149140	-1.3231270	-4.7996510
37	O	3.0066130	0.0274740	-1.4554000
38	C	-4.7823330	3.8820620	0.0284060
39	C	-5.6380040	4.8809920	0.4977310
40	C	-5.2575290	6.2271530	0.4158800
41	C	-4.0156510	6.5699770	-0.1336410
42	C	-3.1599720	5.5729220	-0.6110770
43	C	-3.5416420	4.2202120	-0.5406670
44	H	-5.0643480	2.8373890	0.0899170
45	H	-5.9207100	7.0030290	0.7838090
46	H	-2.1875060	5.8555640	-1.0012860
47	C	-2.6687170	3.1168900	-1.0307540
48	N	-1.7589850	3.3948100	-1.9922790
49	O	-2.7815070	1.9347550	-0.5700040
50	H	-1.7077130	4.3229140	-2.3865330
51	C	-0.8409570	2.3724940	-2.5211220
52	H	-1.4371660	1.5157060	-2.8522850
53	C	-0.0628840	2.9535110	-3.7119760
54	H	-0.7512730	3.2783180	-4.4994460
55	H	0.6007370	2.1921200	-4.1305720
56	H	0.5499860	3.8023570	-3.3927090
57	C	0.0988900	1.8421470	-1.4424370
58	O	0.1649460	0.5201830	-1.2955330
59	N	0.7946220	2.7061530	-0.7317440
60	N	1.6314130	2.0300060	0.1933600
61	C	2.3134860	2.7772940	1.0217770
62	H	2.1678180	3.8551140	0.9457210
63	C	3.2313050	2.2959130	2.0151710
64	C	3.5386350	0.9062340	2.1973430
65	C	3.8608860	3.2593000	2.8471770
66	C	4.4639850	0.5562250	3.2132170
67	C	4.7623020	2.8915410	3.8339080
68	H	3.6187770	4.3078100	2.6943460
69	C	5.0602430	1.5233830	4.0114180
70	H	4.6902890	-0.4963230	3.3428640
71	H	5.2321330	3.6422130	4.4594480
72	H	5.7650180	1.2216560	4.7806000
73	O	3.0036820	-0.0745080	1.4583770
74	H	-6.6373450	-4.5550040	-0.9394900
75	H	-3.7845240	-7.5671360	0.2195390
76	H	-6.5973610	4.6117350	0.9265420
77	H	-3.7098100	7.6094800	-0.1826530
78	Na	-1.7901630	0.0120650	-0.0095670

(Λ, *S*)-**10b**-Na in THF

Total energy = -4018.177281 Hartrees

Number of negative eigenvalues: 0

No	Symbol	X	Y	Z
1	Ga	0.0024850	1.0328520	-0.0022030
2	C	9.1958750	-2.2415000	0.8150580
3	C	8.0262650	-2.0474200	1.5606650
4	C	6.7767700	-2.0925940	0.9332210
5	C	6.6860090	-2.3324800	-0.4497100
6	C	7.8636020	-2.5419320	-1.1876590
7	C	9.1111940	-2.4912060	-0.5613870
8	H	10.1643130	-2.2041840	1.3035860
9	H	5.8807030	-1.9696760	1.5326340
10	H	7.7796350	-2.7412550	-2.2497700
11	C	5.3803560	-2.3994090	-1.1837940
12	N	4.3089680	-1.8124580	-0.5932020
13	O	5.2949690	-2.9696000	-2.3113590
14	H	4.3982520	-1.2327300	0.2347120
15	C	2.9774170	-1.7895240	-1.2056000
16	H	3.0843980	-1.5247050	-2.2657510
17	C	2.2723980	-3.1601100	-1.1207030
18	H	1.2811040	-3.0985150	-1.5780570
19	H	2.8671710	-3.9078290	-1.6509630
20	H	2.1609560	-3.4655660	-0.0750520
21	C	2.1474430	-0.7090120	-0.5381050
22	O	0.9551180	-0.4644420	-1.0342750
23	N	2.6683890	-0.0935820	0.5156660
24	N	1.7714930	0.8680560	1.0266840
25	C	2.1450190	1.5343710	2.0868130
26	H	3.1288150	1.3021720	2.4961130
27	C	1.3648530	2.5397650	2.7558000
28	C	0.0492120	2.9294100	2.3334650
29	C	1.9390540	3.1631100	3.8943290
30	C	-0.6201950	3.9302780	3.0848020
31	C	1.2641040	4.1401180	4.6108240
32	H	2.9359440	2.8557400	4.2003230
33	C	-0.0291900	4.5208460	4.1937950
34	H	-1.6138540	4.2172120	2.7582130
35	H	1.7216920	4.6037580	5.4778070
36	H	-0.5702370	5.2840810	4.7457130
37	O	-0.5770220	2.4043680	1.2753400
38	C	-7.8884560	-2.5116620	1.1889860
39	C	-9.1267470	-2.4852360	0.5430560
40	C	-9.1926670	-2.2643040	-0.8393270
41	C	-8.0136340	-2.0746900	-1.5710970
42	C	-6.7735090	-2.0956280	-0.9241080
43	C	-6.7016760	-2.3064070	0.4646800
44	H	-7.8191150	-2.6889040	2.2560430
45	H	-10.153844	-2.2460510	-1.3430470
46	H	-5.8694700	-1.9772440	-1.5123480
47	C	-5.4063170	-2.3479410	1.2186870
48	N	-4.3307130	-1.7657310	0.6310650
49	O	-5.3327020	-2.8946870	2.3586370
50	H	-4.4118800	-1.2035650	-0.2096470
51	C	-3.0058760	-1.7289230	1.2570340
52	H	-3.1233670	-1.4395510	2.3096490
53	C	-2.3013190	-3.1017000	1.2103200
54	H	-1.3141400	-3.0301900	1.6749820
55	H	-2.9018320	-3.8366830	1.7517190
56	H	-2.1805740	-3.4305890	0.1728130
57	C	-2.1669630	-0.6660670	0.5727290
58	O	-0.9766570	-0.4175790	1.0713880
59	N	-2.6772400	-0.0711260	-0.4979600
60	N	-1.7693300	0.8705660	-1.0266480
61	C	-2.1363930	1.5234370	-2.0973220
62	H	-3.1246530	1.2983980	-2.4997990
63	C	-1.3442460	2.5062090	-2.7855850
64	C	-0.0192640	2.8796890	-2.3780820
65	C	-1.9160730	3.1227720	-3.9290320
66	C	0.6609040	3.8588800	-3.1480750
67	C	-1.2305390	4.0786400	-4.6637300
68	H	-2.9199780	2.8275040	-4.2238300
69	C	0.0716960	4.4438550	-4.2609700
70	H	1.6613880	4.1340000	-2.8323850
71	H	-1.6867430	4.5376560	-5.5338970
72	H	0.6211470	5.1905540	-4.8270240
73	O	0.6071760	2.3576950	-1.3184680
74	H	10.0143600	-2.6464210	-1.1426520
75	H	8.0851730	-1.8684160	2.6293020
76	H	-10.037244	-2.6371300	1.1136640
77	H	-8.0578270	-1.9182320	-2.6439740

(Δ, *S*)-**10b**-Na in THF

Total energy = -4018.177053 Hartrees

Number of negative eigenvalues: 0

No	Symbol	X	Y	Z
1	Ga	0.0000100	1.2760430	0.0002120
2	C	-8.5466380	-3.3778190	0.8708600
3	C	-7.5627460	-2.5346080	1.4024790
4	C	-6.3571060	-2.3422540	0.7196570
5	C	-6.1241720	-2.9945630	-0.5046560
6	C	-7.1102020	-3.8511780	-1.0231880
7	C	-8.3163910	-4.0377650	-0.3437770
8	H	-9.4819070	-3.5239130	1.4017490
9	H	-5.5960970	-1.7086550	1.1639850

10	H	-6.9122820	-4.3609860	-1.9589910
11	C	-4.8511430	-2.8427840	-1.2827410
12	N	-4.1051030	-1.7382190	-1.0336070
13	O	-4.5035910	-3.7073460	-2.1404700
14	H	-4.3837580	-1.0378960	-0.3561250
15	C	-2.8026940	-1.4915480	-1.6574940
16	H	-2.2220410	-2.4221270	-1.6300910
17	C	-2.9313490	-1.0464570	-3.1309450
18	H	-3.4329160	-1.8297650	-3.7042410
19	H	-1.9387410	-0.8694780	-3.5539610
20	H	-3.5146080	-0.1220790	-3.1990640
21	C	-2.0576140	-0.4499620	-0.8431790
22	O	-0.8109360	-0.1983920	-1.1745840
23	N	-2.7194030	0.1618770	0.1312020
24	N	-1.8975930	1.1144000	0.7699660
25	C	-2.4139990	1.7826660	1.7667130
26	H	-3.4481590	1.5579110	2.0304350
27	C	-1.7298880	2.7823540	2.5414460
28	C	-0.3628580	3.1597520	2.3159920
29	C	-2.4588780	3.4131380	3.5832350
30	C	0.1971840	4.1567810	3.1567190
31	C	-1.8880200	4.3858830	4.3903030
32	H	-3.4924610	3.1150130	3.7403790
33	C	-0.5445340	4.7547440	4.1667930
34	H	1.2301800	4.4360710	2.9797500
35	H	-2.4638960	4.8551140	5.1803500
36	H	-0.0834980	5.5148810	4.7908810
37	O	0.4077810	2.6273990	1.3615840
38	C	7.1110190	-3.8501300	1.0235470
39	C	8.3173210	-4.0364440	0.3442630
40	C	8.5475730	-3.3763890	-0.8703120
41	C	7.5635730	-2.5333460	-1.4020020
42	C	6.3578170	-2.3412670	-0.7193100
43	C	6.1248760	-2.9936880	0.5049470
44	H	6.9130910	-4.3600120	1.9593070
45	H	9.4829310	-3.5222690	-1.4011040
46	H	5.5967300	-1.7078010	-1.1636890
47	C	4.8517350	-2.8421960	1.2829080
48	N	4.1054680	-1.7377940	1.0337210
49	O	4.5043080	-3.7068220	2.1406240
50	H	4.3839970	-1.0374220	0.3562410
51	C	2.8029420	-1.4914630	1.6574940
52	H	2.2225240	-2.4221900	1.6299900
53	C	2.9313340	-1.0464120	3.1309670
54	H	3.4330600	-1.8296170	3.7042620
55	H	1.9386370	-0.8697120	3.5538870
56	H	3.5143370	-0.1218800	3.1992040
57	C	2.0576860	-0.4500300	0.8431440
58	O	0.8109690	-0.1986780	1.1745640
59	N	2.7193740	0.1619120	-0.1312470
60	N	1.8974290	1.1143800	-0.7699380
61	C	2.4134410	1.7821420	-1.7672250
62	H	3.4474090	1.5570590	-2.0314180
63	C	1.7291020	2.7816220	-2.5420300
64	C	0.3623410	3.1595670	-2.3158500
65	C	2.4575640	3.4115870	-3.5846820
66	C	-0.1979760	4.1562950	-3.1567500
67	C	1.8864390	4.3840340	-4.3919200
68	H	3.4909470	3.1130540	-3.7423700
69	C	0.5432230	4.7534400	-4.1676880
70	H	-1.2307600	4.4360090	-2.9792120
71	H	2.4619050	4.8526240	-5.1826460
72	H	0.0819890	5.5133580	-4.7918980
73	O	-0.4077500	2.6281090	-1.3604950
74	H	-9.0736870	-4.6956590	-0.7577290
75	H	-7.7297040	-2.0331850	2.3502030
76	H	9.0747040	-4.6942070	0.7582660
77	H	7.7305450	-2.0318400	-2.3496790

(Λ, *S*)-**10b** in DMSO
Total energy = -4180.473336 Hartrees
Number of negative eigenvalues: 0

No	Symbol	X	Y	Z
1	Ga	1.7736560	0.0000040	0.0000200
2	C	-5.2754810	6.1545930	-0.1107560
3	C	-4.0481260	6.5214790	0.4558320
4	C	-3.1455440	5.5384090	0.8729290
5	C	-3.4652540	4.1759770	0.7236250
6	C	-4.6920640	3.8147890	0.1384180
7	C	-5.5947410	4.7992240	-0.2701670
8	H	-5.9752000	6.9191790	-0.4314850
9	H	-2.1854470	5.8403330	1.2786420
10	H	-4.9287960	2.7640100	0.0171710
11	C	-2.5350980	3.0881170	1.1419830
12	N	-1.6620620	3.3525470	2.1405620
13	O	-2.5673820	1.9481470	0.5766750
14	H	-1.7217920	4.2382710	2.6218990
15	C	-0.6585920	2.3889570	2.6458940
16	H	0.0163920	2.9921430	3.2619260
17	C	-1.2940650	1.3085620	3.5312550
18	H	-0.5227330	0.6421700	3.9264070
19	H	-1.8088700	1.7843050	4.3714580
20	H	-2.0151510	0.7081780	2.9720960
21	C	0.1971760	1.8254220	1.5072130
22	O	0.2428230	0.5106750	1.3151650
23	N	0.8817920	2.6979540	0.7902600
24	N	1.6742700	2.0359640	-0.1817870
25	C	2.3054910	2.7929020	-1.0416730
26	H	2.1421420	3.8680190	-0.9651470
27	C	3.1922600	2.3239070	-2.0697860
28	C	3.5445070	0.9437410	-2.2386740
29	C	3.7498650	3.2926180	-2.9459190
30	C	4.4385030	0.6064820	-3.2854050
31	C	4.6214310	2.9373480	-3.9641880
32	H	3.4761440	4.3344790	-2.8028030
33	C	4.9632120	1.5782350	-4.1283790
34	H	4.6998110	-0.4392330	-3.4056160
35	H	5.0350410	3.6916690	-4.6238820
36	H	5.6448470	1.2863790	-4.9216100
37	O	3.0826130	-0.0407480	-1.4520960
38	C	-4.6921500	-3.8147370	-0.1383910
39	C	-5.5948220	-4.7991660	0.2702170
40	C	-5.2755540	-6.1545380	0.1108420
41	C	-4.0481970	-6.5214340	-0.4557360
42	C	-3.1456200	-5.5383690	-0.8728560
43	C	-3.4653360	-4.1759360	-0.7235840
44	H	-4.9288870	-2.7639560	-0.0171740
45	H	-5.9752720	-6.9191190	0.4315890
46	H	-2.1855230	-5.8402950	-1.2785700
47	C	-2.5351820	-3.0880920	-1.1419810
48	N	-1.6621080	-3.3525960	-2.1405070
49	O	-2.5674390	-1.9481120	-0.5766900
50	H	-1.7218350	-4.2383510	-2.6217910
51	C	-0.6585930	-2.3890620	-2.6458570
52	H	0.0164020	-2.9923050	-3.2618220
53	C	-1.2940020	-1.3087140	-3.5313220
54	H	-0.5226380	-0.6423620	-3.9264790
55	H	-1.8087740	-1.7845010	-4.3715190
56	H	-2.0151040	-0.7082800	-2.9722380
57	C	0.1971440	-1.8254730	-1.5071800
58	O	0.2427840	-0.5107150	-1.3152050
59	N	0.8817960	-2.6979640	-0.7902100
60	N	1.6742950	-2.0359590	0.1818160
61	C	2.3055600	-2.7928940	1.0416730
62	H	2.1422190	-3.8680120	0.9651510
63	C	3.1923730	-2.3238950	2.0697480
64	C	3.5446260	-0.9437280	2.2386180
65	C	3.7500240	-3.2926060	2.9458520
66	C	4.4386720	-0.6064680	3.2853070
67	C	4.6216390	-2.9373340	3.9640790
68	H	3.4762990	-4.3344670	2.8027480
69	C	4.9634240	-1.5782210	4.1282540
70	H	4.6999840	0.4392470	3.4055060
71	H	5.0352820	-3.6916550	4.6237510
72	H	5.6450970	-1.2863640	4.9214530
73	O	3.0826900	0.0407610	1.4520660
74	H	-6.5431490	4.5117590	-0.7112450
75	H	-3.7898850	7.5692090	0.5652810
76	H	-6.5432330	-4.5116950	0.7112850
77	H	-3.7899520	-7.5691650	-0.5651620
78	Na	-1.6654750	0.0000020	0.0000010

(Δ, *S*)-**10b** in DMSO
Total energy = -4180.479852 Hartrees
Number of negative eigenvalues: 0

No	Symbol	X	Y	Z
1	Ga	1.6918590	-0.0765550	-0.0027770
2	C	-5.5320840	-6.0324450	-0.3860530
3	C	-4.3194350	-6.4121110	0.2035170
4	C	-3.4276260	-5.4389710	0.6641380
5	C	-3.7434390	-4.0733540	0.5361800
6	C	-4.9550430	-3.6990120	-0.0720580
7	C	-5.8470510	-4.6738550	-0.5245330
8	H	-6.2235360	-6.7896390	-0.7404470
9	H	-2.4789740	-5.7517950	1.0881040
10	H	-5.1884030	-2.6456960	-0.1759360
11	C	-2.8279020	-2.9963840	1.0055720
12	N	-1.9547030	-3.2823220	1.9978510
13	O	-2.8673430	-1.8288840	0.4963650
14	H	-1.9697780	-4.1907380	2.4388660
15	C	-1.0028180	-2.2858560	2.5125610
16	H	-1.5651390	-1.3967820	2.8176890
17	C	-0.2608250	-2.8689750	3.7258770
18	H	-0.9700610	-3.1406460	4.5145350
19	H	0.4326580	-2.1272010	4.1313760
20	H	0.3133380	-3.7544910	3.4355730
21	C	-0.0297950	-1.8207070	1.4337410
22	O	0.1228330	-0.5057150	1.2858000
23	N	0.6126110	-2.7279660	0.7257990
24	N	1.4948950	-2.1073010	-0.1967070
25	C	2.1265940	-2.8966070	-1.0262840
26	H	1.9081710	-3.9623100	-0.9561350
27	C	3.0782820	-2.4739780	-2.0153210
28	C	3.4830940	-1.1087250	-2.1886230
29	C	3.6417540	-3.4747260	-2.8508920
30	C	4.4341790	-0.8184660	-3.1986910
31	C	4.5705170	-3.1652070	-3.8327100
32	H	3.3263810	-4.5044070	-2.7050960
33	C	4.9643950	-1.8207110	-4.0013860
34	H	4.7350310	0.2160780	-3.3227270
35	H	4.9884620	-3.9434150	-4.4611710
36	H	5.6910270	-1.5645020	-4.7664920
37	O	3.0165070	-0.0960240	-1.4422190
38	C	-4.5736700	4.1282750	0.0664690
39	C	-5.3809860	5.1710120	0.5263560
40	C	-4.9536260	6.4994260	0.3968460
41	C	-3.7136450	6.7804180	-0.1911190
42	C	-2.9063040	5.7393070	-0.6590500
43	C	-3.3351140	4.4040910	-0.5402830
44	H	-4.8934360	3.0972800	0.1638610
45	H	-5.5792200	7.3093780	0.7569060
46	H	-1.9348660	5.9749810	-1.0810390
47	C	-2.5147840	3.2558670	-1.0170990
48	N	-1.6156380	3.4718610	-2.0040460
49	O	-2.6602100	2.0920110	-0.5190340
50	H	-1.5475440	4.3819530	-2.4365400
51	C	-0.7559410	2.3985530	-2.5273360
52	H	-1.3958160	1.5679110	-2.8442020
53	C	0.0403740	2.9248640	-3.7321050
54	H	-0.6381480	3.2688410	-4.5195870
55	H	0.6646810	2.1273190	-4.1441090
56	H	0.6919220	3.7511480	-3.4309340
57	C	0.1658690	1.8343390	-1.4507410
58	O	0.1935090	0.5096060	-1.3141500
59	N	0.8875970	2.6710720	-0.7323950
60	N	1.6982890	1.9631220	0.1927510
61	C	2.3985950	2.6839830	1.0297170
62	H	2.2899660	3.7663570	0.9582870
63	C	3.2915050	2.1672090	2.0288270
64	C	3.5496420	0.7676150	2.2088290
65	C	3.9459250	3.1057720	2.8702540
66	C	4.4533780	0.3819840	3.2304360
67	C	4.8257600	2.7033670	3.8634050
68	H	3.7406680	4.1623210	2.7204300
69	C	5.0755960	1.3255300	4.0381800
70	H	4.6426240	-0.6780830	3.3596830
71	H	5.3147910	3.4352050	4.4962760
72	H	5.7623500	0.9969490	4.8124150
73	O	2.9870850	-0.1924970	1.4594510
74	H	-6.7838610	-4.3763710	-0.9833300
75	H	-4.0644200	-7.4620950	0.2976640
76	H	-6.3392090	4.9497630	0.9840450
77	H	-3.3718670	7.8060510	-0.2783350
78	Na	-1.8180040	0.0757700	-0.0377970

(Λ, *S*)-**10b**-Na in DMSO
Total energy = -4018.190724 Hartrees
Number of negative eigenvalues: 0

No	Symbol	X	Y	Z
1	Ga	-0.0002000	1.0101520	0.0001990
2	C	9.2365080	-2.1968500	0.8387080
3	C	8.0616990	-2.0451140	1.5863330
4	C	6.8150920	-2.0836390	0.9527500
5	C	6.7325720	-2.2743470	-0.4386460
6	C	7.9157480	-2.4411890	-1.1792150
7	C	9.1603790	-2.3974000	-0.5463810
8	H	10.2024420	-2.1649550	1.3321710
9	H	5.9161290	-1.9944960	1.5536790
10	H	7.8402090	-2.6016100	-2.2485280
11	C	5.4311080	-2.3313210	-1.1785420
12	N	4.3461270	-1.7884840	-0.5698700
13	O	5.3557590	-2.8540440	-2.3304970
14	H	4.4218720	-1.2436790	0.2821150
15	C	3.0192110	-1.7712740	-1.1912950
16	H	3.1350950	-1.4990910	-2.2483480
17	C	2.3257330	-3.1492500	-1.1219620
18	H	1.3415600	-3.0979400	-1.5957510
19	H	2.9350810	-3.8902750	-1.6451090
20	H	2.2017360	-3.4604680	-0.0794710
21	C	2.1720160	-0.7042630	-0.5251710
22	O	0.9849930	-0.4617880	-1.0394480
23	N	2.6678050	-0.0987770	0.5452850
24	N	1.7521300	0.8481920	1.0543740
25	C	2.1036140	1.5033070	2.1296730
26	H	3.0829070	1.2749570	2.5510760
27	C	1.3036790	2.4923030	2.7996150
28	C	-0.0091970	2.8757110	2.3642670
29	C	1.8564730	3.1060620	3.9545220
30	C	-0.6983330	3.8605240	3.1174730
31	C	1.1621670	4.0678960	4.6730910
32	H	2.8513610	2.8039840	4.2711140
33	C	-0.1282750	4.4426240	4.2425530
34	H	-1.6900830	4.1440340	2.7818760
35	H	1.6030760	4.5246200	5.5520150
36	H	-0.6837820	5.1940720	4.7957830
37	O	-0.6167420	2.3570350	1.2888220
38	C	-7.9142140	-2.4429460	1.1795390
39	C	-9.1595310	-2.3975350	0.5481770
40	C	-9.2371050	-2.1949710	-0.8365380
41	C	-8.0630510	-2.0428400	-1.5852700
42	C	-6.8157520	-2.0829840	-0.9531510
43	C	-6.7317740	-2.2757290	0.4378760
44	H	-7.8375450	-2.6049250	2.2485370
45	H	-10.203575	-2.1618120	-1.3288660
46	H	-5.9174230	-1.9934920	-1.5549780
47	C	-5.4295340	-2.3344980	1.1762600
48	N	-4.3449230	-1.7912910	0.5672560
49	O	-5.3532240	-2.8589080	2.3273860
50	H	-4.4212950	-1.2452310	-0.2838690
51	C	-3.0174480	-1.7753690	1.1875160
52	H	-3.1324020	-1.5053880	2.2452250
53	C	-2.3240410	-3.1532050	1.1146820
54	H	-1.3394780	-3.1029340	1.5877800
55	H	-2.9329570	-3.8953460	1.6367590
56	H	-2.2009010	-3.4622210	0.0714360
57	C	-2.1709030	-0.7069650	0.5228390
58	O	-0.9837530	-0.4649380	1.0370370
59	N	-2.6673430	-0.0999950	-0.5464830

60	N	-1.7523030	0.8482720	-1.0543030
61	C	-2.1040020	1.5042220	-2.1290220
62	H	-3.0829370	1.2754250	-2.5510140
63	C	-1.3047060	2.4946050	-2.7976790
64	C	0.0075140	2.8790280	-2.3612430
65	C	-1.8574550	3.1087200	-3.9524180
66	C	0.6961040	3.8651120	-3.1132790
67	C	-1.1636900	4.0718030	-4.6698360
68	H	-2.8518480	2.8058820	-4.2698340
69	C	0.1261260	4.4474920	-4.2382560
70	H	1.6873660	4.1493680	-2.7768740
71	H	-1.6045390	4.5287630	-5.5486680
72	H	0.6812040	5.1999120	-4.7905950
73	O	0.6148580	2.3602550	-1.2857310
74	H	10.0675150	-2.5194840	-1.1290660
75	H	8.1145330	-1.9044700	2.6608380
76	H	-10.066081	-2.5199200	1.1317120
77	H	-8.1170170	-1.9006290	-2.6595110

(Δ, *S*)-**10b**-Na in DMSO
Total energy = -4018.190421 Hartrees
Number of negative eigenvalues: 0

No	Symbol	X	Y	Z
1	Ga	0.0000280	1.2902710	-0.0001040
2	C	8.5251270	-3.4281610	-0.8644390
3	C	7.5548040	-2.5678660	-1.3941870
4	C	6.3503720	-2.3615360	-0.7133340
5	C	6.1050810	-3.0168900	0.5072710
6	C	7.0777060	-3.8902680	1.0241010
7	C	8.2827250	-4.0909360	0.3464310
8	H	9.4594250	-3.5848190	-1.3936610
9	H	5.6006710	-1.7140680	-1.1568070
10	H	6.8726290	-4.4014840	1.9576170
11	C	4.8330330	-2.8503760	1.2817780
12	N	4.1011990	-1.7350080	1.0370150
13	O	4.4684680	-3.7140130	2.1346150
14	H	4.3940050	-1.0335360	0.3671020
15	C	2.8026280	-1.4762760	1.6636420
16	H	2.2170640	-2.4039620	1.6441560
17	C	2.9404760	-1.0232250	3.1342900
18	H	3.4416750	-1.8051830	3.7096240
19	H	1.9517120	-0.8406060	3.5641120
20	H	3.5289190	-0.1016460	3.1948670
21	C	2.0585690	-0.4364170	0.8468750
22	O	0.8097080	-0.1844130	1.1771100
23	N	2.7195190	0.1756700	-0.1266390
24	N	1.8969930	1.1285170	-0.7670980
25	C	2.4161090	1.7951370	-1.7645110
26	H	3.4502450	1.5696220	-2.0265700
27	C	1.7338050	2.7942800	-2.5413450
28	C	0.3673320	3.1737410	-2.3191100
29	C	2.4656250	3.4228870	-3.5832260
30	C	-0.1913240	4.1696560	-3.1606980
31	C	1.8960980	4.3950860	-4.3919290
32	H	3.4988430	3.1236900	-3.7387730
33	C	0.5527700	4.7657880	-4.1709470
34	H	-1.2243390	4.4510590	-2.9865840
35	H	2.4731950	4.8627500	-5.1817030
36	H	0.0935220	5.5254340	-4.7965150
37	O	-0.4061380	2.6411370	-1.3634870
38	C	-7.0782540	-3.8896200	-1.0242980
39	C	-8.2833960	-4.0900230	-0.3467680
40	C	-8.5257590	-3.4272560	0.8641140
41	C	-7.5552770	-2.5672350	1.3940140
42	C	-6.3507210	-2.3611680	0.7133000
43	C	-6.1054640	-3.0165160	-0.5073150
44	H	-6.8732090	-4.4008350	-1.9578220
45	H	-9.4601530	-3.5837090	1.3932280
46	H	-5.6009030	-1.7139130	1.1568870
47	C	-4.8332910	-2.8502760	-1.2816750
48	N	-4.1012600	-1.7350530	-1.0368390
49	O	-4.4688340	-3.7139660	-2.1345030
50	H	-4.3939950	-1.0335240	-0.3669550
51	C	-2.8025880	-1.4765670	-1.6633570
52	H	-2.2171860	-2.4043530	-1.6437980
53	C	-2.9402310	-1.0235250	-3.1340290
54	H	-3.4415070	-1.8054130	-3.7093910
55	H	-1.9513970	-0.8410800	-3.5637650
56	H	-3.5285150	-0.1018500	-3.1946790
57	C	-2.0584100	-0.4368220	-0.8465530
58	O	-0.8095500	-0.1848920	-1.1768430
59	N	-2.7193820	0.1755210	0.1267870
60	N	-1.8968520	1.1284800	0.7670790
61	C	-2.4158190	1.7949420	1.7646740
62	H	-3.4498460	1.5692280	2.0269910
63	C	-1.7334670	2.7941110	2.5414360
64	C	-0.3671570	3.1739140	2.3187910
65	C	-2.4650600	3.4223650	3.5836880
66	C	0.1915830	4.1697820	3.1603780
67	C	-1.8954570	4.3945260	4.3923830
68	H	-3.4981600	3.1229190	3.7395350
69	C	-0.5522840	4.7655550	4.1710060
70	H	1.2244740	4.4514450	2.9859510
71	H	-2.4723770	4.8619140	5.1824490
72	H	-0.0929780	5.5251730	4.7965660
73	O	0.4060610	2.6417360	1.3627240
74	H	9.0296830	-4.7611340	0.7589980
75	H	7.7315810	-2.0638030	-2.3385390
76	H	-9.0304780	-4.7600090	-0.7594530
77	H	-7.7320270	-2.0631810	2.3383760

[1] Shi, D.; Cao, J.; Weng, P.; Yan, X.; Zhao Lia, Z.; Jiang,Y.-B. Chalcogen bonding mediates the formation of supramolecular helices of azapeptides in crystals. *Org. Biomol. Chem*. **2021**, *19*, 6397–6401.
[2] Sheldrick, G. M. A short history of SHELX. *Acta Crystallogr. Sect. A* **2008**, *64*, 112–122.
[3] Dolomanov, O. V.; Bourhis, L. J.; Gildea, R. J.; Howard, J. A. K.; Puschmann, H. OLEX2: a complete structure solution, refinement and analysis program. *J. Appl. Crystallogr.* **2009**, *42*, 339–341.
[4] Gaussian 09, Revision E.01, Frisch, M. J.; Trucks, G. W.; Schlegel, H. B.; Scuseria, G. E.; Robb, M. A.; Cheeseman, J. R.; Scalmani, G.; Barone, V.; Petersson, G. A.; Nakatsuji, H.; Li, X.; Caricato, M.; Marenich, A. V.; Bloino, J.; Janesko, B. G.; Gomperts, R.; Mennucci, B.; Hratchian, H. P.; Ortiz, J. V.; Izmaylov, A. F.; Sonnenberg, J. L.; Williams-Young, D.; Ding, F.; Lipparini, F.; Egidi, F.; Goings, J.; Peng, B.; Petrone, A.; Henderson, T.; Ranasinghe, D.; Zakrzewski, V. G.; Gao, J.; Rega, N.; Zheng, G.; Liang, W.; Hada, M.; Ehara, M.; Toyota, K.; Fukuda, R.; Hasegawa, J.; Ishida, M.; Nakajima, T.; Honda, Y.; Kitao, O.; Nakai, H.; Vreven, T.; Throssell, K.; Montgomery, J. A., Jr.; Peralta, J. E.; Ogliaro, F.; Bearpark, M. J.; Heyd, J. J.; Brothers, E. N.; Kudin, K. N.; Staroverov, V. N.; Keith, T. A.; Kobayashi, R.; Normand, J.; Raghavachari, K.; Rendell, A. P.; Burant, J. C.; Iyengar, S. S.; Tomasi, J.; Cossi, M.; Millam, J. M.; Klene, M.; Adamo, C.; Cammi, R.; Ochterski, J. W.; Martin, R. L.; Morokuma, K.; Farkas, O.; Foresman, J. B.; Fox, D. J. Gaussian, Inc., Wallingford CT, 2009.
